# Programmed cell death 5 mediates HDAC3 decay to promote genotoxic stress response

**DOI:** 10.1038/ncomms8390

**Published:** 2015-06-16

**Authors:** Hyo-Kyoung Choi, Youngsok Choi, Eun Sung Park, Soo-Yeon Park, Seung-Hyun Lee, Jaesung Seo, Mi-Hyeon Jeong, Jae-Wook Jeong, Jae-Ho Jeong, Peter C. W. Lee, Kyung-Chul Choi, Ho-Geun Yoon

**Affiliations:** 1 Department of Biochemistry and Molecular Biology, Brain Korea 21 PLUS Project for Medical Sciences, Yonsei University College of Medicine, Seoul 120-752, Korea; 2 Department of Biomedical Science, CHA University, 335 Pangyo-ro, Bundang-gu, Seongnam-si, Gyeonggi-do 436-400, Korea; 3 Medical Convergence Research Institute, Yonsei University College of Medicine, Seoul 120-752, Korea; 4 Department of Obstetrics, Gynecology and Reproductive Biology, Michigan State University College of Human Medicine, Lansing, Michigan 49503, USA; 5 Department of Surgery, Yonsei University College of Medicine, Seoul 120-752, Korea; 6 Department of Biomedical Sciences, University of Ulsan College of Medicine, Seoul 138-736, Korea

## Abstract

The inhibition of p53 activity by histone deacetylase 3 (HDAC3) has been reported, but the precise molecular mechanism is unknown. Here we show that programmed cell death 5 (PDCD5) selectively mediates HDAC3 dissociation from p53, which induces HDAC3 cleavage and ubiquitin-dependent proteasomal degradation. Casein kinase 2 alpha phosphorylates PDCD5 at Ser-119 to enhance its stability and importin 13-mediated nuclear translocation of PDCD5. Genetic deletion of PDCD5 abrogates etoposide (ET)-induced p53 stabilization and HDAC3 cleavage, indicating an essential role of PDCD5 in p53 activation. Restoration of PDCD5^WT^ in *PDCD5*^*−/−*^ MEFs restores ET-induced HDAC3 cleavage. Reduction of both PDCD5 and p53, but not reduction of either protein alone, significantly enhances *in vivo* tumorigenicity of AGS gastric cancer cells and correlates with poor prognosis in gastric cancer patients. Our results define a mechanism for p53 activation via PDCD5-dependent HDAC3 decay under genotoxic stress conditions.

## Abstract (web summary)

The tumour suppressor p53 is known to be inhibited by histone deacetylase 3 but the molecular mechanism is poorly understood. Here Choi *et al*. show regulation by programmed cell death 5 and an essential role in activating p53 following DNA damage.

## Graphical abstract

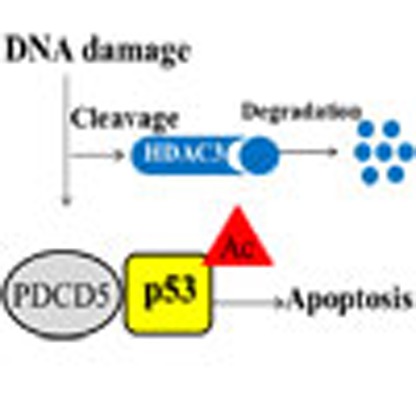

Deciphering the regulatory pathway for p53-dependent apoptosis, which is involved in a variety of stress signals, is important for understanding tumorigenesis and paving the way for new cancer therapies^1^. In response to a broad range of cellular stresses, p53 accumulates in the cell and thereby becomes activated, indicating that protein abundance dictates function^2^. It is generally believed that p53 protein accumulation is not due to an enhanced transcriptional response of the cell, but rather is the consequence of p53 protein stabilization resulting from post-translational modifications^3^. Acetylation of p53 at different lysine residues has been shown to produce a variety of effects on p53 function^4^. For example, acetylation-dependent p53 stabilization was shown to antagonize the mouse double minute 2 homology (MDM2)-mediated negative control of p53 (ref. 5). Conversely, deacetylation of p53, either by an HDAC1-containing complex or by the NAD-dependent histone deacetylase Sir2a, was shown to repress p53-dependent transcriptional activation, apoptosis and growth arrest. MDM2 was also shown to interfere with the acetylation of p53, and subsequently promote the HDAC1-mediated deacetylation of p53 (ref. 6). Thus, coordinated interplay between histone acetytransferases and histone deacetylases (HDACs) in the regulation of p53 acetylation is believed to play a significant role in p53-mediated apoptosis.

Recent studies highlight a connection between HDAC3 function and p53-mediated apoptosis. For instance, suppression of HDAC3 expression, or inhibition of its activity, was found to increase p53 stability and acetylation in human cancer cells^7^,^8^. Moreover, HDAC3 and p53 have been shown to interact indirectly via formation of a complex with the MAGE-A tumour antigen, which confers resistance to chemotherapeutic agents^9^. In accordance with the above finding, downregulation of HDAC3 decreases cancer cell death in response to anticancer drugs^10^. Stimuli such as osmotic stress or FAS ligand binding trigger the caspase-7-dependent C-terminal cleavage of HDAC3 in mammalian cells, eventually leading to apoptosis induction by modulation of HDAC3 activity^11^,^12^. Although these studies demonstrated the potential engagement of HDAC3 in caspase-7-dependent apoptosis, the effects of caspase-7-mediated cleavage on HDAC3 deacetylase activity are somewhat controversial. These findings suggest that apoptotic stimuli may alter the function of HDAC3 via C-terminal cleavage, presumably by leading to cellular apoptosis via activation of a pro-apoptotic molecule such as p53. Consistent with this hypothesis, a meta-analysis of human solid tumours showed that HDAC3 was one of the most frequently upregulated genes in cancer cells^13^. This suggests that cancer cells may resist apoptotic cell death, at least in part, through HDAC3-mediated mechanisms. However, detailed mechanisms underlying the role of HDAC3 in p53-mediated apoptosis have not been fully elucidated.

Programmed cell death 5 (PDCD5), also designated TFAR19 (TF-1 cell apoptosis-related gene-19), is a novel gene from TF-1 cells undergoing cytokine deprivation-induced apoptosis^14^. The amino-acid sequence of PDCD5 is quite conserved among eukaryotic species, indicating that PDCD5 has important biological functions in multiple organisms. PDCD5 is widely expressed in a variety of tissues, with messenger RNA (mRNA) levels in fetal tissue being significantly lower than that observed in adult tissues^15^. Accumulating evidence indicates that expression of PDCD5 is downregulated in human cancer patients, specifically those affected by lung cancer, ovarian cancer, gastric cancer and glioma, suggesting that decreased expression of PDCD5 may be associated with the pathogenesis of human tumours^16^,^17^,^18^. When overexpressed in cancer cell lines, PDCD5 facilitates apoptosis triggered by genotoxic stress^19^,^20^. PDCD5 protein is accumulated in cells undergoing apoptosis, and translocates rapidly from the cytoplasm to the nucleus of cells^21^. Recent studies also show that PDCD5 plays an important enhancing role in TAJ/TROY-triggered paraptosis-like cell death^22^. Sensitivity of HeLa cells to etoposide (ET)-induced apoptosis is reduced by *in situ* electroporation of anti-PDCD5 monoclonal antibody^23^. More recently, PDCD5 was shown to stabilize p53 by inhibiting p53–MDM2 interactions^24^. Although PDCD5 appears to behave as a p53 cofactor, the precise mechanisms behind PDCD5-mediated p53-dependent apoptosis remain unclear.

In this study, we demonstrate that PDCD5 promotes genotoxic stress-induced p53 acetylation through direct mediating dissociation of HDAC3 from p53, leading to caspase-3-dependent HDAC3 cleavage. Furthermore, we reveal that PDCD5 is required for p53 stabilization and cleavage of HDAC3 *in vivo*. Finally, we verify the positive correlation between PDCD5 and p53 in human gastric cancer patients and *in vivo* tumorigenicity of gastric cancer cells. Our findings highlight the functional importance of PDCD5 in genotoxic stress-induced p53 activation through its HDAC3 decay-mediating activity.

## Results

### PDCD5 induces caspase-3-mediated cleavage of HDAC3

**Figure 1:**
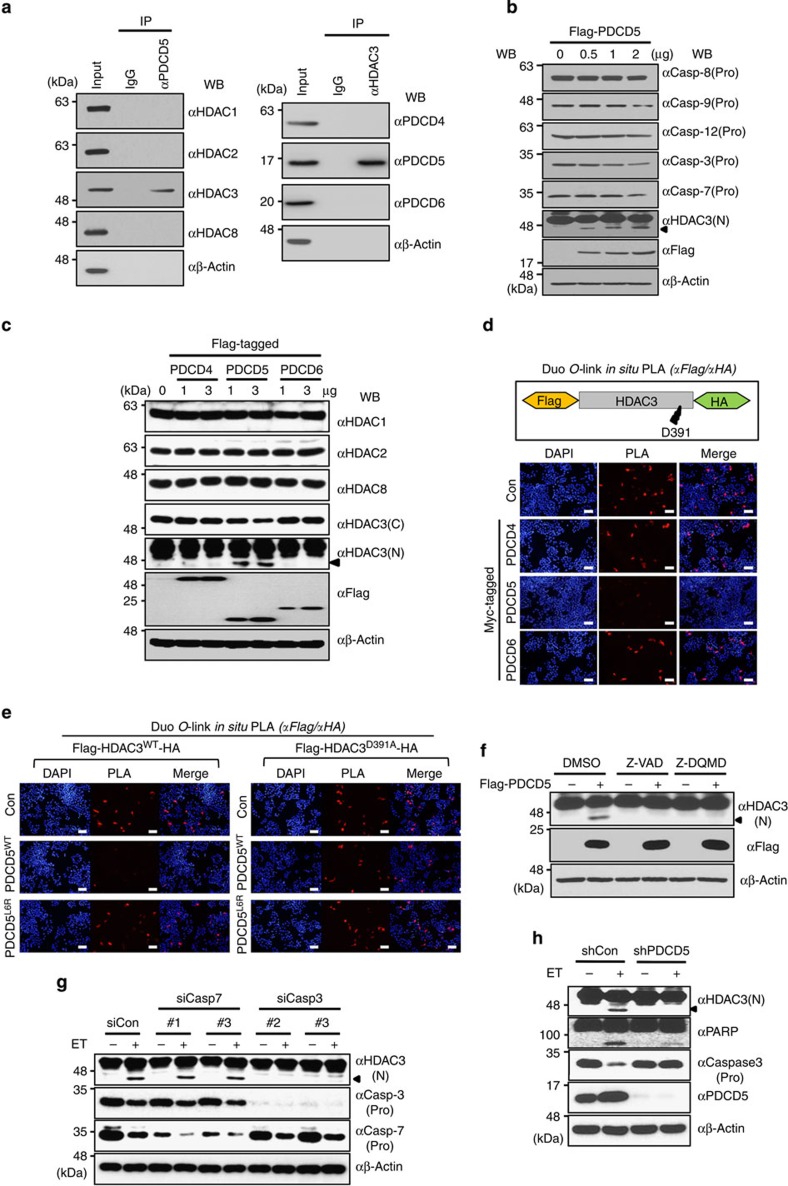
PDCD5 selectively binds to and mediates caspase-3-dependent cleavage of HDAC3 at Asp-391. (**a**) PDCD5 is an HDAC3-associating protein among PDCD proteins. Proteins from HCT-116 (*p53*^*+/+*^) whole-cell lysate were immunoprecipitated and subsequently immunoblotted with the indicated antibodies. (**b**) Overexpression of PDCD5 induces C-terminal cleavage of HDAC3. Cells were transfected with increasing amounts of Flag-PDCD5 plasmid. Whole-cell lysates were immunoblotted with the indicated antibodies. Arrow indicates cleaved HDAC3. (**c**) Overexpression of PDCD5 selectively triggers the cleavage of HDAC3, but not other class I HDACs. Cells were transfected with increasing amounts of PDCD plasmids. Whole-cell lysates were immunoblotted with the indicated antibodies. Arrow indicates cleaved HDAC3. (**d**,**e**) *In vivo* validation of PDCD5-mediated HDAC3 cleavage at Asp-391. HCT-116 cells were transfected with the indicated plasmids. Permeabilized cells were incubated with antibodies against HA and Flag, and then PLA probes were added. Positive signals were analysed using confocal microscopy. Red dots display uncleaved HDAC3. Representative images of three independent experiments are shown. (**f**) Inhibition of caspase-3 abrogates PDCD5-induced HDAC3 cleavage. HCT-116 cells were transfected with Flag-PDCD5 plasmid and treated with the indicated caspase inhibitors. (**g**) Depletion of caspase-3 abrogates ET-induced HDAC3 cleavage. Cells were transfected with siRNAs as indicated and treated with ET (100 μM, 12 h). Whole-cell lysates were immunoblotted with the indicated antibodies. (**h**) PDCD5 is required for caspase-3-dependent HDAC3 cleavage during ET treatment. Either shcontrol or stable shPDCD5-expressing HCT-116 cells was treated with ET. Whole-cell lysates were analysed by western blotting with the indicated antibodies. Scale bar, 10 μm.

To better understand the functional role of HDAC3 in apoptotic signalling, we performed the yeast two-hybrid screen using human testis, ovary and breast tissue libraries and identified 23 HDAC3-interacting proteins (Supplementary Table 1). Among the identified proteins, PDCD5, a tumour suppressor that was recently found to act as a pro-apoptotic factor, was particularly interesting and thus selected for this study. Moreover, we detected HDAC3 in an immunopurified PDCD5 complex with a high confidence using liquid chromatography–mass spectrometry (MS)/MS analysis, suggesting an *in vivo* interaction between HDAC3 and PDCD5 (Supplementary Table 2). Endogenous co-immunoprecipitation analysis demonstrated that PDCD5 selectively interacts with HDAC3 among class I HDACs. Reciprocally, HDAC3 specifically interacted with PDCD5 among PDCD proteins, which are known to be involved in apoptotic signalling. These results indicate that HDAC3, among class I HDACs, is a unique PDCD5-associating protein (Fig. 1a). Same results were also obtained with A2780 ovarian cancer cells (Supplementary Fig. 1a,b). Mapping analysis showed that the N-terminal domain of PDCD5 (1–30 amino acids (a.a.)) directly interacts with the N-terminal domain (1–106 a.a.) of HDAC3 (Supplementary Fig. 1c,d).

The C-terminal cleavage of HDAC3 at Asp-391 was reported to be important for cellular stress-induced apoptosis^11^,^12^. Moreover, PDCD5 has been shown to induce apoptotic cell death and upregulate expression of genes involved in apoptosis^20^,^21^. Thus, we first explored the possibility of PDCD5 involvement in C-terminal cleavage of HDAC3. Importantly, overexpression of PDCD5, but not PDCD4 or PDCD6, selectively induced cleavage of HDAC3, among class I HDACs, indicating the exclusive role of PDCD5 in the C-terminal cleavage of HDAC3 (Fig. 1b,c). To verify *in vivo* cleavage of HDAC3 by PDCD5, a proximity ligation assay (PLA) was performed^25^ using a double-tagged HDAC3 construct (Flag-HDAC3-HA). Overexpression of PDCD5, but not PDCD4 or PDCD6, efficiently induced the cleavage of HDAC3 (Fig. 1d). By contrast, a point mutant with defective interaction with HDAC3, PDCD5^L6R^, failed to induce HDAC3 cleavage, suggesting that interaction with PDCD5 is required for HDAC3 cleavage (Fig. 1e, left panel and Supplementary Fig. 2). Because Asp-391 of HDAC3 is known to be a conserved cleavage site, we next investigated whether this site is a target for PDCD5-mediated cleavage. As expected, PDCD5 induced the cleavage of wild-type but not mutant HDAC3^D391A^ (Fig. 1e, right panel).

As caspase-7 has been reported to be an executioner for HDAC3 cleavage during osmotic stress^11^, we next examined whether caspase-7 is also required for PDCD5-mediated HDAC3 cleavage. As expected, treatment with the pan-caspase inhibitor, Z-VAD, abrogated PDCD5-mediated HDAC3 cleavage. Unexpectedly, treatment with the caspase-3-specific inhibitor, Z-DQMD, unexpectedly blocked PDCD5-mediated HDAC3 cleavage (Fig. 1f). Furthermore, inhibition of caspase-7 had no effects on the HDAC3 cleavage by ET treatment (Fig. 1g; Supplementary Fig. 3). ET treatment efficiently induced HDAC3 cleavage in HCT-116 (p53^+/+^) cells; however, PDCD5 knockdown inhibited ET-induced HDAC3 cleavage, suggesting PDCD5 mediates ET-induced HDAC3 cleavage (Fig. 1h). These collectively demonstrated that PDCD5 induces caspase-3-mediated HDAC3 cleavage in response to ET.

### PDCD5 stabilizes p53 by inhibiting HDAC3–p53 interactions

**Figure 2:**
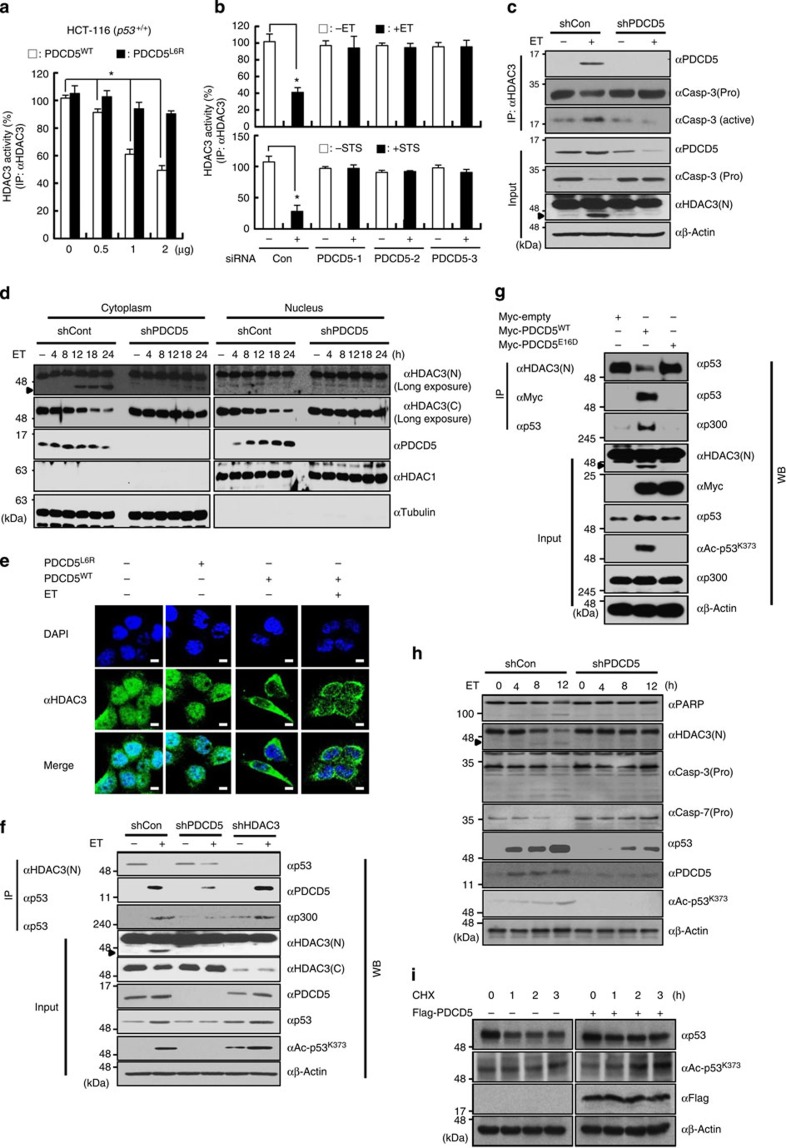
PDCD5 stabilizes p53 by inducing dissociation of the HDAC3–p53 complex and concomitantly triggering cytosolic cleavage of HDAC3. (**a**) Overexpression of PDCD5 reduces the activity of HDAC3. HCT-116 cells were transfected with the indicated plasmids. Whole-cell lysates were immunoprecipitated with anti-HDAC3 antibody, and then HDAC3 activity was measured. Error bars, s.d. (*n*=3). *P<0.05. (**b**) Depletion of PDCD5 abolishes the ET-induced reduction of HDAC3 activity. Cells were transfected with the indicated siRNAs. Cells were treated with ET (100 μM, 12 h) or STS (1 μM, 12 h) and assayed for HDAC3 activity. Error bars, s.d. (*n*=3). *P<0.05. (**c**) Knockdown of PDCD5 abrogates the association of HDAC3 with active caspase-3 in response to ET treatment. Whole-cell lysates were immunoprecipitated with anti-HDAC3 antibody, and subsequently immunoblotted with the indicated antibodies. Arrow indicates cleaved HDAC3. (**d**) Knockdown of PDCD5 prevents cytoplasmic cleavage of HDAC3 in response to ET treatment. Following cell fractionation, fractions were immunoblotted with the indicated antibodies. (**e**) Overexpression of PDCD5 enhances ET-induced cytoplasmic translocation of HDAC3. Immunofluorescence analysis was performed as described in the Supplementary Experimental Procedures Section. Representative images of three independent experiments are shown. (**f**) PDCD5 knockdown diminishes the ET-induced dissociation of HDAC3 from p53. Indicated shRNA-expressing HCT-116 cells were treated with ET. Whole-cell lysates were analysed by western blotting with the indicated antibodies. (**g**) Overexpression of PDCD5 dissociates HDAC3 from p53. Cells were transfected with PDCD5 plasmids. Immunoprecipitation and immunoblotting analyses were performed with the indicated antibodies. (**h**,**i**) PDCD5 increases p53 acetylation and stability via mediating HDAC3 cleavage. Cells were treated with ET (**h**) or cycloheximide (**i**) and subsequently immunoblotted with the indicated antibodies. Scale bar, 10 μm.

Given results suggesting PDCD5 is involved in caspase-3-dependent HDAC3 cleavage, we next investigated the role of PDCD5 in regulation of HDAC3 function. Because HDAC activity is known to be a critical function of HDAC3 (ref. 26), we first assessed the change in HDAC3 activity after either overexpression or depletion of PDCD5 in cells. An increase in wild-type PDCD5 expression, but not mutant PDCD5^L6R^, resulted in a decrease of HDAC3 histone deacetylase activity (Fig. 2a). However, overexpression of either PDCD4 or PDCD6 had no effect on HDAC3 histone deacetylase activity (Supplementary Fig. 4). Conversely, PDCD5 knockdown inhibited ET-induced reduction of HDAC3 activity, indicating that PDCD5 selectively mediates ET-induced effects on HDAC3 (Fig. 2b).

Next, we examined the molecular basis of PDCD5 involvement in ET-induced HDAC3 cleavage and inhibition. ET treatment of cells markedly increased the interaction between HDAC3 and active caspase-3. However, knockdown of PDCD5 levels abrogated this enhanced association (Fig. 2c). Importantly, while ET treatment induced cytoplasmic accumulation of cleaved HDAC3, knockdown of PDCD5 abolished this ET-induced cleavage (Fig. 2d; Supplementary Fig. 5a). Conversely, ET treatment of cells overexpressing wild-type PDCD5 further enhanced the cytoplasmic accumulation of HDAC3 (Fig. 2e). These data collectively demonstrate that ET triggers nuclear translocation of PDCD5, which in turn induces nuclear export of HDAC3 and caspase-3-mediated HDAC3 cleavage.

Acetylation and stability of p53 is coordinated by histone acetytransferases and HDACs^27^. Among HDACs, HDAC3 was recently shown to interact with p53 through MAGE-A^9^. Thus, we hypothesized that nuclear PDCD5 induces dissociation of HDAC3 from p53, subsequently triggering the cytoplasmic translocation and caspase-3-dependent cleavage of HDAC3, in turn increasing the acetylation level of p53. Results from co-immunoprecipitation analyses showed ET treatment efficiently induced the removal of HDAC3 from p53 and concomitant association of p300 with p53. However, knockdown of PDCD5 abolished ET-induced HDAC3–p53 dissociation, and also decreased p53 acetylation and stability (Fig. 2f; Supplementary Fig. 5b). Intriguingly, we found that both HDAC3 and PDCD5 bind to the same region of p53 (100–170 a.a.) (Supplementary Fig. 6). An *in vitro* pull-down and deacetylation assay showed that HDAC3 directly binds to and deacetylates p53 (Supplementary Fig. 7a,b). An *in vitro* competition assay demonstrated that wild-type PDCD5, but not the point mutant PDCD5^E16D^ that has defective interaction with p53, competitively inhibited HDAC3 binding to p53 (Supplementary Figs 2 and 7c). Consistently, PDCD5^E16D^ overexpression did not affect HDAC3–p53 interaction or HDAC3 cleavage compared with that of PDCD5^WT^ (Fig. 2g). Unexpectedly, MAGE-A2 knockdown in both H1299 and HCT-116 (p53^−/−^) cells had negligible effect on the interaction between HDAC3 and p53 and the inhibition of p53 acetylation and stability by HDAC3 overexpression (Supplementary Fig. 8a). Given the negative role of MDM2 in PDCD5-induced p53 acetylation and activation^28^, we next examined the involvement of MDM2 in the PDCD5–HDAC3–p53 pathway using p53/MDM2 double-knockout mouse embryonic fibroblasts (MEFs). PDCD5^WT^, but not mutant PDCD5^E16D^, induced p53 acetylation and stabilization in p53/MDM2 double-knockout MEFs (Supplementary Fig. 8b). Overexpression of HDAC3^WT^, but not the inactive HDAC3^Y298F^ mutant, efficiently reduced p53 acetylation independently of MDM2 (Supplementary Fig. 8c). Collectively, these results suggest that the PDCD5–HDAC3 network regulates p53 stabilization independently of MDM2 and MAGE-A2. Both cycloheximide treatment and ET time-course experiments verified that PDCD5 mediates HDAC3 degradation and p53 acetylation in response to genotoxic stress (Fig. 2h,i). Collectively, these results suggest that PDCD5 stabilizes p53 by inhibiting HDAC3–p53 interactions, which leads to translocation of HDAC3 from the nucleus to the cytosol and subsequent caspase-3-dependent cleavage.

### PDCD5 induces cleavage and degradation of HDAC3

**Figure 3:**
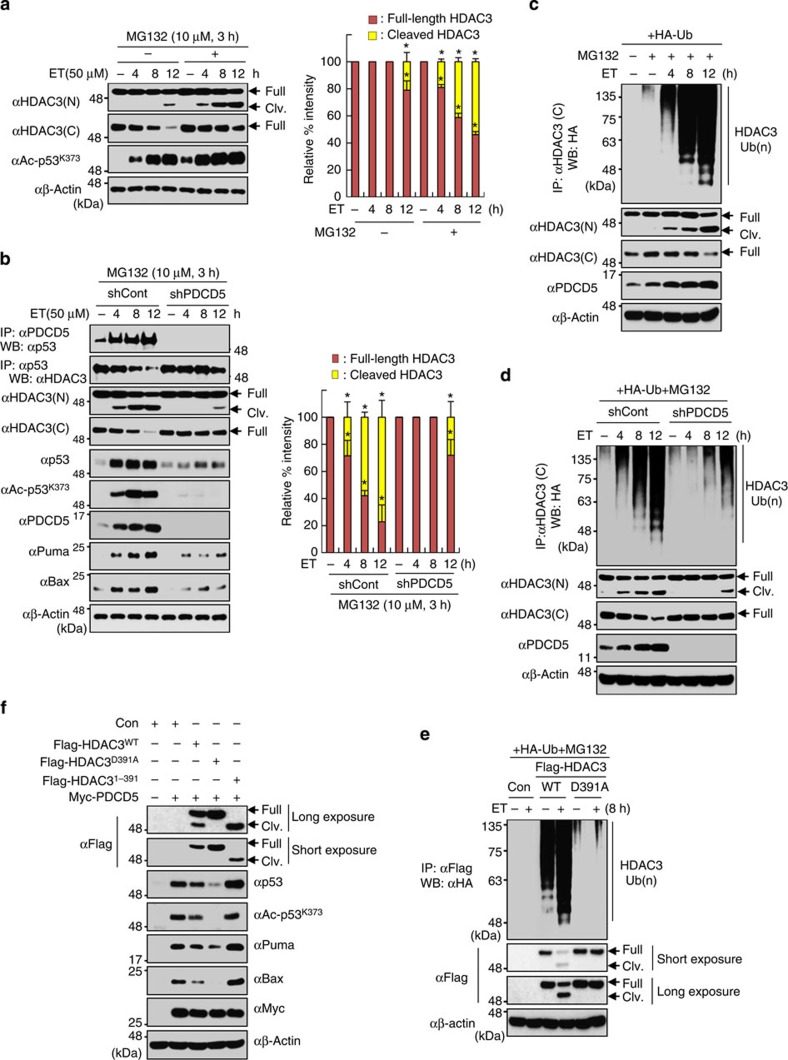
PDCD5 mediates ubiquitin-dependent proteasomal degradation of HDAC3 via C-terminal cleavage of HDAC3. (**a**) MG132 treatment induces accumulation of cleaved HDAC3. Cells were treated with ET (50 μM) and/or MG132 (10 μM, 3 h). Whole-cell lysates were immunoblotted with the indicated antibodies. Arrow indicates cleaved HDAC3 (left panel). Intensities of protein bands obtained from the immunoblotting assay were quantified with ImageJ (right panel) and normalized with respect to that of β-actin. Relative % intensity was calculated by dividing the normalized intensity by the sum of intensities from both cleaved and full-length HDAC3. Error bars, s.d. (*n*=3). (*P<0.01 versus without ET.) (**b**) PDCD5 knockdown diminishes the reduction and cleavage of full-length HDAC3. shcontrol or stable shPDCD5-expressing HCT-116 cells were treated with ET and/or MG132. Whole-cell lysates were immunoprecipitated and immunoblotted with the indicated antibodies (left panel). Relative % intensity was calculated as described above (right panel). Error bars, s.d. (*n*=3). (*P<0.01 versus without ET.) (**c**) HDAC3 ubiquitination increases in a time-dependent manner in response to ET treatment. Cells were transfected with HA-Ub plasmid and treated with MG1432 and/or ET. Whole-cell lysates were immunoprecipitated with anti-HDAC3 (C) antibody and immunoblotted with the indicated antibodies. (**d**) PDCD5 knockdown diminishes HDAC3 ubiquitination in response to ET. Whole-cell lysates were immunoprecipitated with anti-HDAC3 (C) antibody and immunoblotted with the indicated antibodies. (**e**) Mutation of Asp-391 abolishes ET-induced HDAC3 ubiquitination. Cells were transfected with HA-Ub and the indicated Flag-tagged plasmids, and treated with ET and/or MG132. Whole-cell lysates were immunoprecipitated with anti-Flag antibody and immunoblotted with the indicated antibodies. (**f**) Mutation of Asp-391 potentiates the action of HDAC3 in the inhibition of PDCD5-mediated p53 acetylation. Cells were transfected with the indicated Flag-tagged plasmids. Whole-cell lysates were immunoblotted with the indicated antibodies.

HDAC3 cleavage by PDCD5 correlates with increased p53 acetylation; however, HDAC3 cleavage is not observed during ET treatment at the time (4 h) when p53 is activated. We next examined whether the cleaved form of HDAC3 was rapidly degraded during ET treatment. At 2 h after the start of ET treatment, p53 acetylation at multiple lysine residues increased when the cleavage of HDAC3 was observed in the presence of MG132 (Supplementary Fig. 9a). At 4 h after the start of ET treatment, ∼20% of HDAC3 was observed as the cleaved form in the presence of MG132. At 12 h after the start of ET treatment, we found that ∼60% of full-length HDAC3 in the cell lysate was cleaved and dissociated from p53, which is consistent with maximum p53 activation (Fig. 3a). However, PDCD5 knockdown strongly reduced the percentage of HDAC3 cleavage and the dissociation of HDAC3 from p53 in response to ET treatment (Fig. 3b; Supplementary Fig. 9a). Similar results were observed by PDCD5 overexpression in the presence of MG132 (Supplementary Fig. 9b). We found that ET treatment induced the time-dependent ubiquitination and cleavage of HDAC3 (Fig. 3c); however, depletion of PDCD5 substantially reduced HDAC3 ubiquitination (Fig. 3d). By contrast, uncleaved mutant HDAC3^D391A^ was not ubiquitinated in response to ET treatment (Fig. 3e). Confocal microscopy analysis showed the nuclear retention of uncleaved HDAC3^D391A^ even after ET treatment, suggesting that cytoplasmic translocation and cleavage of HDAC3 is required for HDAC3 ubiquitination (Supplementary Fig. 9c). Moreover, HDAC3^D391A^ overexpression conferred stronger inhibition of PDCD5-induced p53 acetylation and activation that was conferred by HDAC3^WT^ (Fig. 3f). These results indicate that PDCD5 induces caspase-3-dependent HDAC3 cleavage, which leads to ubiquitin-dependent proteasomal degradation of HDAC3.

### CK2α-mediated phosphorylation enhances PDCD5 stabilization

**Figure 4:**
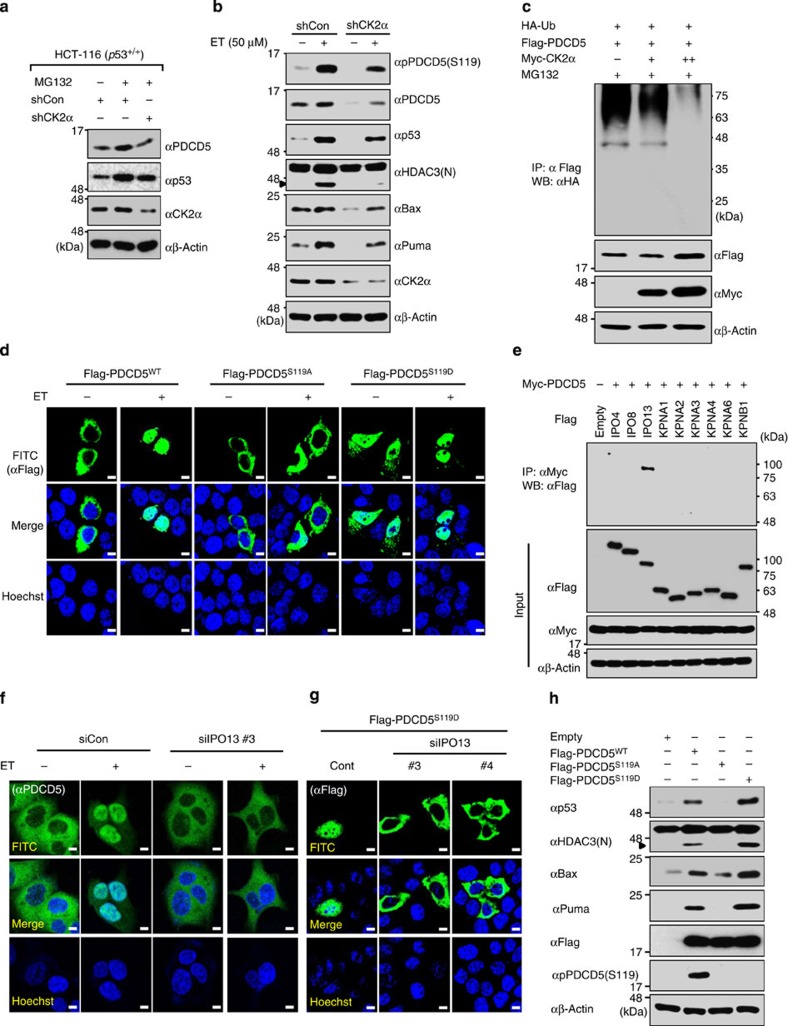
Casein kinase 2α enhances the stability and IPO13-mediated nuclear translocation of PDCD5 during genotoxic stress responses. (**a**) CK2α knockdown diminishes the effect of MG132 on PDCD5 stability. HCT-116 (p53^+/+^) cells were transfected with MG132 or shCK2α and immunoblotted with the indicated antibodies. (**b**) CK2α knockdown abolishes ET-induced HDAC3 cleavage, PDCD5 induction and PDCD5 phosphorylation. Either shcontrol or stable shCK2α-expressing HCT-116 cells were treated with ET. Whole-cell lysates were immunoblotted with the indicated antibodies. (**c**) CK2α overexpression inhibits PDCD5 ubiquitination. Cells were transfected with HA-Ub and the indicated Flag-tagged plasmids, and treated with MG132. Whole-cell lysates were immunoprecipitated with anti-Flag antibody and immunoblotted with the indicated antibodies. (**d**) CK2-mediated phosphorylation is required for ET-induced PDCD5 nuclear translocation. Cells were transfected with the indicated Flag-PDCD5 plasmids and treated with ET. Immunofluorescence analysis was performed as described in the Supplementary Experimental Procedures Section. (**e**) IPO13 selectively interacts with PDCD5 upon genotoxic stress response. Cells were transfected with HA-PDCD5 and the indicated Flag-tagged plasmids, and treated with ET. Whole-cell lysates were immunoprecipitated and immunoblotted with the indicated antibodies. (**f**) Knockdown of IPO13 abrogated the nuclear translocation of endogenous PDCD5 in response to ET. HCT-116 cells were transfected with the indicated siRNAs and treated with ET. Immunofluorescence analysis was performed as described in the Supplementary Experimental Procedures Section. (**g**) IPO13 knockdown abolishes the nuclear translocation of phosphor-PDCD5. HCT-116 cells were transfected with the indicated siRNAs and Flag-PDCD5 plasmid, and treated with ET. Immunofluorescence analysis was performed as described in the Supplementary Experimental Procedures Section. (**h**) Phospho-mimetic PDCD5^S119D^ mutant further promotes p53 acetylation and stabilization. Cells were transfected with the indicated Flag-PDCD5 plasmids. Whole-cell lysates were immunoblotted with the indicated antibodies. Scale bar, 10 μm.

We next examined how PDCD5 levels are increased and how PDCD5 is translocated into the nucleus in response to ET treatment. CK2α phosphorylated PDCD5 at Ser-119; mutation of this site disrupted the pro-apoptotic function of PDCD5 (ref. 29). Therefore, we tested whether CK2-mediated phosphorylation increased PDCD5 stability. Treatment with MG132 significantly increased PDCD5 levels, whereas CK2α knockdown diminished the effect of MG132 on PDCD5 stabilization (Fig. 4a). A cycloheximide time-course experiment showed that CK2α enhanced PDCD5 stability. CK2α knockdown reduced PDCD5 stability, indicating a crucial role for CK2α in PDCD5 stabilization (Supplementary Fig. 10a). To confirm these results, we generated a PDCD5 antibody that specifically recognized the phosphorylated Ser-119 residue. The phospho-specific antibody detected phosphorylated Ser-119 in PDCD5^WT^, but not in the PDCD5^S119A^ mutant, verifying its specificity (Supplementary Fig. 10b). We observed that the levels of both PDCD5 and phosphorylated PDCD5 concurrently increase in response to ET treatment. However, CK2 knockdown diminished the levels of PDCD5 and phosphorylated PDCD5, and reduced HDAC3 cleavage and p53 activation in response to ET (Fig. 4b; Supplementary Fig. 10c). CK2 overexpression markedly reduced PDCD5 ubiquitination (Fig. 4c) and increased PDCD5 stability, but not PDCD5 mRNA levels (Supplementary Fig. 10d). These results indicate that CK2-mediated phosphorylation of Ser-119 is required for PDCD5 stabilization.

### IPO13 mediates nuclear translocation of phosphor-PDCD5

We examined how PDCD5 is translocated into the nucleus in response to ET treatment. First, we performed bioinformatics analysis to search for nuclear localization signals (NLSs) in PDCD5, and identified a candidate NLS sequence in the N-terminal (1−20 a.a.) region. Then, we used site-directed mutagenesis to generate several different PDCD5 point mutants. Each PDCD5 point mutant was transfected into HCT-116 cells, and nuclear translocation was examined by confocal microscopy. Mutation of the candidate NLS sequence had no effect on PDCD5 subcellular localization in response to ET treatment (Supplementary Fig. 11a). However, mutation of the Ser-119 residue blocked PDCD5 translocation into the nucleus that was observed with wild-type PDCD5. Strikingly, the phospho-mimetic PDCD5 mutant (PDCD5^S119D^) localized primarily in the nucleus even without ET treatment, suggesting that CK2-mediated phosphorylation of Ser-119 is required for nuclear translocation of PDCD5 in response to genotoxic stress (Fig. 4d). Cell fractionation analysis again verified the above finding that phosphor-PDCD5 rapidly moved into nucleus upon ET treatment. However, knockdown of CK2α blocked the nuclear localization of PDCD5 (Supplementary Fig. 11b,c). There are evidences that phosphorylation activates non-canonical transport signals and the phosphorylated motif is recognized by nuclear receptor importin-β family without intervention of an importin α-like protein^30^. Strikingly, we found that PDCD5 selectively binds to a nuclear transport protein IPO13 in response to ET, and the knockdown of IPO13 abrogated the nuclear translocation of endogenous PDCD5 (Fig. 4e,f; Supplementary Fig. 11d). Importantly, knockdown of IPO13 abolished the nuclear retention of phospho-mimetic PDCD5^S119D^ mutant (Fig. 4g). Moreover, IPO13 strongly binds to phosphor-mimetic PDCD5^S119D^ but not with the phospho-mutant PDCD5^S119A^, indicating that IPO13 is indispensable for nuclear translocation of phosphor-PDCD5 (Supplementary Fig. 11f). Finally, overexpression of PDCD5^S119D^ induced HDAC3 cleavage and p53 activation more strongly than that induced by wild-type PDCD5. By contrast, the phospho-mutant PDCD5^S119A^ had no effect on HDAC3 cleavage and p53 activation (Fig. 4h). Collectively, these results demonstrate that CK2-dependent phosphorylation is required for IPO13-mediated nuclear translocation of PDCD5, which leads to HDAC3 cleavage in response to ET treatment.

### PDCD5 promotes p53 function via HDAC3 inhibition

**Figure 5:**
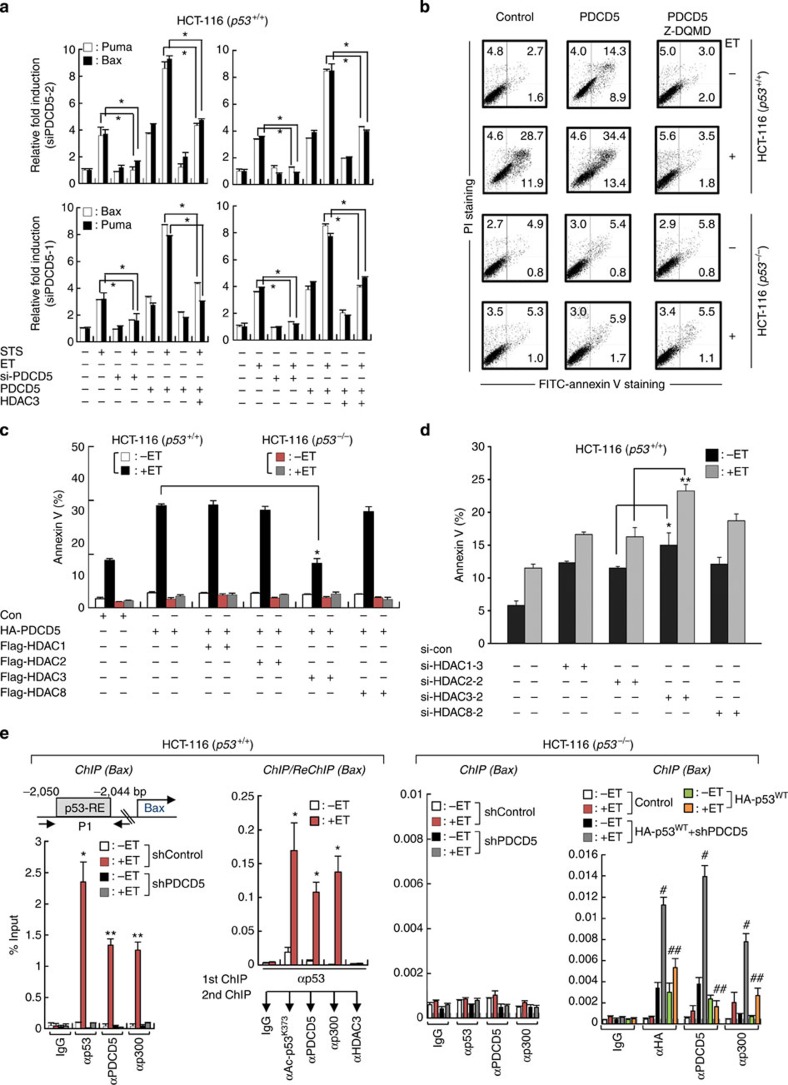
PDCD5 promotes p53-dependent apoptosis via selective inhibition of HDAC3. (**a**) Overexpression of HDAC3 suppresses the PDCD5-mediated transcription of p53-target genes. Cells were transfected with indicated siRNAs and/or plasmids and treated with either ET or STS. The levels of indicated genes were analysed by real-time PCR. Error bars, s.d. (*n*=3). *P<0.05. (**b**) PDCD5 promotes p53-dependent apoptosis in a caspase-3-dependent manner. Cells were transfected with indicated plasmids and treated with ET and/or Z-DQMD. Annexin V-positive cells were assessed by flow cytometry. A representative figure of three independent experiments is shown. (**c**) HDAC3, but not other class I HDACs tested, selectively antagonizes PDCD5-enhanced apoptosis. Annexin V-positive cells were assessed by flow cytometry. Error bars, s.d. (*n*=3). *P<0.05. (**d**) Knockdown of HDAC3 significantly enhances ET-induced apoptosis. Annexin V-positive cells were assessed by flow cytometry. Error bars, s.d. (*n*=3). *P<0.05; **P<0.01. (**e**) PDCD5 is required for ET-induced recruitment of the p53–p300 complex to the promoter region of *Bax*. Cells were transfected with indicated plasmids and/or shPDCD5, and then treated with ET. ChIP and re-ChIP assays were performed with the indicated antibodies. Precipitated samples were analysed by real-time PCR, and results are presented as the percentage of input. Error bars, s.d. (*n*=3). *P<0.05, **P<0.01 versus without ET; ^#^*P*<0.05 versus without ET; ^##^*P*<0.05 versus ET+HA-p53^WT^.

To investigate the functional significance of PDCD5-mediated HDAC3 cleavage on p53-dependent apoptosis, we observed the modulation of pro-apoptotic genes after knocking down or overexpressing PDCD5 and/or HDAC3. PDCD5 knockdown abrogated both ET- and staurosporine (STS)-induced transcription of p53-target genes, Puma and Bax. Accordingly, overexpression of PDCD5 further enhanced ET-induced p53-target gene expression. Strikingly, coexpression of both PDCD5 and HDAC3 attenuated the positive action of PDCD5 on ET-induced p53-target gene expression, indicating an antagonistic function of HDAC3 in PDCD5-mediated apoptosis (Fig. 5a).

Next, we examined whether the pro-apoptotic function of PDCD5 is dependent on p53. Overexpression of PDCD5 in HCT-116 (*p53*^*+/+*^) cells yielded a significant increase in annexin V-positive cells compared with HCT-116 (*p53*^−/−^). Moreover, both inhibition and knockdown of caspase-3 fully abrogated PDCD5-enhanced apoptotic cell death in HCT-116 (*p53*^*+/+*^) cells, but not in HCT-116 (*p53*^−/−^), suggesting that PDCD5 promotes genotoxic stress-induced apoptosis in a p53-dependent manner (Fig. 5b; Supplementary Fig. 12a). Strikingly, overexpression of HDAC3 selectively antagonized PDCD5-mediated apoptosis, indicating the exclusive role of HDAC3 in PDCD5-mediated apoptotic signalling (Fig. 5c; Supplementary Fig. 12b). Although depletion of class I HDACs commonly enhanced ET-induced apoptosis, likely due to their activity in genome maintenance, depletion of HDAC3 further enhanced ET-induced apoptosis of HCT-116 (*p53*^*+/+*^) cells than that induced by other HDACs (Fig. 5d). Collectively, these data indicate that PDCD5 negatively regulates anti-apoptotic action of HDAC3 in a p53-dependent manner.

p53 mediates transcriptional activation upon binding to the promoter region of its target genes. Intriguingly, structure analysis of PDCD5 protein using Conserved Domain Database showed that PDCD5 possesses a double-strand DNA-binding domain^31^. Given our findings that show enhanced interaction between PDCD5 and p53 in response to ET, we next explored the possibility that PDCD5 forms a complex with p53 that enhances recruitment to the promoter region of target genes. For this, we performed chromatin immunoprecipitation (ChIP) analyses against the p53-binding site (p53-RE) on *Bax*. ChIP assays showed that ET efficiently induced the binding of p53 and p300 to p53-RE of *Bax* (Fig. 5e, first panel). ChIP and re-ChIP analyses further verified the presence of an acetylated p53–PDCD5–p300 complex in the p53-RE region of *Bax* (Fig. 5e, second panel). Strikingly, the depletion of PDCD5 abrogated ET-induced recruitment of the p53–p300 complex to the p53-binding site of *Bax* (Fig. 5e, first panel). In addition, the restoration of p53 in HCT-116 (p53^−/−^) cells efficiently regained the effect of ET on recruitment of PDCD5 and p300 to *Bax*. Knockdown of PDCD5, however, greatly abolished binding of the p53–p300 complex to *Bax* (Fig. 5e, fourth panel). Similar results were observed in the p53-RE on the *Puma* gene (Supplementary Fig. 13a). Collectively, these data demonstrate that PDCD5 mediates the removal of HDAC3 from p53 upon genotoxic stress, and subsequently forms a complex with p53 and p300 that promotes transcriptional activation of p53-target genes.

### PDCD5 mediates acetylation-dependent p53 stabilization

**Figure 6:**
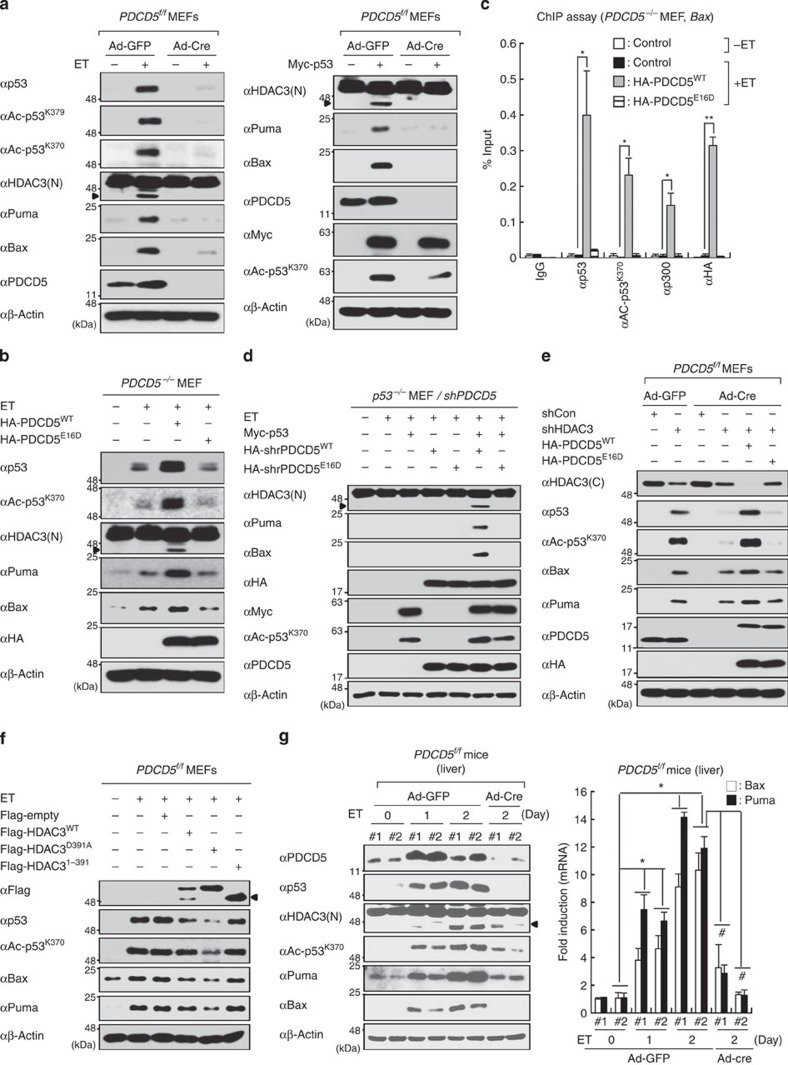
PDCD5 is required for genotoxic stress-induced HDAC3 cleavage and p53 activation. (**a**) Depletion of PDCD5 abrogated the ET-induced HDAC3 cleavage and p53 activation. MEFs were infected with Ad-Cre or Ad-GFP and then electroporated with Myc-p53 or treated with ET (50 μM, 8 h). Whole-cell lysates were immunoblotted with indicated antibodies. Arrow indicates cleaved HDAC3. (**b**) PDCD5 promotes ET-induced p53 activation and HDAC3 cleavage. *PDCD5*^−/−^ MEFs were electroporated with indicated plasmids, treated with ET, lysed and then analysed by immunoblotting. (**c**) Restoration of PDCD5 into PDCD5^−/−^ MEFs induces the recruitment of the p53–p300 complex to the promoter region of *Bax*. ChIP assays were performed with the indicated antibodies. Error bars, s.d. (*n*=3). *P<0.05, **P<0.01. (**d**) Both PDCD5 and p53 are mutually required for ET-induced activation of apoptosis. Stable shPDCD5-expressing *p53*^−/−^ MEFs were electroporated with indicated plasmids and treated with ET. (**e**) Knockdown of HDAC3 rescues the suppression of p53 caused by depletion of PDCD5. *PDCD5*^−/−^ MEFs were electroporated with indicated plasmids and/or shHDAC3, and cell lysates were analysed by immunoblotting. (**f**) Negative effect of cleavage at Asp-391 on the anti-apoptotic function of HDAC3. Cell lysates were analysed by immunoblotting. (**g**) Ablation of PDCD5 abolishes the genotoxic stress response *in vivo*. Mice were injected with ET (10 mg kg^−1^) for the indicated days. Adenovirus expressing GFP or Cre recombinase was injected in mice 6 days before ET injection, as indicated. Tissues from individual livers were harvested and processed for western blotting. Total RNA was isolated from individual livers, and qRT–PCR was performed for the indicated genes. Error bars, s.d. (*n*=8). *P<0.05 versus without ET; ^#^*P*<0.05 versus ET (2 days).

Our results illustrating the p53-dependent action of PDCD5 in genotoxic stress responses led us to further investigate whether PDCD5 is critical for the stabilization and activation of p53. For this, we generated *PDCD5*^*flox/flox*^ mice (Supplementary Fig. 14). MEFs generated from these mice were subjected to a Cre recombinase (Ad-Cre)-expressing adenovirus to delete PDCD5, generating a *PDCD5*^−/−^ MEF line. Ad-Cre recombinase treatment depleted most PDCD5 protein in MEFs derived from *PDCD5*^*f/f*^ mice (Fig. 6a). Depletion of PDCD5 strongly reduced HDAC3 cleavage and p53 activation in response to ET treatment and p53 overexpression (Fig. 6a; Supplementary Fig. 15a). Consistently, a Mdm2 inhibitor, nutlin3a treatments had negligible effects on HDAC3 cleavage and p53 activation in *PDCD5*^−/−^ MEFs (Supplementary Fig. 15b). However, rescue of *PDCD5*^−/−^ MEFs with haemagglutinin (HA)-tagged PDCD5^WT^, but not with PDCD5^E16D^, markedly increased p53 activity, HDAC3 cleavage and recruitment of the p53–p300 complex to the p53-RE of *Bax* and *Puma*, indicating that PDCD5 is required for p53 activation via HDAC3 cleavage (Fig. 6b,c; Supplementary Figs 13b and 15b,c). Importantly, rescue of PDCD5-knockdown *p53*^−/−^ MEFs with p53 and wild-type PDCD5^WT^, but not mutant PDCD5^E16D^, resulted in regained p53 action on ET-induced HDAC3 cleavage, target gene expression and apoptosis (Fig. 6d; Supplementary Fig. 15d). These results again verify that PDCD5 and p53 are mutually required for genotoxic stress-induced apoptosis and HDAC3 cleavage. We observed that HDAC3 knockdown rescued p53 suppression caused by PDCD5 depletion (Fig. 6e; lane 3 versus 4). Restoration of PDCD5^WT^, but not PDCD5^E16D^, further enhanced the effect of HDAC3 knockdown on p53 acetylation and activation (lane 2 versus 5), again confirming the crucial role of PDCD5 in acetylation-induced p53 stabilization (Fig. 6e; Supplementary Fig. 15e). Notably, overexpression of uncleaved mutant HDAC3^D391A^ markedly suppressed ET-induced p53 acetylation and activation, as well as apoptosis, when compared with wild-type HDAC3^WT^. As expected, the cleaved form of HDAC3 (HDAC3^1–391^) had negligible effects on both p53 activation and apoptosis, supporting our notion that HDAC3 function is negatively regulated during genotoxic stress responses (Fig. 6f; Supplementary Figs 13c and 15f).

To further corroborate the critical role of PDCD5 in genotoxic stress-induced DNA damage *in vivo*, *PDCD5*^*f/f*^ mice were treated with ET followed by injection of adenovirus for green fluorescent protein (GFP) or Cre recombinase to ablate PDCD5 mRNA expression in liver. ET injection significantly induced the HDAC3 cleavage and levels of p53 at 1 and 2 days when compared without ET injection. However, depletion of PDCD5 markedly diminished HDAC3 cleavage and levels of p53. As expected, the mRNA levels of Puma and p21 were not increased in the depletion of PDCD5 (Fig. 6g). Collectively, these data demonstrate that PDCD5 plays an essential role in p53 activation by modulating the decay of HDAC3 *in vivo*.

### Reduction of PDCD5 and p53 enhances gastric tumorigenesis

**Figure 7:**
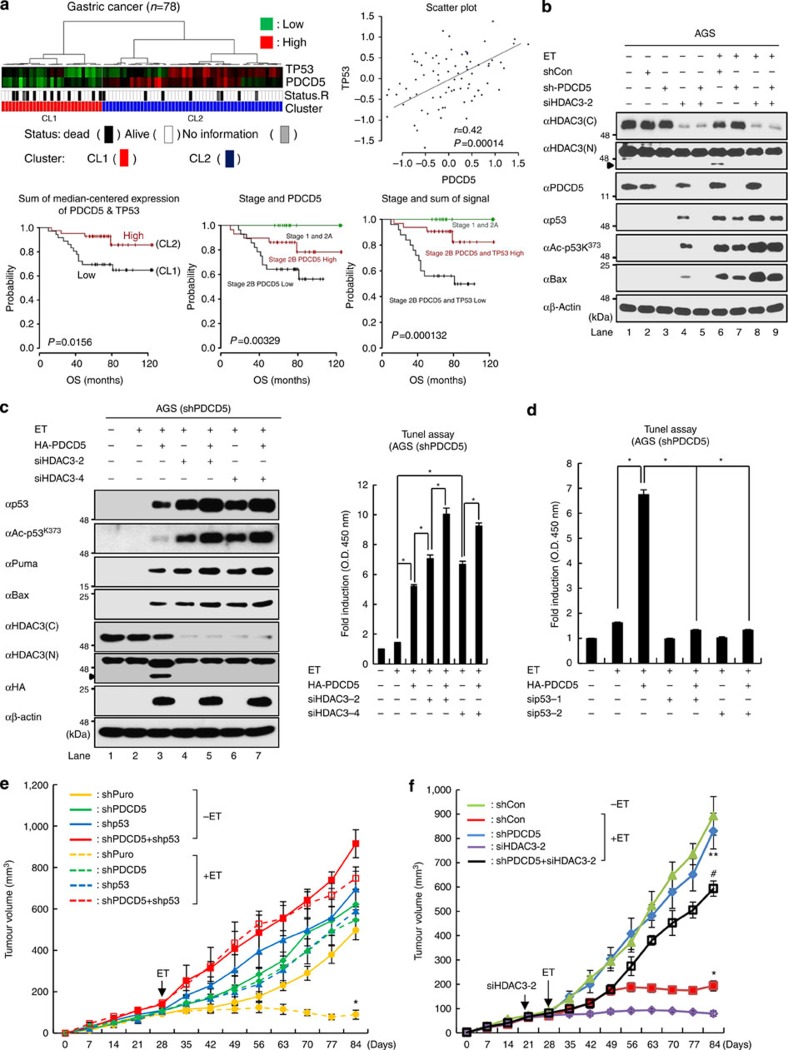
Reduction of both PDCD5 and p53 synergistically enhance *in vivo* tumorigenicity of gastric cancer cells. (**a**) Reduction of PDCD5 and p53 significantly correlates with poor survival in stage 2b gastric cancer patients. Kaplan–Meier plots and log-rank test were used to estimate the prognostic differences of categorized patient groups. (**b**) Depletion of PDCD5 diminishes the effect of HDAC3 knockdown on p53 acetylation and activation. AGS cells were transfected with siHDAC3 and/or shPDCD5 as indicated, and treated with or without ET (75 μM, 8 h). Whole-cell lysates were immunoblotted with indicated antibodies. (**c**) Restoration of PDCD5 with HDAC3 knockdown potentiates ET-induced p53 activation. Stable shPDCD5-expressing AGS cells were transfected with indicated plasmids and/or siHDAC3, and then treated with ET. DNA damage of cells was determined by the TUNEL assay. Error bars, s.d. (*n*=3). *P<0.01. (**d**) Reduction of PDCD5 and p53 synergistically reduces the genotoxic response of AGS cells. Stable shPDCD5-expressing AGS cells were transfected with indicated plasmids and/or siRNA, and then the cells were treated with ET. DNA damage of cells was determined by the TUNEL assay. Error bars, s.d. (*n*=3). *P<0.01. (**e**) Reduction of PDCD5 and p53 significantly reduces the chemosensitivity of AGS cells. Stable AGS cells were injected subcutaneously into the right flank of nude mice. Four weeks after injection, mice with comparable-sized tumours (100∼200 mm^3^) were selected for treatment with ET (10 mg kg^−1^), with 2-day intervals for 8 weeks. Tumour volumes were measured for 12 weeks. Error bars indicate s.d. (*n*=6). *P<0.05 versus without ET+shPuro. (**f**) Knockdown of HDAC3 reversed the impaired chemosensitivity of AGS cells by depletion of PDCD5. Stable shCon or shPDCD5 AGS cells were injected subcutaneously into the right flank of nude mice. Four weeks after injection, mice with comparable-sized tumours (100∼200 mm^3^) were selected for treatment with etoposide (10 mg kg^−1^), with 2-day intervals for 8 weeks. Detailed procedure for siHDAC3 treatment is described in the Methods. Tumour volumes were measured for 12 weeks. *P<0.05 versus shCon without ET; **P<0.05 versus shCon with ET; ^#^*P*<0.05 versus shPDCD5 with ET. Error bars indicate s.d. (*n*=6).

Reduced expression of PDCD5 has been reported in patients with multiple cancer types including gastric, lung, ovarian and glioma^16^,^17^,^18^,^32^. We investigated the pathological correlation between PDCD5 and p53 in Korean gastric cancer samples. Tumour tissues from 78 lymph-node-negative (N0-stage patients having no lymph node metastasis) gastric adenocarcinoma patients were used to generate gene expression profiles using Illumina human bead arrays (HumanHT-12, v3.0, Illumina, San Diego, CA). Pearson correlation coefficient calculations between PDCD5 and p53 in gastric cancer patients' gene expression profiles revealed a strong positive correlation (*r*=0.42; *P*=0.00014) (Fig. 7a, upper panel). When we investigated the prognostic power of PDCD5 and p53, neither PDCD5 nor p53 expression levels alone showed significant prognostic discrimination among gastric cancer. However, the combined signature of PDCD5 and p53 showed strong prognostic powers in N0-stage gastric cancer patients (log-rank test *P*=0.015) (Fig. 7a, lower left panel). As all of stage 1 and 2a patients survived, we next sub-stratified patients into three groups: stage 1 and 2a patients, stage 2b PDCD5 plus p53 (high) and stage 2b PDCD5 plus p53 (low). Patients showing increased expression of PDCD5 and p53 showed relatively good prognostic outcomes (over 80% 5-year survival rate), while the prognostic outcome of patients showing low expression of PDCD5 and p53 in stage 2b was more than 50% of death rates. We conclude that non-lymph node metastasis gastric cancer patients can be stratified based on disease stage and PDCD5 and p53 signature to show distinct prognostic differences (log-rank test *P*=0.000132) (Fig. 7a, lower middle/right panel).

We again verified PDCD5-dependent p53 action in response to genotoxic stress using the wild-type p53-positive gastric cancer cell, AGS. As consistently, ET treatment had negligible effects on HDAC3 cleavage and p53 activation in the absence of PDCD5. Moreover, knockdown of HDAC3 markedly increased p53 stability and activation, while depletion of PDCD5 abrogated this effect (Fig. 7b). Notably, knockdown of HDAC3 further potentiated the effects of PDCD5 restoration on ET-induced p53 activation and HDAC3 cleavage, supporting our notion that PDCD5 promotes p53 acetylation by suppressing HDAC3 function (Fig. 7c). Importantly, reduction of both PDCD5 and p53 levels diminished the effect of ET on DNA damage response more than knockdown of either protein individually (Fig. 7d). Finally, an *in vivo* tumorigenicity assay using subcutaneous injection of AGS cells into nude mice demonstrated that reduction of either PDCD5 or p53 significantly increased the tumour growth compared with control short hairpin RNA (shRNA) (Supplementary Fig. 16a–c). Strikingly, reduction of both PDCD5 and p53 further enhanced tumour growth and chemoresistance of AGS cells as compared with reduction of the respective protein (Fig. 7e; Supplementary Fig. 16d). Co-knockdown of PDCD5 and HDAC3 significantly increased the chemosensitivity of AGS cells compared with knockdown of PDCD5 alone, indicating a negative action of HDAC3 in p53-mediated genotoxic stress responses (Fig. 7f; Supplementary Fig. 16e,f). Collectively, these data demonstrate that reduced levels of PDCD5 and p53 enhance *in vivo* tumorigenic growth of AGS gastric cancer cells and correlate with poor survival in gastric cancer patients.

## Discussion

Here, we found that p53 function is reversibly regulated by both HDAC3 and PDCD5 during genotoxic stress response. There has been accumulating evidence that inhibition of HDAC3 enhances p53 acetylation and stability in human cancer and normal cell lines^7^,^8^,^33^. Furthermore, a recent report showed that HDAC3 suppresses p53 activity in coordination with MAGE-A, which confers resistance to chemotherapeutic agents^9^. However, we here observed direct interaction between HDAC3 and p53, and found that HDAC3 deacetylated p53 *in vitro*. These data suggest that HDAC3 inhibition directly affects p53 acetylation. Furthermore, MAGE-A knockdown had no effect on HDAC3–p53 interaction or HDAC3-mediated inhibition of p53 acetylation and stability. On the basis of these results, it is likely that HDAC3 regulates the acetylation of p53 independently of MAGE-A. We do not exclude the possibility that MAGE-A recruits HDAC3 to inhibit p53 function under certain cellular stresses or a certain type of cells. Further work is necessary to elucidate functional relationships between HDAC3 and MAGE-A in regulating p53 activity.

Although there is evidence for HDAC3 cleavage during apoptosis, a detailed understanding of the effects of C-terminal cleavage on HDAC3 function is lacking. In this study, we found that PDCD5-mediated HDAC3 cleavage leads to ubiquitin-dependent proteasomal degradation of HDAC3. PDCD5 knockdown strongly reduced HDAC3 ubiquitination in response to ET treatment. Overexpression of uncleaved HDAC3^D391A^ inhibited PDCD5-induced p53 acetylation and stabilization more effectively compared with that of wild-type and cleaved (1−391) HDAC3. We showed that PDCD5 competitively inhibited HDAC3–p53 interaction in response to ET treatment, whereas overexpression of the PDCD5^E16D^ mutant, which is defective in its interaction with p53, failed to induce HDAC3 cleavage and p53 activation. These results indicate that PDCD5 and HDAC3 competitively bind to p53 in response to ET treatment, which induces HDAC3 cleavage by caspase-3 and ultimately leads to degradation of the cleaved form of HDAC3.

Previous reports show that PDCD5 is rapidly upregulated and translocated into the nucleus during DNA damage responses; however, the underlying mechanism was unknown. We found that CK2 participated in the positive regulation of PDCD5 function during genotoxic stress responses. We generated phospho-specific PDCD5 antibody, which confirmed that CK2-mediated phosphorylation of PDCD5 at Ser-119 increased concurrently with increasing PDCD5 levels during ET treatment. We showed that CK2 depletion blocked ET-induced PDCD5 stabilization and phosphorylation, and CK2-mediated PDCD5 phosphorylation was required for nuclear translocation of PDCD5. The phospho-mimetic PDCD5 mutant was localized primarily in the nucleus regardless of ET treatment, and its overexpression more efficiently induced HDAC3 cleavage and p53 acetylation more effectively than that of wild-type PDCD5, indicating a positive action of CK2 in the pro-apoptotic function of PDCD5. Accumulating evidence demonstrates that CK2 enhances the stability or nuclear translocation of target proteins via phosphorylation of Ser/Thr residues^34^,^35^,^36^. Our results indicate that CK2 positively affects PDCD5 function by enhancing both stability and nuclear translocation. Although there is no obvious NLS in PDCD5, we found that phosphorylated PDCD5 is recognized and imported into the nucleus by importin IPO13, which is similar to ERK1/2 and ASF/SF2 (ref. 30). Importantly, knockdown of IPO13 abrogated the nuclear translocation of phospho-mimetic PDCD5^S119D^ mutant, highlighting that IPO13 mediates nuclear translocation of phosphor-PDCD5. Further work is necessary to unravel the details of PDCD5 nuclear transport.

On the contrary to previous findings^11^,^12^, we found that inhibition of caspase-3, but not caspase-7, completely blocked HDAC3 cleavage by ET and PDCD5 overexpression. These data suggest that caspase-3 is the principal executioner for PDCD5-mediated HDAC3 cleavage upon genotoxic stress. One possible scenario is that since caspase-3 is a major effector in human cancer cell lines, it is sufficient for proteolysis of the majority of substrates that are cleaved during the end of apoptosis. Furthermore, it has been shown that both caspase-3 and caspase-7 commonly cleave certain substrates such as PARP, but exhibit differential activity towards multiple substrates such as Bax, XIAP and cochaperone p23 (ref. 37). However, we do not exclude the possibility that caspase-7 may be capable of functionally substituting for caspase-3 in situations where both proteases are expressed at similar levels such as Jurkat and NIH3T3 cell lines.

Intriguingly, PDCD5 seems to possess a double-strand DNA-binding domain. This raises the possibility that PDCD5 binds to chromatin with p53 to activate transcription of pro-apoptotic genes. In here, we found that p53 is recruited to p53-RE of *Bax* and *Puma* after forming the p53–p300 complex in the presence of PDCD5. Moreover, rescue of *PDCD5*^−/−^ MEFs with PDCD5^WT^, but not mutant PDCD5^E16D^, markedly induced the formation of the p53–p300 complex in the promoter region of *Bax* and *Puma* genes. This finding suggests that PDCD5–p53 interaction is required for p53 binding to the target gene promoters, and this association promotes the transcription of pro-apoptotic genes (Supplementary Fig. 16g). Finally, our clinical analyses confirmed a positive correlation between PDCD5 and p53 and gastric cancer patient survival rates. Our data showed that survival rates of stage 2b gastric cancer patients with reduced levels of both PDCD5 and p53 are markedly decreased compared with those patients with reduction of PDCD5 alone. Furthermore, this result is well consistent with that from the mice xenograft assay. This suggests that reduction of PDCD5 and p53 levels may contribute to the early progression of human gastric cancer.

The use of HDAC inhibitors as anticancer treatments has been extensively studied, and several inhibitors are currently in clinical trials. Development of HDACi-based anticancer drugs, however, is limited due to unfavourable toxicities^38^. Though, recent studies in the development of selective HDAC inhibitors have demonstrated that HDAC3-selective inhibitors are promising candidates for anticancer drugs^39^,^40^. Thus, future directions for developing clinical applications for HDACi may focus on the use of HDAC subtype-specific inhibitors. A better understanding of individual HDAC function will pave the way for development of prospective HDAC inhibitors as anticancer therapeutics.

## Methods

### Immunoprecipitation and antibodies

The antibody against mouse PDCD5 was generated by LabFrontier (Anyang, South Korea) using the synthetic peptide TEKKTTVKFNRRKVMDSDEDDADY and used at a 1:500 dilution. The antibody against HDAC3 (N) and (C) were generated by LabFrontier using the synthetic peptide CLNVPLRDGIDDQSYKHLFQ and YDGDHDNDKESDVEI, respectively, and used at a 1:1,000 dilution. The antibody against phosphor-PDCD5^Ser119^ was generated by LabFrontier using the synthetic peptide ^111^FNRRKVMD*p*SDEDDDY^125^ (1:1,000). Antibodies against HDAC1 (sc-78972), HDAC2 (sc-7899), HDAC8 (sc-11405), pro-caspase-8 (sc-7272), pro-caspase-9 (sc-11405), p53 (sc-126 and sc-6243), caspase-3 (sc-7148), caspase-7 (SC-56063), p21 (sc-397), HA (sc805), MAGE-a2 (sc-130164), HDAC3(C-terminal) (sc-11417) and pro-caspase-12 (sc-70227) were purchased from Santa Cruz Biotechnology Inc. (Santa Cruz, CA, USA). Antibody against PDCD5 (12456-1-AP) was purchased from Proteintech Group Inc. (Chicago, IL, USA). FLAG (F3165) and β-actin (A5441) antibodies were obtained from Sigma-Aldrich. Myc (#2278S), acetyl-p53^K379^ (#2570), acetyl-p53^K382^ (#2525S) and active-caspase-3 (#9661S) antibodies were purchased from Cell Signaling (Beverly, MA, USA). Tubulin (05-829), GAPDH (CB1001) and p300 (N446) antibodies were obtained from Millipore (Billerica, MA, USA). PARP-1 (51-6639GR) antibody was purchased from BD Transduction Laboratories (Lexington, KY, USA). Bax (1063-1) and acetyl-p53^K373^ (#2204-1) antibodies were purchased from Epitomics Inc. PDCD4 (ab32831-100), PDCD6 (ab133326), IPO13 (ab101374), acetyl-p53^K381^ (ab61241) and Puma (ab133326) antibodies were purchased from Abcam (Cambridge, MA, USA). To quantify the intensity of immunoblotting, images were analysed using the ImageJ software (http://rsbweb.nih.gov/ij/). Briefly, to analyse the individual images for quantification, RGB colour images obtained from the immunoblotting analysis were converted to 8-bit grayscale images. Mean gray value and integrated density were quantified on each object in images according to the guide instruction of ImageJ. The results are shown as mean±s.d. calculated from three independent experiments. Working dilutions or quantities of the antibodies used in the study are summarized in Supplementary Table 3. Uncropped immunoblottings are provided as Supplementary Fig. 17.

### Yeast two-hybrid assay

HDAC3 bait plasmids (pGBKT7-HD3DO1 and pGBKT7-HD3DO2) were transformed into the yeast strain AH109. Transformants containing each bait plasmid were mated with the pre-transformed human Ovary, Breast and Testis MATCHMAKER cDNA library (Clontech, Shiga, Japan). Two-hybrid screening was performed according to the manufacturer's protocol. Plasmids were harvested from the positive clones that grew in minimal media lacking tryptophan, leucine, adenosine, histidine and β-galactosidase expression. Plasmids were identified by DNA sequencing.

### Mass spectrometry

The eluted immune complexes were precipitated with 20% trichloroacetic acid and the pellets were washed four times with cold acetone. The precipitated proteins were resuspended in 100 mM ammonium bicarbonate (pH 8.0) with 10% acetonitrile and incubated with sequencing-grade trypsin (Promega, Fitchburg, WI, USA) at a concentration of 12.5 ng ml^−1^ at 37 °C for 4 h. Trypsin reactions were quenched by addition of 5% formic acid, and peptides were desalted using the C_18_ Stage Tip method. For each liquid chromatography–MS/MS analysis, 4 μl of sample was loaded onto an EASY-Spray C_18_ column (Thermo Scientific, Waltham, MA, USA) and eluted using a 90-min 8–26% acetonitrile gradient. Mass spectra were acquired with an LTQ Orbitrap XL linear ion trap mass spectrometer (Thermo Scientific) using a data-dependent Top-10 method. Each sample was shot twice in succession, followed by a wash with 70% acetonitrile and 30% isopropanol. MS/MS data were analysed using the Coon OMSSA Proteomics Software Suite^41^. Z-score is representative of the identification of candidate interacting proteins. Total Spectral Count (TSC) is for each identified protein from each immunoprecipitation–MS/MS experiment.

### Cell culture, siRNA and plasmids

Human colon carcinoma HCT-116 (*p53*^+/+^), HCT-116 (*p53*^−/−^) (Dr B. Vogelstein, Johns Hopkins University) and human gastric cancer cell AGS (American Type Culture Collection) were maintained in DMEM (Welgene, Korea) with 10% fetal bovine serum (Hyclone) and 1% antibiotics (Invitrogen, Carlsbad, CA, USA) at 37 °C and 5% CO_2_. Short interfering RNAs (siRNAs) were designed to target PDCD5, p53, HDAC1, HDAC2, HDAC3, HDAC8, MAGE-a2, caspase-3 and caspase-7 mRNA (Supplementary Table 4). Non-targeting siRNA (siCONTROL, Genepharma, Shanghai, China) was used as a control. Cells were transfected with 200 nM chemically synthesized siRNA (Genepharma) using Lipofectamine 2000 (Invitrogen) in 60-mm dishes or 12-well plates. Mammalian expression plasmids for FLAG-HDAC1, Flag-HDAC2, FLAG-HDAC3, FLAG-HDAC8, FLAG-PDCD5, Myc-PDCD5, HA-p53, FLAG-PDCD5 deletion mutants, FLAG-PDCD4 and FLAG-PDCD6 were constructed with standard PCR and cloned into the pSG5 vector (Supplementary Table 4). The FLAG-HDAC3^D391A^, FLAG-HDAC3^1–391^, FLAG-HDAC3^WT^-HA, FLAG-HDAC3^D391A^-HA, FLAG-PDCD5^L6R^, HA-PDCD5^E16D^, shPDCD5-resistant HA-PDCD5^WT^ and shPDCD5-resistant HA-PDCD5^E16D^ expression plasmids were derived from FLAG-HDAC3 and HA-PDCD5 or Myc-PDCD5 using the QuikChange site-directed mutagenesis kit (Stratagene, La Jolla, CA, USA). Bacterial expression plasmids for GST-HDAC3, GST-HDAC3N and GST-HDAC3C were constructed with PCR and subcloned into the pGEX4T-1 vector. Glutathione *S*-transferase (GST)-fusion proteins were expressed in the *Escherichia coli* strain BL21 (DE3) with 0.5 mM isopropyl-β-D-thiogalactoside for 2 h, and then isolated using Glutathione Sepharose 4B beads (Peptron, Korea) according to the manufacturer's instructions. All constructs were verified by DNA sequencing.

### Generation of PDCD5^*flox/flox*^ mice and MEFs

Conditional targeting strategy of *pdcd5* was carried out by the insertion of a loxP site between exon 2 and exon 3. An additional site was inserted upstream of exon 4 (Supplementary Fig. 13). Primers used for genotyping are given below: P1, 5′- CTTGGGACAAACGCTAGTCG -3′; P2, 5′- GGAAACTGGACCTCACCAAA -3′; P3, 5′- GTCGGCTCTATGGCTTCTGA -3′; P4, 5′- GCAGGGGTAAAACAGCAGAG -3′; P5, 5′- CAGTAAATACCCACGGAGTT -3′. All mouse experiments were carried out on the 129S7/SvEvBrd × C57BL/6 mixed background. Wild-type and PDCD5^*f/f*^ MEFs were prepared from mouse embryos of wild-type and PDCD5^*f/f*^ homozygotes as follows: after isolation and genotyping of individual embryos seperately at embryonic day 13.5, MEFs with the same genotype were pooled and maintained until use. Animal studies were performed after obtaining approval according to the guidelines of the Institutional Animal Care committee of CHA Research Institute. Embryo head and internal organs were removed, and the torso was minced. Cells were grown for two population doublings (considered as one passage) and then viably frozen. MEFs were used for all subsequent experiments. MEFs were maintained in DMEM containing 10% fetal bovine serum (Gibco BRL, Gaithersburg, MD, USA) and subcultured 1:3 upon reaching confluence. For electroporation of MEFs, 5 μg of plasmid was electroporated into a suspension of 2 × 10^5^ cells using a Neon transfection system (Life Technologies, Grand Island, NY, USA).

### Xenograft experiments

A suspension of 2 × 10^6^ AGS cells in 100 μl PBS was injected subcutaneously into the right flank of 5-week-old athymic BALB/c nu/nu mice (Orient, Seoul, Korea). Each experimental group included eight mice. Tumour size was monitored closely and measured every 6 days using a caliper. Three weeks after injection, mice with comparable-sized tumours (100∼200 mm^3^) were selected for treatment with ET (10 mg kg^−1^), with 2-day intervals for 8 weeks. For siHDAC3 treatment, HDAC3 siRNA/*in vivo* jetPEI complexes were prepared in a volume of 20 μl per tumour using the *in vivo* jetPEI (Polyplus Transfection, Illkirch, France) delivery reagent (20 μg siRNA/20 μl jetPEI) according to the manufacturer's instructions. The xenografts reaching a tumour volume of 60–80 mm^3^ were injected intratumorally into each animal seven times with 2-day intervals. After 8 weeks of ET treatment, mice were killed and tumours were harvested, photographed and weighed. The volume of tumours was estimated using the formula: volume=½ × *a* × *b^2^*, where *a* and *b* represent the largest and smallest diameters, respectively. Animal studies were performed after obtaining approval according to the guidelines of the Institutional Animal Care committee of the Ulsan College of Medicine.

### Animal experiments

Eight mice per group were used for each experiment. Mice were injected with adenovirus for GFP or Cre recombinase via tail vein injection. A dosage of 1 × 10^9^ plaque-forming units per mouse was used for adenovirus delivery. Mice were injected with ET (10 mg kg^−1^) from the 6th day post adenovirus injection for the indicated days. For the restoration experiment using PDCD5 adenovirus, a dosage of 1 × 10^9^ plaque-forming units per mouse was injected from the 2nd day post Cre adenovirus injection. Mice were anesthetized using ketamine (80 mg kg^−1^) and xylaxine (10 mg kg^−1^) by intraperitoneal injection. Total protein and RNA were isolated from individual livers, and western blot analysis and quantitative reverse transcription (qRT)–PCR were performed as described above. Animal studies were performed after obtaining approval according to the guidelines of the Institutional Animal Care committee of CHA Research Institute.

### Gene expression data

Experiments and analysis for 78 samples from the YUSH data set were conducted in the Yonsei University Severance Hospital. All specimens from patients were collected and archived under the standard protocols with codified patient identification information. The need for informed consent for the gene expression analysis was waived by the institutional review board of Severance Hospital Yonsei University Health System, as the current study was considered a retrospective review of anonymized clinical data. The information on clinic-pathological characteristics of cancer patients was described in Supplementary Table 5. Gene expression profiles were generated by hybridizing labelled cRNAs to Illumina human bead arrays (HumanHT-12, v3.0) containing 48,803 gene features. Total RNA was extracted from the fresh frozen tissues using a mirVana RNA isolation labeling kit (Ambion, Inc. Grand Island, NY, USA). Total RNA (500 ng) was used for labelling and hybridization, according to the manufacturer's protocol. After the bead chips were scanned with an Illumina BeadArray Scanner, the microarray data were normalized with a quantile normalization method in the Linear Models for Microarray Data (LIMMA) package in the R language environment^42^. Kaplan–Meier plots and log-rank tests were used to estimate the prognostic differences of categorized patient groups.

### Duolink *in situ* proximity ligation assay (PLA)

Duolink *in situ* PLA analysis was performed according to the manufacturer's instructions (OLink Biosciences, Uppsala, Sweden). In short, paraformaldehyde-fixed cells were washed with PBS, incubated for 15 min in 1.5% hydrogen peroxide, washed and blocked with blocking solution. Primary rabbit antibody was applied, and the cells were incubated with PLUS and MINUS secondary PLA probes against rabbit IgG only or against both rabbit and mouse IgG. After the sample was incubated, hybridization, ligation and amplification steps were performed. Samples were mounted with Duolink mounting medium, and then examined using a Zeiss LSM700 confocal microscope (Carl Zeiss, Oberkochen, Germany).

### HDAC activity assay

Briefly, in the standard assay, 5 *μ*g histone H4 tail peptides were incubated with 0.25 μCi of [^3^H] acetyl coenzyme A (Amersham) in 20 μl reaction buffer containing 50 mM Tris (pH 8.0), 5% glycerol, 0.1 mM EDTA, 50 mM KCl, 1 mM dithiothreitol (DTT), 1 mM phenylmethylsulphonyl fluoride and 10 mM sodium butyrate at 30 °C for 2 h. Immunoprecipitated HDAC3 samples were incubated with ^3^H-acetate-labelled H4 histone in 150 μl of reaction buffer (20 mM Tris at pH 8.0, 150 mM NaCl and 10% glycerol) overnight at room temperature. Reactions were quenched with 1 M HCl and 0.16 M acetic acid (50 μl in each sample). Released ^3^H-acetic acid was extracted with 600 μl ethyl acetate by vortexing and centrifugation (1 min at 9,300*g*). Ethyl acetate supernatants (300 μl from each sample) were quantified by scintillation counting. The results are shown as mean±s.d. calculated from three independent experiments.

### RNA isolation and quantitative RT–PCR

PCR primers for qRT–PCR are described in Supplementary Table 4. The concentration of complementary DNA was normalized using GAPDH. QPCR analysis and quantification was performed using SYBR Green PCR master mix reagents and an ABI Prism 7700 sequence detection system (Applied Biosystems, Carlsbad, CA, USA). All reactions were performed in triplicate. Relative expression levels and s.d. were calculated using the comparative method. The results are shown as mean±s.d. calculated from three independent experiments.

### Chromatin immunoprecipitation (ChIP) assay

Briefly, ∼2 × 10^8^ cells in 100-mm dishes were first treated with PBS containing 1% formaldehyde for 10 min, washed twice with cold PBS and then incubated with 100 mM Tris (pH 9.4) and 10 mM DTT at 30 °C for 15 min. The cells were then rinsed twice in PBS and resuspended in 600 μl of SolA (10 mM Hepes (pH 7.9), 0.5% NP-40, 1.5 mM MgCl_2_, 10 mM KCl and 0.5 mM DTT) by pipetting. After a short spin, the pellets were resuspended in SolB (20 mM Hepes (pH 7.9), 25% glycerol, 0.5% NP-40, 0.42 M NaCl, 1.5 mM MgCl_2_ and 0.2 mM EDTA) containing protease inhibitors followed by vigorous pipetting to extract nuclear proteins. After centrifugation at 13,000 r.p.m. for 30 min, the nuclear pellets were resuspended in immunoprecipitation buffer (1% Triton X-100, 2 mM EDTA, 20 mM Tris-HCl (pH 8.0), 150 mM NaCl and protease inhibitors) and sonicated to break chromatin into fragments of 0.5–1 kb average length. The ChIP assays were then preformed with the indicated antibodies essentially as described, but without SDS in all buffers. Primers were used for ChIP assays are described in Supplementary Table 4. All reactions were normalized relative to input activities and are presented as mean±s.d. of three independent experiments. The results are shown as percentage of input.

### TUNEL assay

For the detection of apoptosis in cells, DNA fragmentation was evaluated by the TdT-mediated dUTP nick end labelling (TUNEL) assay using an HT Titer TACS Assay Kit (Cat No. 4822-96-K, Trivigen, Gaithersburg, MD, USA) according to the manufacturer's instructions. Briefly, cells were fixed with a 3.7% buffered formaldehyde solution for 7 min, washed with PBS, permeabilized with 100% methanol for 20 min and then washed twice with PBS. Samples were digested with proteinase K for 15 min, and then quenched with 3% hydrogen peroxide. Samples were then washed with distilled water, labelled with deoxynucleotidyl transferase at 37 °C for 90 min and then treated with stop buffer. The cells were incubated with TACS-Sapphire substrate, and the colorimetric reaction was stopped with 0.2 N HCl after 30 min. Absorbance was measured with a microplate reader at 450 nm. The results are shown as mean±s.d. calculated from three independent experiments.

### Annexin V-FITC staining

To assess the extent of apoptosis after DNA damage, cells were stained with both Annexin V-fluorescein isothiocyanate (FITC) and propidium iodide using the ApoScan Annexin V-FITC Apoptosis Detection Kit (BioBud, Korea) according to the manufacturer's protocol. Alternatively, cells were fixed in 70% (v/v) ethanol and stained with a solution containing RNase A (50 μg ml^−1^) and propidium iodide (50 μg ml^−1^).

### Adenoviruses and lentiviral shRNAs

For stable knockdown of *PDCD5* gene expression, two pairs of oligonucleotides (5′- CCGGUGAUGAAGAUGACGAUUAUUGCUCGAGCAAUAAUCGUCAUCUUCAUCAUUUUG -3′) that encoded the shRNA against each target MISSION shRNA were purchased (Sigma-Aldrich). To generate lentiviral particles, pLKO.1-PURO PDCD5 plasmid with three plasmids (pMDLg/pRRE, envelope pRSV-REV and pMD2.G) were co-transfected using Lipofectamine 2000 (Invitrogen, Grand Island, NY, USA) in 293FT cell line. After 48 h incubation, supernatants were collected and filtered using a 0.45-μm pore. HCT-116 line was then infected with lentivirus particles. After incubation with virus supernatant for 2 days, cells were selected with 1 μg ml^−1^ of puromycin (Sigma-Aldrich). Lentivirus PURO shRNA was generated as a control. Recombinant adenoviruses expressing GFP and Cre recombinase were generated as described^43^. These were constructed into an E1 shuttle vector, and then linearized with PmeI. The linearized vector was cotransformed into *E. coli* BJ 5,183 along with pAdEasy1 vector. All viruses were propagated in 293A cells and purified by CsCl density purification. Viral particles were calculated at 260 nm absorbance. The multiplicity of infection was calculated from viral particle numbers^44^.

### Cell fractionation

Cells were cultured on 100-mm plates and washed twice with ice-cold PBS. Harvested cells were homogenized in buffer A (250 mM sucrose, 10 mM KCl, 1.5 mM MgCl_2_, 1 mM EDTA, 1 mM EGTA, 1 mM DTT, 0.1 mM phenylmethylsulphonyl fluoride, 20 mM HEPES (pH 7.5) and protease inhibitor cocktail (Roche)) using a Teflon pestle (Thomas Scientific) and then centrifuged at 1,000*g* at 4 °C for 10 min. Pellets were used as nuclear fractions while post-nuclear supernatant fractions were centrifuged at 10,000*g* at 4 °C for 10 min to obtain mitochondria. The pellets containing mitochondria were washed with buffer A and resuspended in TXIP-1 buffer (1% Triton X-100, 150 mM NaCl, 0.5 mM EDTA and 50 mM Tris-HCl (pH 7.4)) containing a protease inhibitor cocktail. The post-mitochondrial supernatant was centrifuged for 30 min at 100,000*g* to obtain cytosolic protein fractions.

### Immunofluorescence staining

Cells were cultured on coverslips. Cells were fixed in 4% paraformaldehyde for 30 min at 4 °C, and then treated with 0.4% Triton X-100 in PBS for 10 min at room temperature. For detection of endogenous HDAC3, cells were incubated with anti-HDAC3 antibody at 37 °C for 2 h, and then stained with goat anti-rabbit rhodamine (Invitrogen) at 37 °C for 1.5 h. DNA was revealed with Hoechst 33258 staining. Cells were imaged using a confocal microscope.

### Statistical analysis

Statistical analyses were performed using a Student's *t*-test with Bonferroni correction for multiple comparisons. A *P* value of less than 0.05 was considered statistically significant.

## Additional information

**How to cite this article:** Choi, H.-K. *et al*. Programmed cell death 5 mediates HDAC3 decay to promote genotoxic stress response. *Nat. Commun.* 6:7390 doi: 10.1038/ncomms8390 (2015).

## Supplementary Material

Supplementary Information Supplementary Figures 1-16, Supplementary Tables 1-5
