# Corrigendum: Programmed cell death 5 mediates HDAC3 decay to promote genotoxic stress response

**DOI:** 10.1038/ncomms9225

**Published:** 2015-08-21

**Authors:** Hyo-Kyoung Choi, Youngsok Choi, Eun Sung Park, Soo-Yeon Park, Seung-Hyun Lee, Jaesung Seo, Mi-Hyeon Jeong, Jae-Wook Jeong, Jae-Ho Cheong, Peter C. W. Lee, Kyung-Chul Choi, Ho-Geun Yoon

Nature Communications 6: Article number: 7390 (2015); 10.1038/ncomms8390 Published: 06162015; Updated 08212015.

The original version of this Article contained a typographical error in the spelling of the author Jae-Ho Cheong, which was incorrectly given as Jae-Ho Jeong. This has now been corrected in both the PDF and HTML versions of the Article.

