# Supplementary material for: Programmed cell death 5 mediates HDAC3 decay to promote genotoxic stress response
Source: Nat Commun. 2015 Jun 16;6:7390. doi: 10.1038/ncomms8390 (PMC4490383; doi:10.1038/ncomms8390)
Supplement: Supplementary Information — Supplementary Figures 1-16, Supplementary Tables 1-5 [file ncomms8390-s1.pdf]

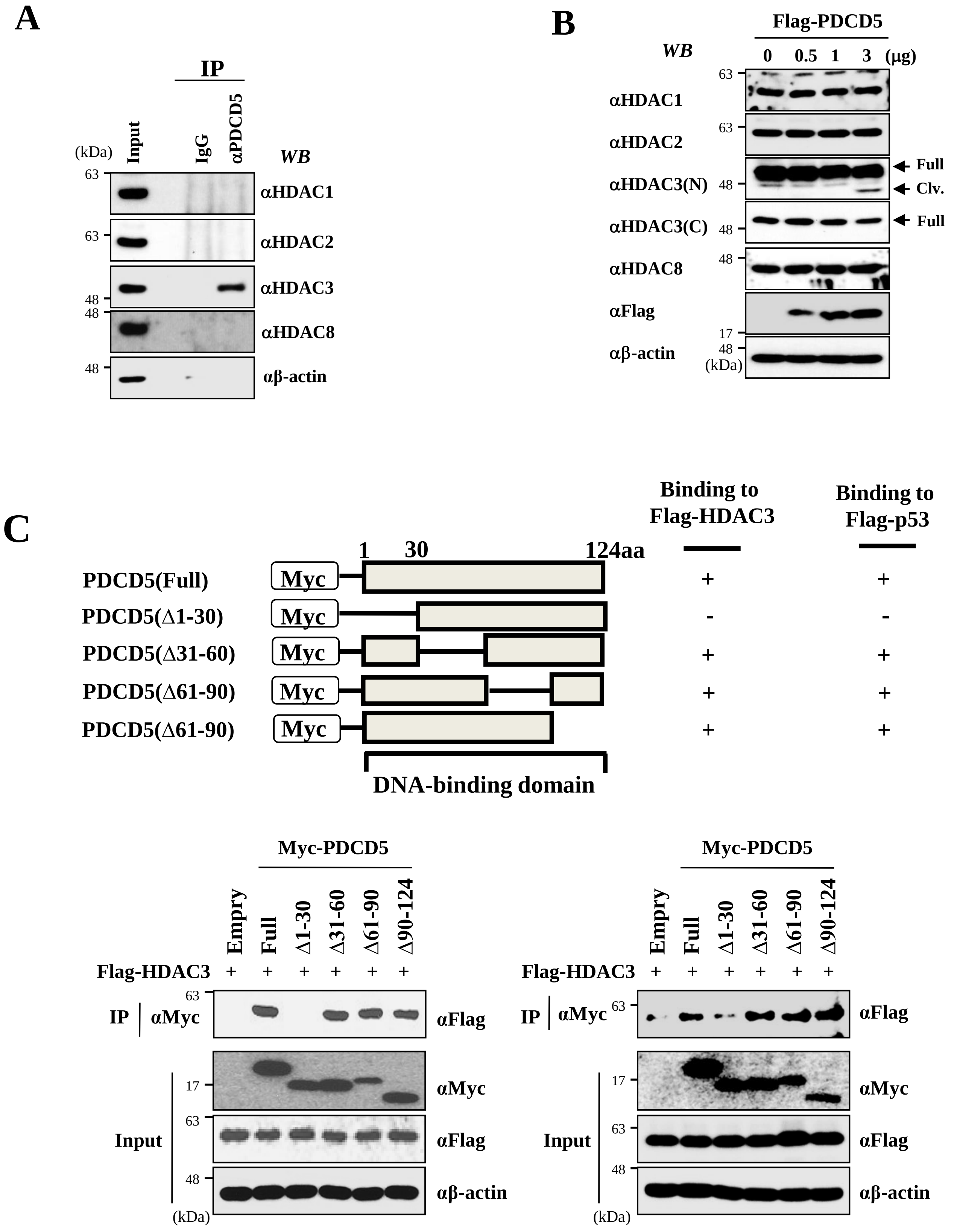

A
IP
(kDa)
Input
IgG
αPDCD5
WB
63
αHDAC1
63
αHDAC2
48
αHDAC3
48
αHDAC8
48
αβ-actin
B
Flag-PDCD5
WB
0 0.5 1 3 (μg)
63
αHDAC1
63
αHDAC2
Full
αHDAC3(N)
48
Clv.
αHDAC3(C)
48
Full
48
αHDAC8
αFlag
17
48
αβ-actin
(kDa)
C
Binding to Flag-HDAC3
Binding to Flag-p53
1
30
124aa
PDCD5(Full)
PDCD5(Δ1-30)
PDCD5(Δ31-60)
PDCD5(Δ61-90)
PDCD5(Δ61-90)
Myc
+
-
DNA-binding domain
Myc-PDCD5
Empry
Full
Δ1-30
Δ31-60
Δ61-90
Δ90-124
Flag-HDAC3
+ + + + + +
IP
αMyc
63
αFlag
17
αMyc
Input
63
αFlag
48
αβ-actin
(kDa)

D

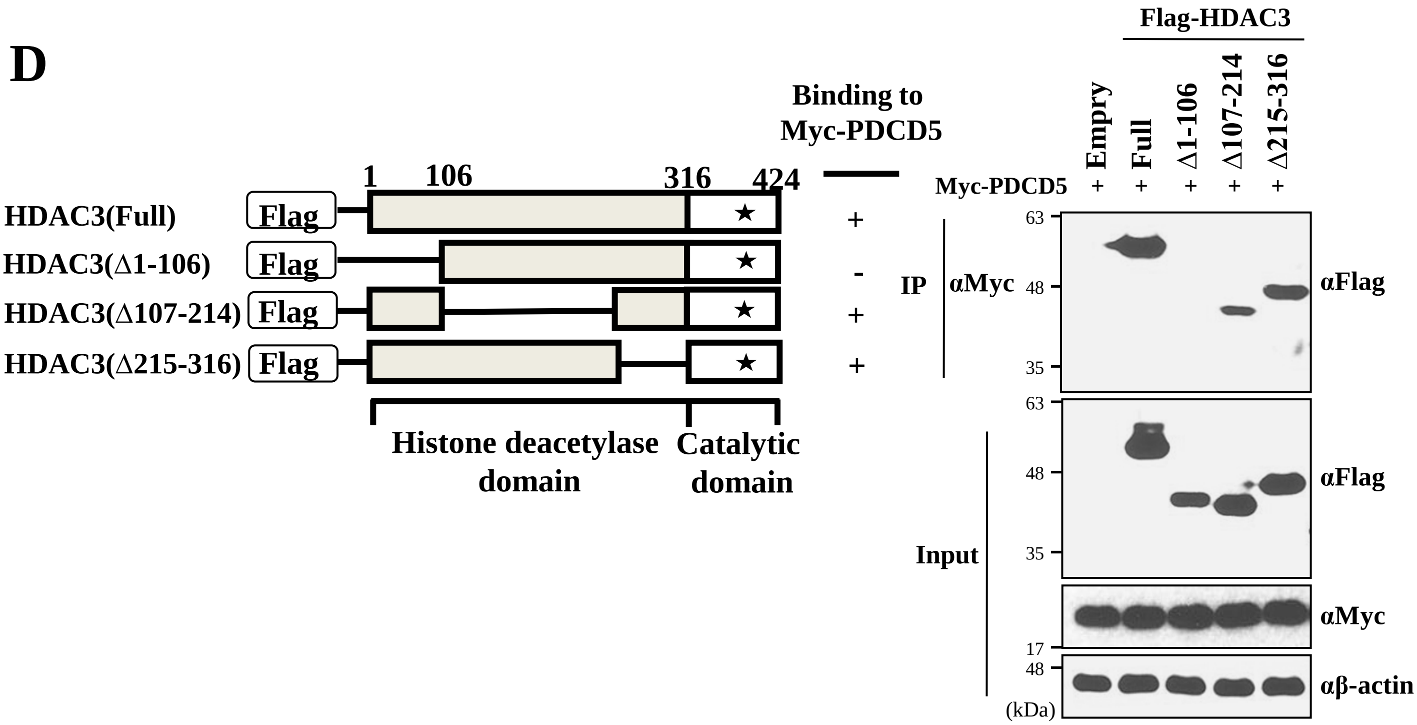

Supplementary figure 1. The N-terminal domain of PDCD5 (1-30 aa) interacts with the histone deacetylase domain (1-106 aa) of HDAC3 and p53. (A) PDCD5 selectively interacts with HDAC3 in A2780 ovarian cancer cell. Proteins from A2780 whole cell lysate were immunoprecipitated and subsequently immunoblotted with the indicated antibodies. (**B**) Overexpression of PDCD5 induces C-terminal cleavage of HDAC3 in A2780 cells. Cells were transfected with the increasing amounts of Flag-PDCD5 plasmid. Whole cell lysate were immunoblotted with the indicated antibodies. Arrow indicates cleaved HDAC3. (**C-D**) Schematic diagrams of Myc-tagged PDCD5 (**C**) and Flag-tagged HDAC3 (**D**) deletion mutants for co-immunoprecipitation and mapping analysis. HCT116 (*p53*$^{+/+}$) cells were cotransfected with indicated Myc-PDCD5 and Flag-HDAC3 or Flag-p53 plasmids. Cell lysates were immunoprecipitated with Flag antibodies, and subsequently immunoblotted with the indicated antibodies.

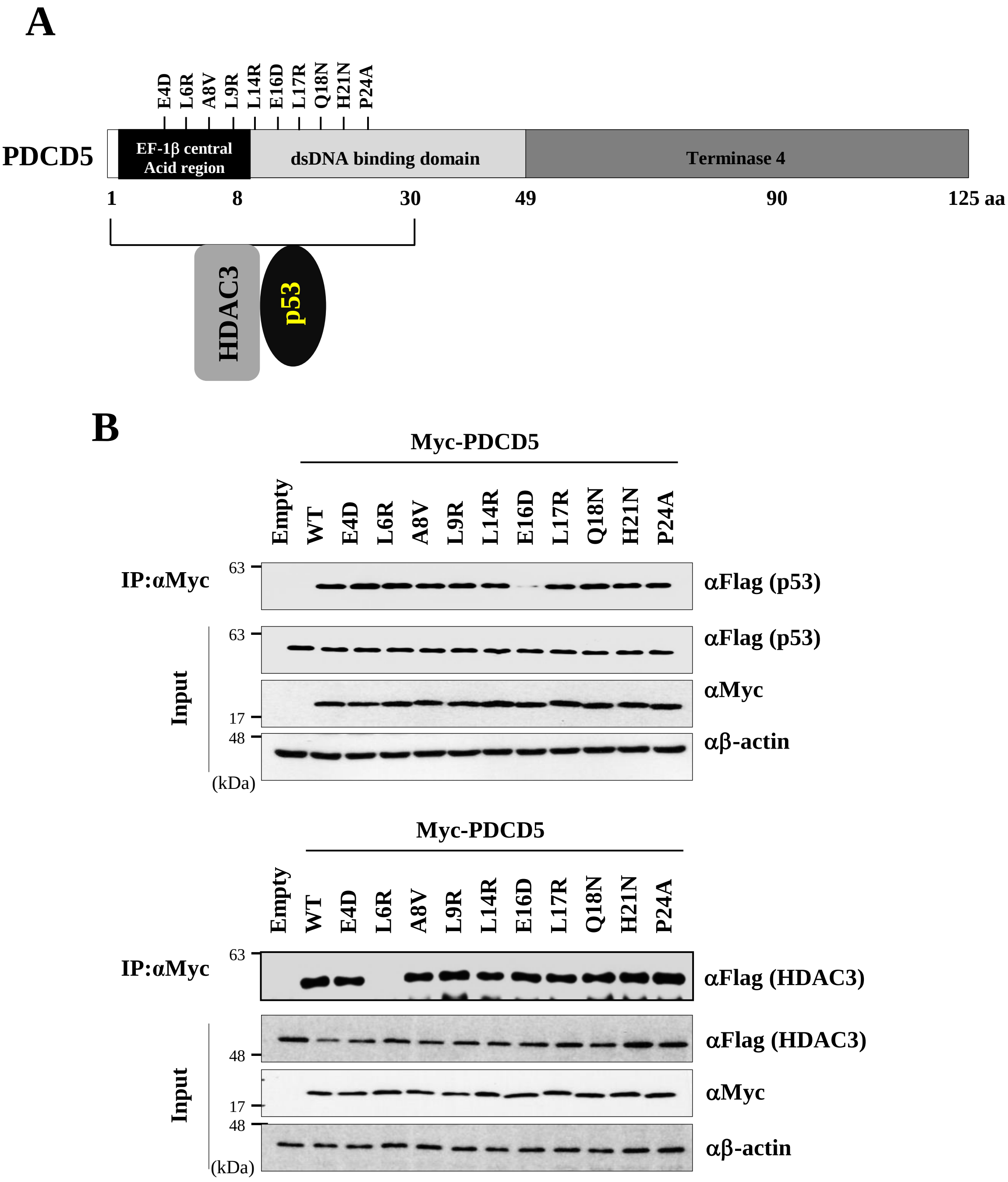

Supplementary figure 2. Generation of various PDCD5 point mutants by site-directed mutagenesis. (**A**) Schematic diagrams of PDCD5 mutants. (**B-C**) Flag-tagged mutant PDCD5 plasmids in which the indicated amino acid residue was substituted for indicated amino acids were screened generated by site-directed mutagenesis. To select the p53-interacting, PDCD5-defective mutant and the HDAC3-interacting, PDCD5-defective mutant, each Myc-PDCD5 plasmid was co-transfected with Flag-p53 (**B**) or Flag-HDAC3 (**C**) into HCT116 cells. Whole cell lysates were immunoprecipitated with anti-Myc antibody and subsequently immunoblotted with indicated antibodies.

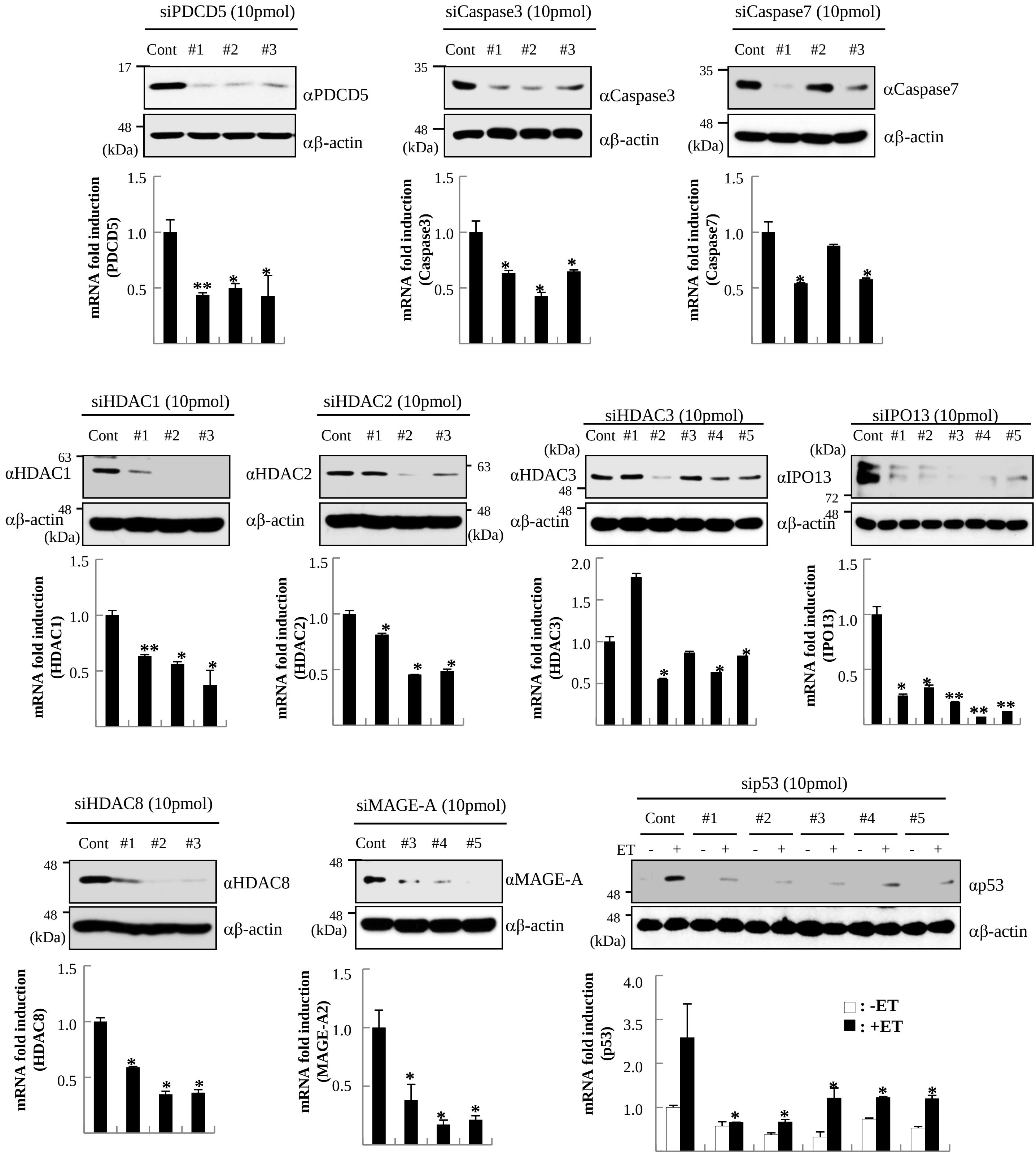

Supplementary figure 3. Validation of siRNAs. HCT116 cells were transfected with indicated sets of plasmids. Whole cell lysates were analyzed by western blotting with the indicated antibodies. Total RNA was isolated from individual cells, and qRT-PCR was performed for the indicated genes. (* *P*<0.05 vs sicon; ***P*<0.01 vs sicon). Error bars, SD (n=3). SD, Standard deviation.

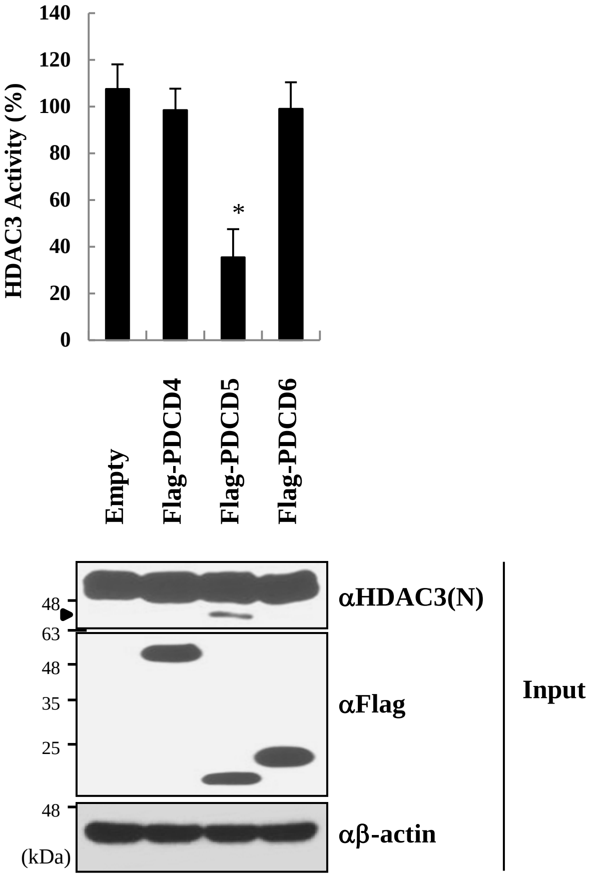

Supplementary figure 4. PDCD5 selectively reduces HDAC3 activity. HCT116 cells were transfected with indicated plasmids. Whole cell lysates were immunoblotted with indicated antibodies. Whole cell lysates were immunoprecipitated with anti-HDAC3 (N) antibody, and then HDAC3 activity was measured. Error bars, SD (n=3). **P*<0.05 vs. empty plasmid.

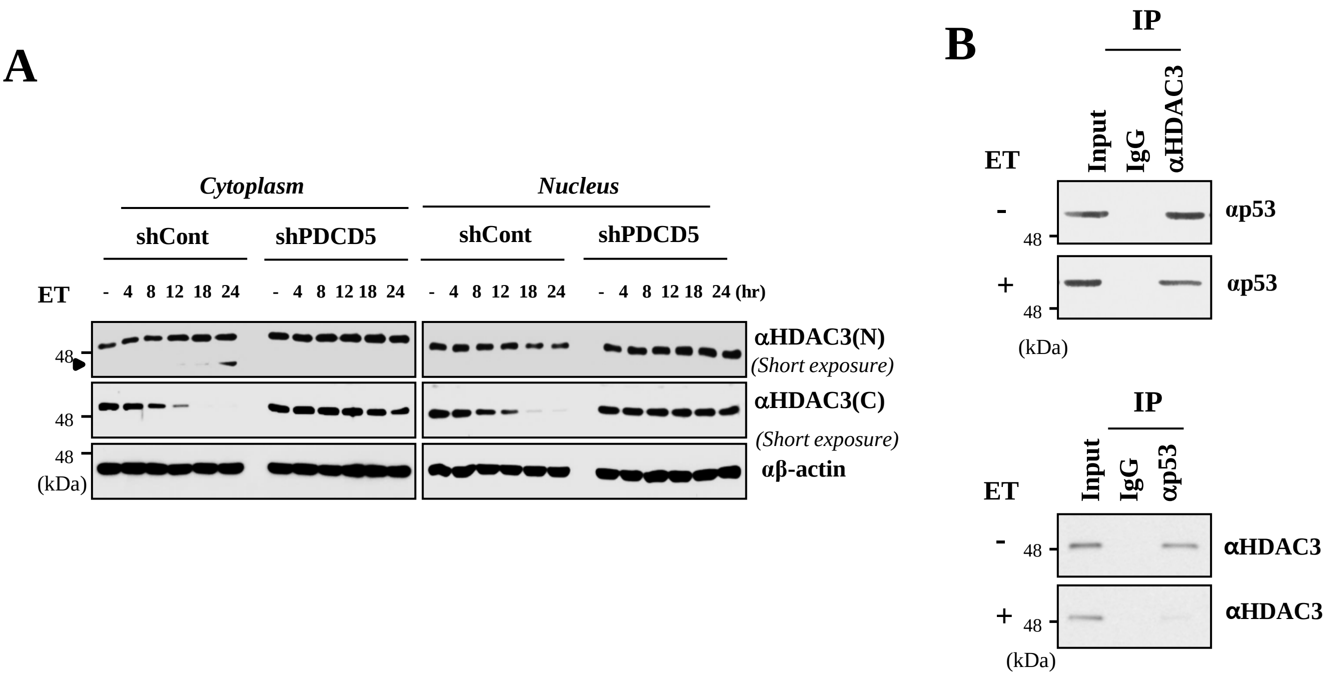

Supplementary figure 5. PDCD5 mediates ET-induced cytosolic translocation of HDAC3 and dissociation of HDAC3 from p53. (**A**) HCT116 cells were treated with ET (100 μM) for 24 hr. Following cell fractionation, each fraction was analyzed by immunoblotting with the indicated antibodies. (**B**) HCT116 cells were treated with ET. Cell lysates were immunoprecipitated with p53 or HDAC3 antibodies and subsequently analyzed by immunoblotting with the indicated antibodies.

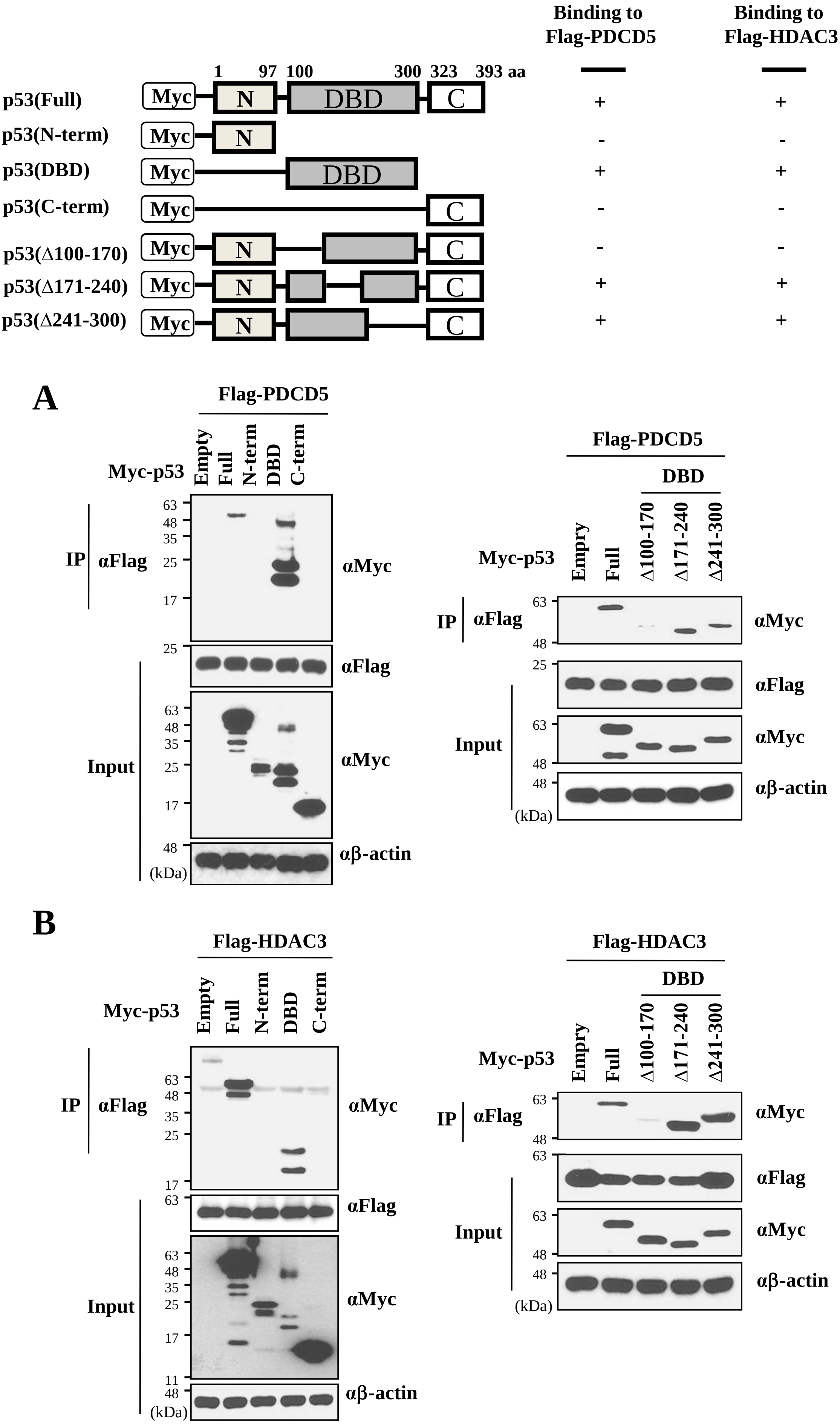
Binding to Flag-PDCD5
Binding to Flag-HDAC3
p53(Full) + +
p53(N-term) - -
p53(DBD) + +
p53(C-term) - -
p53(Δ100-170) - -
p53(Δ171-240) + +
p53(Δ241-300) + +
1 97 100 300 323 393 aa
Myc N DBD C
A
Flag-PDCD5
Myc-p53 Empty Full N-term DBD C-term
IP αFlag αMyc
Input αFlag αMyc αβ-actin
Myc-p53 Empry Full DBD Δ100-170 Δ171-240 Δ241-300
(kDa)
B
Flag-HDAC3
Myc-p53 Empty Full N-term DBD C-term
IP αFlag αMyc
Input αFlag αMyc αβ-actin
Myc-p53 Empry Full DBD Δ100-170 Δ171-240 Δ241-300
(kDa)

Supplementary figure 6. HDAC3-binding domain in p53 (100-170 a.a.) is identical to that in PDCD5. Schematic diagrams of Myc-p53 deletion mutants for co-immunoprecipitation and mapping analysis. HCT116 cells were co-transfected with Myc-p53 and Flag-PDCD5 plasmids (**A**) or Flag-HDAC3 plasmids (**B**). Cell lysates were immunoprecipitated with Flag antibodies, and subsequently analyzed via immunoblotting with the indicated antibodies.

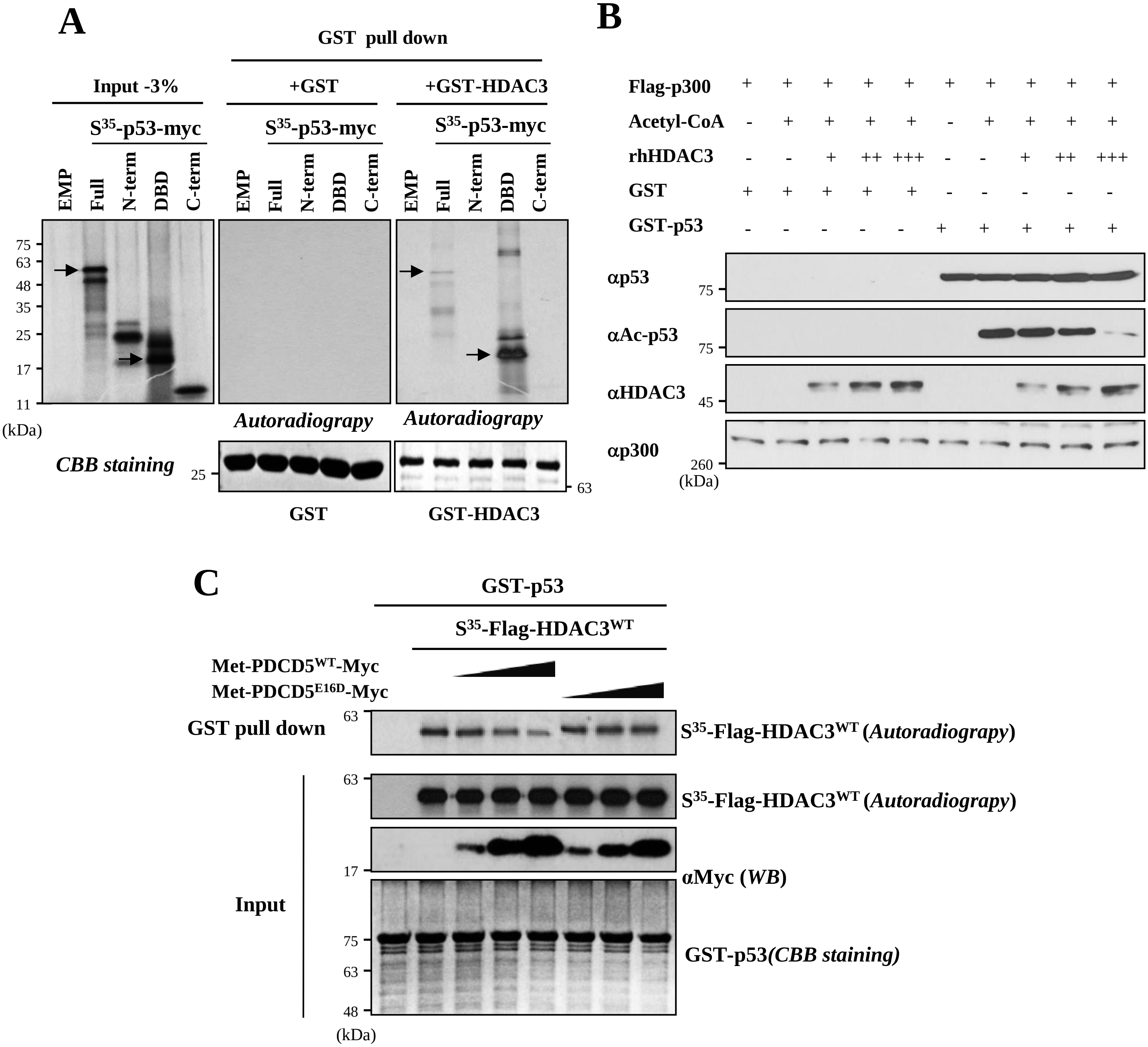

Supplementary figure 7. PDCD5 competitively inhibits HDAC3-mediated p53 deacetyaltion. (**A**) HDAC3 directly binds p53. GST pull-downs were performed overnight at 4°C in binding buffer. Bound proteins were eluted and analyzed by autoradiography. (**B**) HDAC3 directly deacetylates p53. HCT cells were transfected with Flag-p300 plasmid. Cell lysates were immunoprecipitated with Flag antibodies. Individual reactions contained immunoprecipitated Flag-p300 proteins and GST-p53 proteins in 50 μl of assay buffer with 10 μM acetyl CoA. Acetylation reactions were incubated for 60 min at 30°C on a rotating platform. Recombinant human HDAC3(rhHDAC3) proteins were added after washing with deacetylation buffer as described in the section of HDAC activity assay. The reaction was stopped by addition of SDS-PAGE sample buffer and electrophoresis through 10% SDS-PAGE gels. The levels of p53 acetylation were assessed by immunoblotting assay. (**C**) PDCD5 competitively inhibits the binding of HDAC3 to p53. The binding of ^{35}S-Met labeled HDAC3 to GST-p53 was challenged with an increasing amount of in vitro translated-PDCD5^{WT} or PDCD5^{E16D} mutant. GST pull-downs were performed overnight at 4°C in binding buffer. Bound proteins were eluted and analyzed by autoradiography.

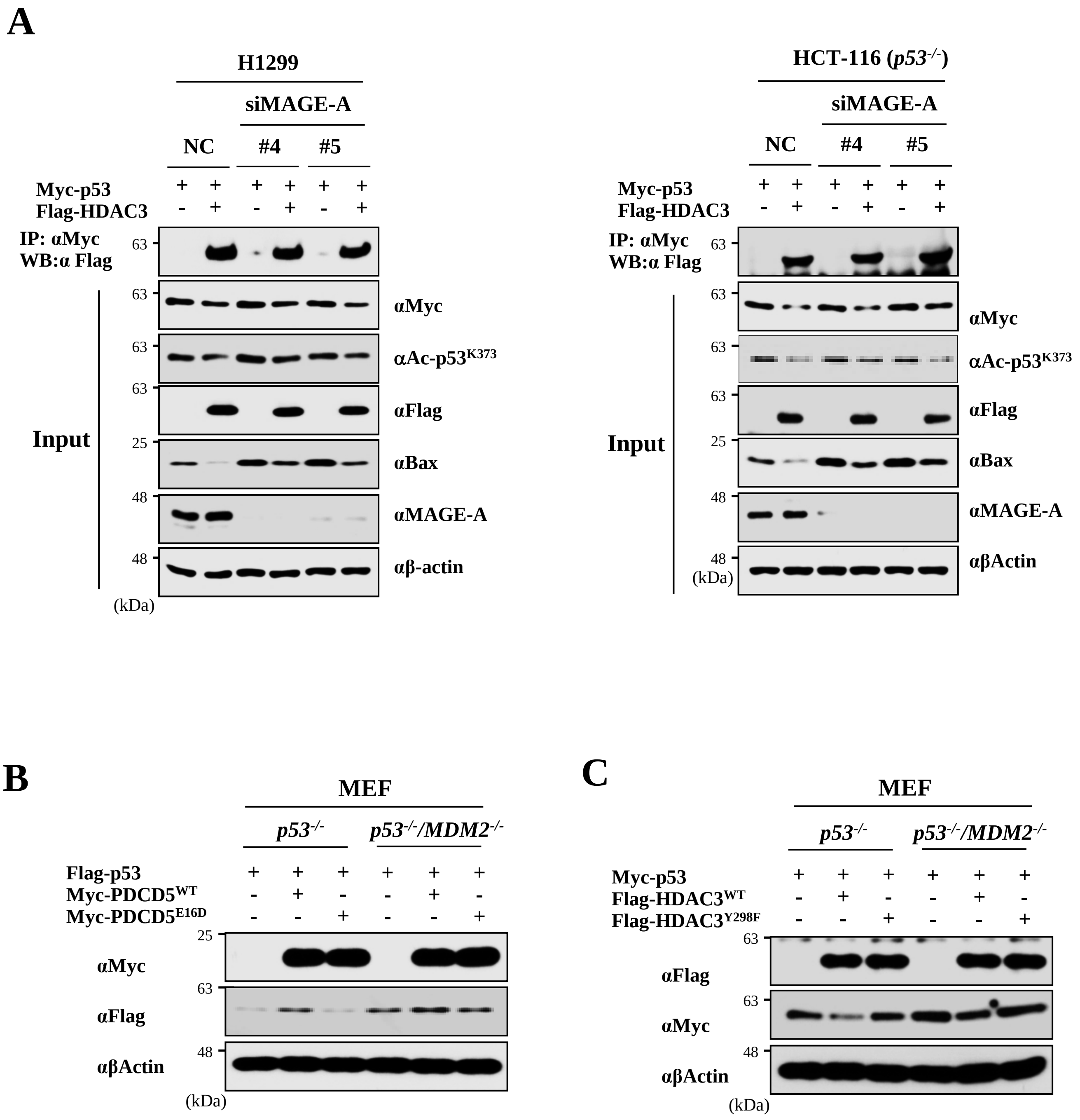

Supplementary figure 8. HDAC3 suppresses p53 stabilization independently of MDM2 and Mage-A2. (**A**) Either H1299 cell or HCT-116 cell was transfected with indicated plasmids and/or siRNAs against Mage-A. Cell lysates were immunoprecipitated with Myc or Flag antibody, and subsequently analyzed via immunoblotting with the indicated antibodies. (**B-C**) MEFs were electroporated with indicated plasmids, and cell lysates were analyzed by immunoblotting.

Supplementary Figure 9

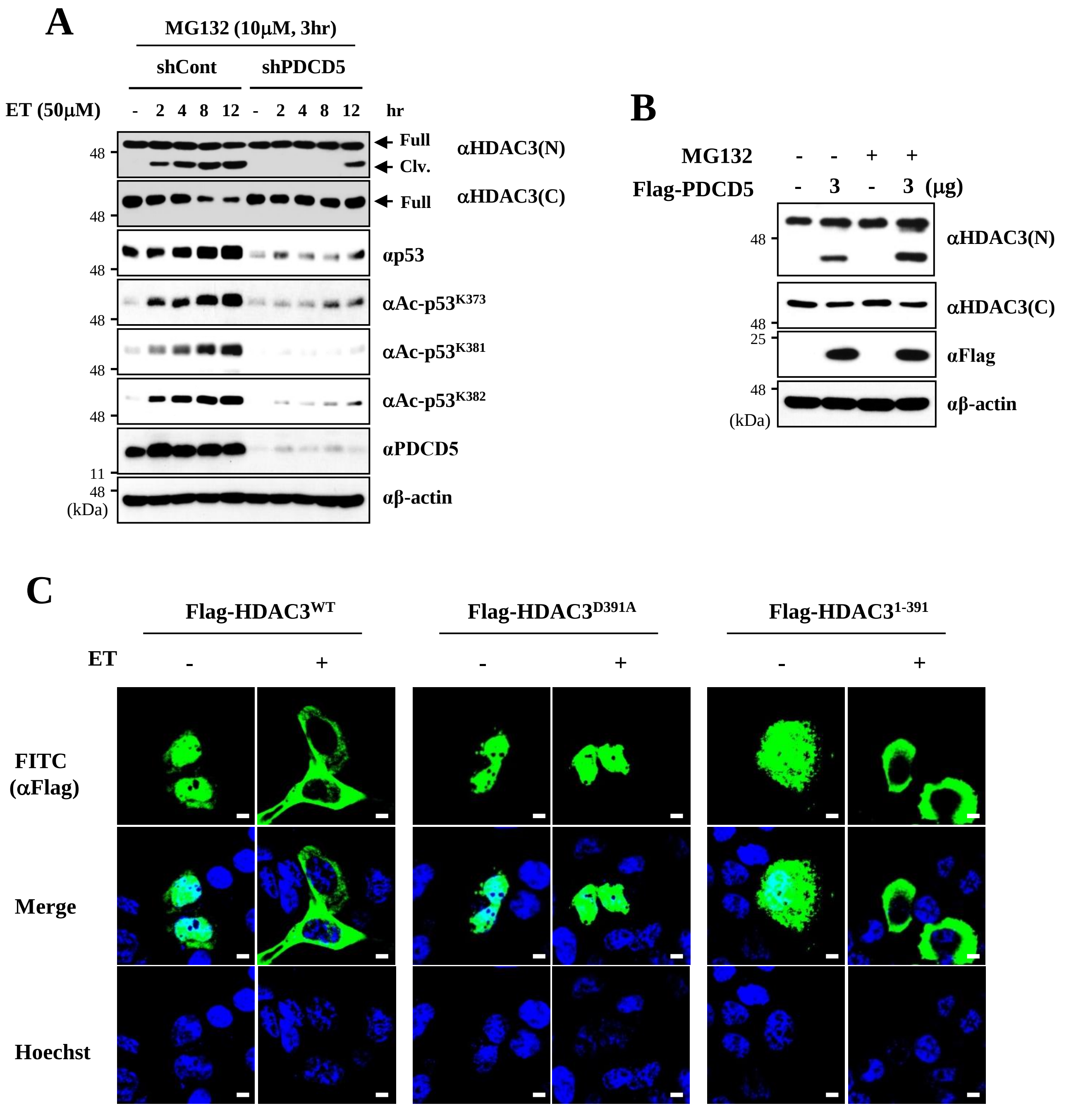

Supplementary figure 9. PDCD5-medaited HDAC3 cleavage leads to proteosomal degradation of HDAC3. (**A**) PDCD5 knockdown diminishes the cleavage of full-length HDAC3 and p53 acetylation. Cells were treated with ET (50 μM) and/or MG132 (10 μM, 3 h). Whole-cell lysates were immunoblotted with the indicated antibodies. (**B**) MG132 treatment induces accumulation of cleaved HDAC3 by PDCD5 overexpression. Cells were were transfected with the indicated plasmids, and treated with ET and/or MG132. Whole-cell lysates were immunoblotted with the indicated antibodies. (**C**) Mutation of Asp-391 abolishes ET-induced nuclear export of HDAC3. HCT-116 cells were transfected with the indicated Flag-HDAC3 plasmids and treated with ET. Immunofluorescence analysis was performed as described in the Supplemental Experimental Procedures section. Scale bar, 10μm.

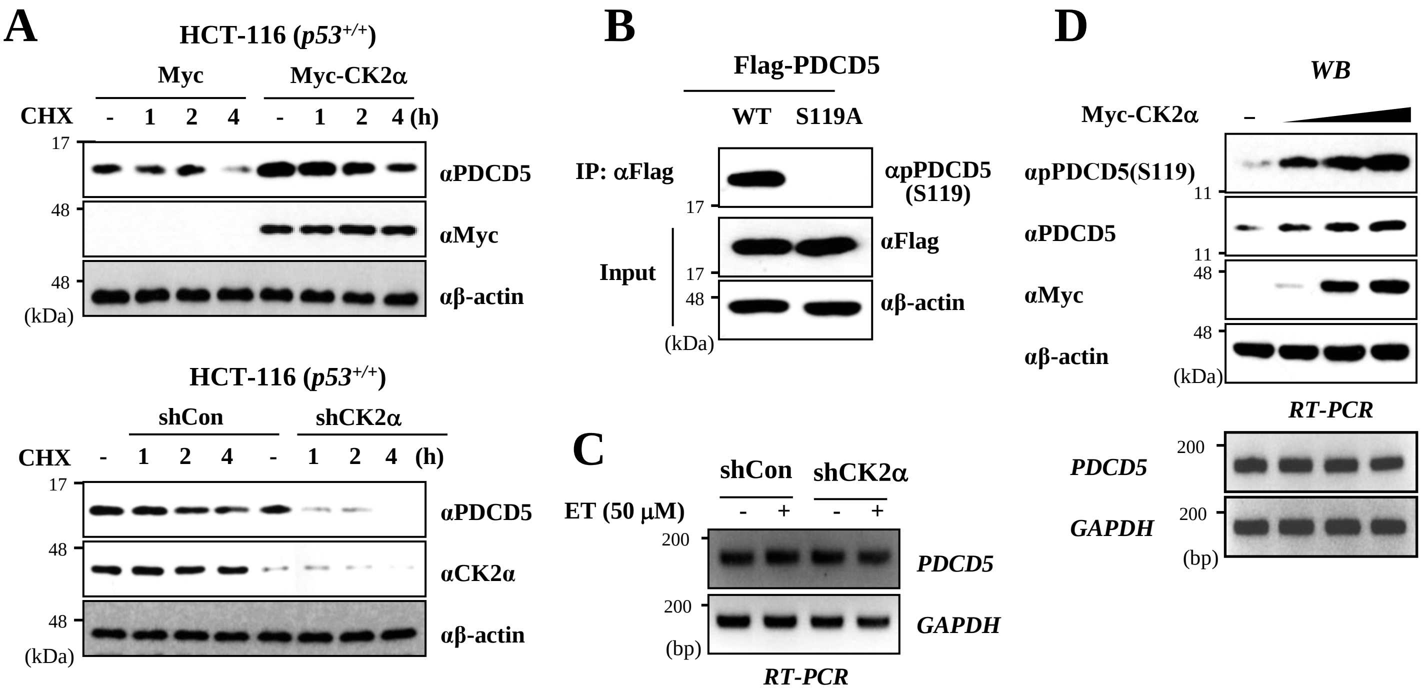

Supplementary figure 10. Casein kinase 2–mediated phosphorylation enhances PDCD5 stability. (**A**) CK2α enhances PDCD5 stability. HCT-116 ($p53^{+/+}$) cells were treated with cycloheximide (CHX) and Flag-CK2α plasmid (upper panel) or shCK2α (lower panel) during the indicated time periods, and cell lysates were analyzed by western blotting. (**B**) Validation of phospho-specific PDCD5 antibody that specifically recognizes phosphorylated Ser-119. Either Flag-$PDCD5^{WT}$ or Flag-$PDCD5^{S119A}$ was transfected into HCT-116 cells, and cell lysates were analyzed by western blotting with phospho-specific $PDCD5^{S119}$ antibody. (**C**) CK2α knockdown had no effects on *PDCD5* mRNA levels. shControl or stable shCK2α-expressing HCT-116 cells were treated with ET. *PDCD5* mRNA was analyzed by RT-PCR. (**D**) CK2a increases PDCD5 protein levels, but not *PDCD5* mRNA levels, in a dose-dependent manner. Cells were transfected with increasing amounts of Myc-CK2. Whole-cell lysates were immunoblotted with the indicated antibodies (upper panel). *PDCD5* mRNA was analyzed by RT-PCR (lower panel).

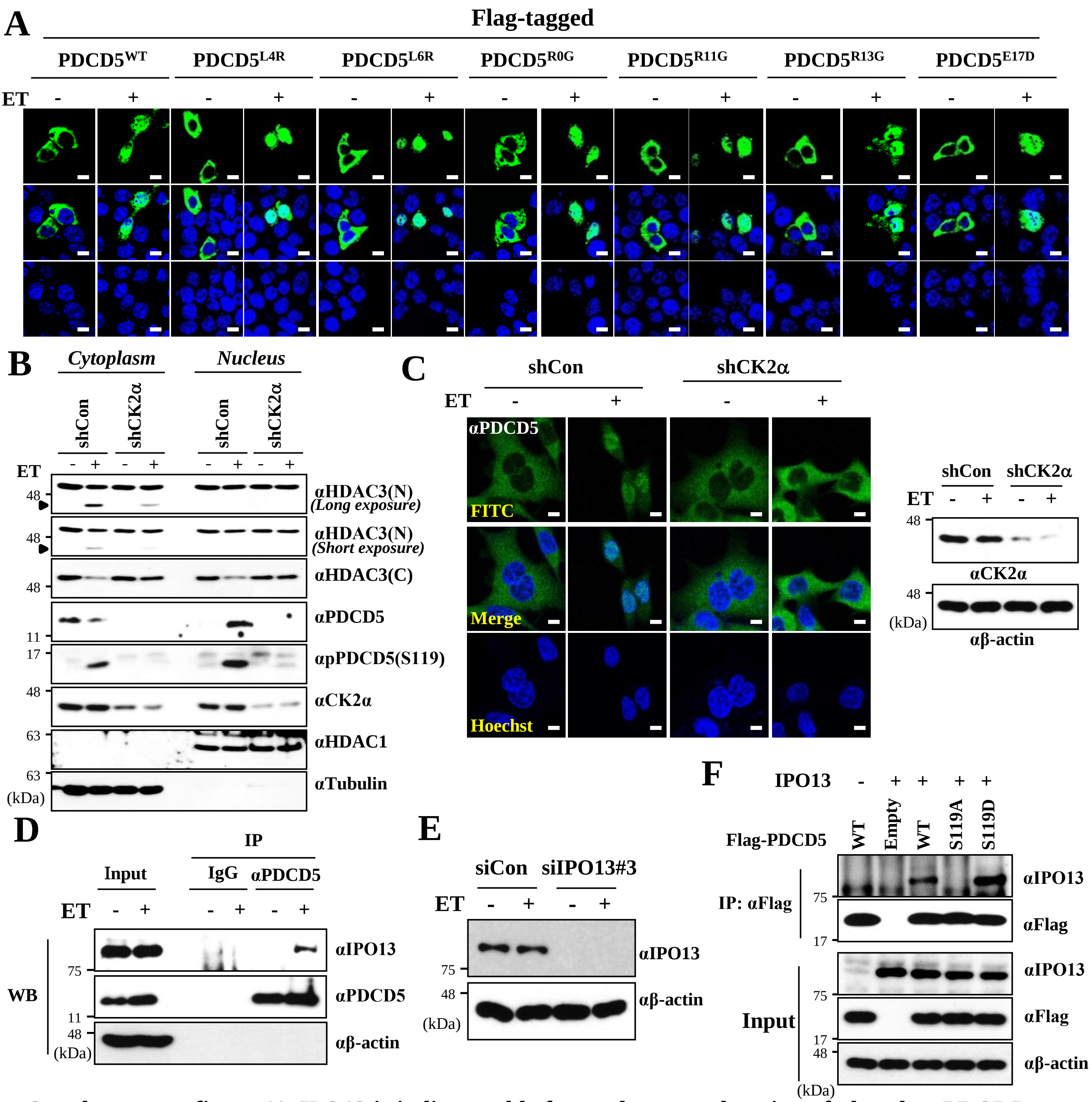

Supplementary figure 11. IPO13 is indispensable for nuclear translocation of phosphor-PDCD5 upon genotoxic stress response. (A) Cells were transfected with wild-type or the indicated PDCD5 mutant plasmids, and treated with ET (50 μM). Immunofluorescence analysis was performed as described in the Supplemental Experimental Procedures section. (**B**) CK2α is required for nuclear translocation of PDCD5. shControl or stable shCK2α-expressing HCT-116 cells were treated with ET. Following cell fractionation, fractions were immunoblotted with the indicated antibodies. (**C**) Knocking-down of CK2α blocked the nuclear translocation of PDCD5. Cells were treated with indicated siRNAs and analyzed by Immunofluorescence assays (left panel). Validation of shRNA against CK2α (right panel). (**D**) IPO13 interacts with PDCD5 in response to ET. Whole-cell lysates were immunoprecipitated and immunoblotted with the indicated antibodies. (**E**) Validation of siRNA against IPO13. (**F**) IPO13 strongly binds to phospho-mimetic PDCD5^{S119D} when compared with wild-type PDCD5. Whole-cell lysates were immunoprecipitated and immunoblotted with the indicated antibodies. Scale bar, 10μm.

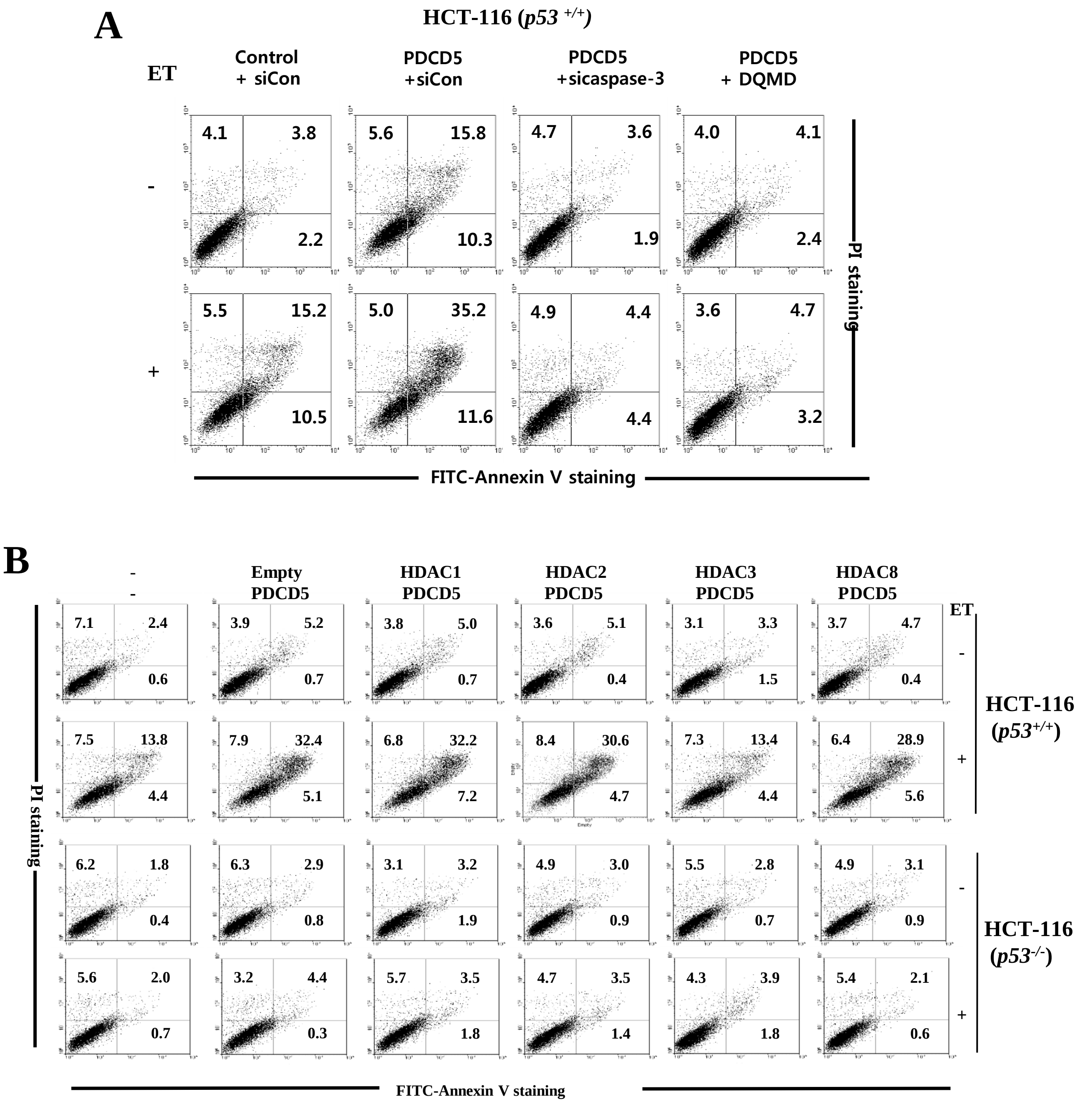

Supplementary figure 12. HDAC3 selectively antagonizes PDCD5-mediated apoptosis. (**A**) Inhibition of caspase-3 blocks the positive action of PDCD5 in ET-induced apoptosis. Cells were transfected with indicated plasmids and treated with ET and/or Z-DQMD. Annexin V-positive cells were assessed by flow cytometry. A figure representative of at least three experiments is shown. (**B**) HDAC3 selectively antagonizes PDCD5-mediated apoptosis. Annexin V-positive cells were assessed by flow cytometry.

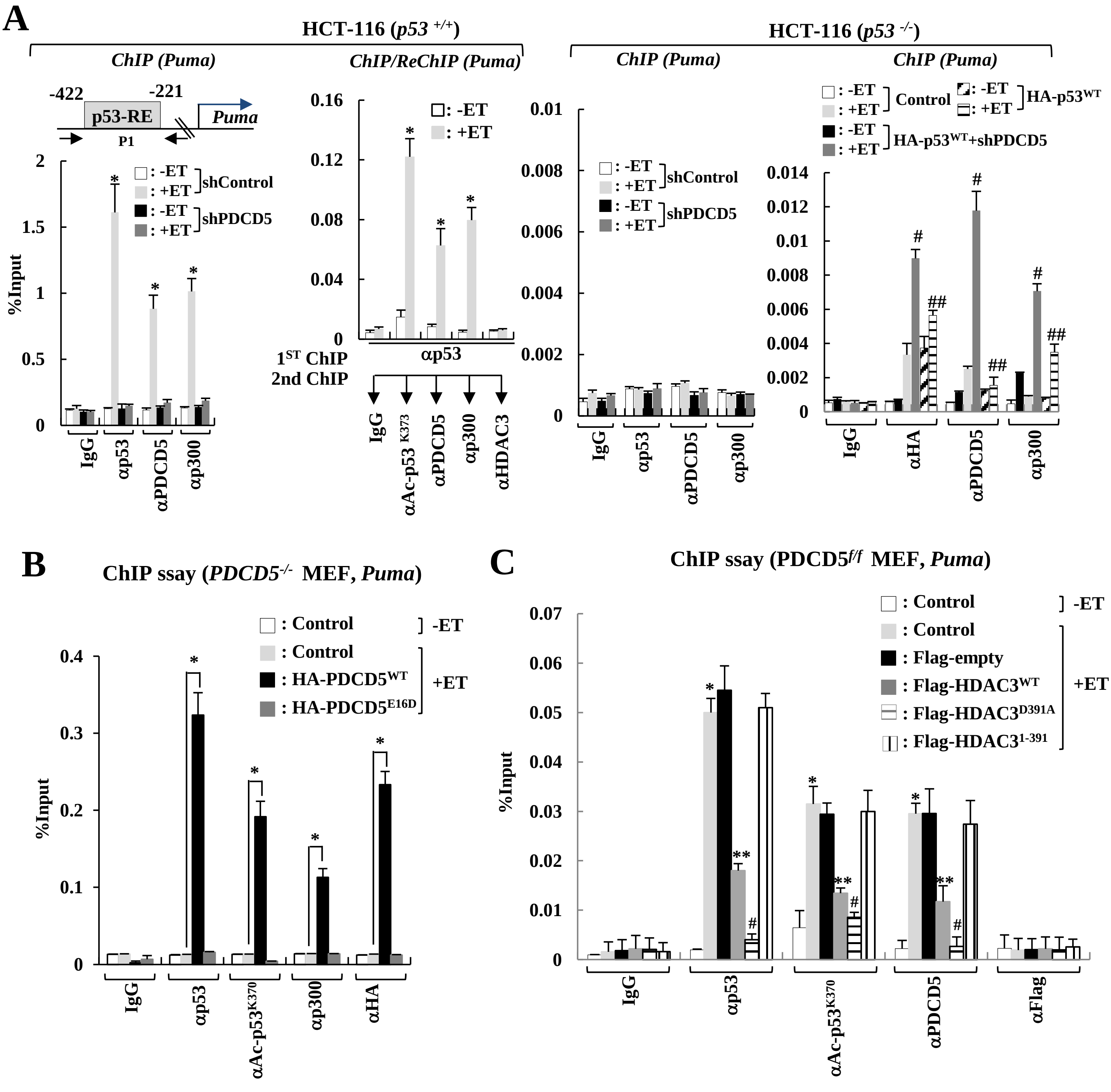

Supplementary figure 13. PDCD5 is required for ET-induced recruitment of the p53-p300 complex to the promoter region of *Puma*. (**A**) HCT-116 cells were transfected with indicated plasmids and/or shPDCD5, and then treated with ET. ChIP and reChIP assays were performed with the indicated antibodies. Precipitated samples were analyzed by real-time PCR, and results are presented as the percentage of input. Error bars, SD (n=3). (*P<0.05 vs. without ET; $^{\#}P$<0.05 vs. without ET; $^{\#\#}P$<0.05 vs. ET+HA-$p53^{WT}$). (**B**) PDCD5-/- MEFs were electroporated with indicated plasmids, treated with ET. ChIP assays were performed with the indicated antibodies. Precipitated samples were analyzed by real-time PCR, and results are presented as the percentage of input. Error bars, SD (n=3). *P<0.05. (**C**) Uncleaved $HDAC3^{D391A}$ mutant more efficiently inhibits the binding of p53 and PDCD5 to Bax promoter when compared with wild-type HDAC3 and cleaved HDAC3. $PDCD5^{f/f}$ MEFs were electroporated with indicated plasmids, treated with ET. ChIP assays were performed with the indicated antibodies. Error bars, SD (n=3). (*P<0.05 vs. without ET; $^{\#}$ P<0.05 vs. ET+Flag-empty; $^{\#\#}P$<0.05 vs. ET+Flag-$HDAC3^{WT}$). SD, Standard deviation.

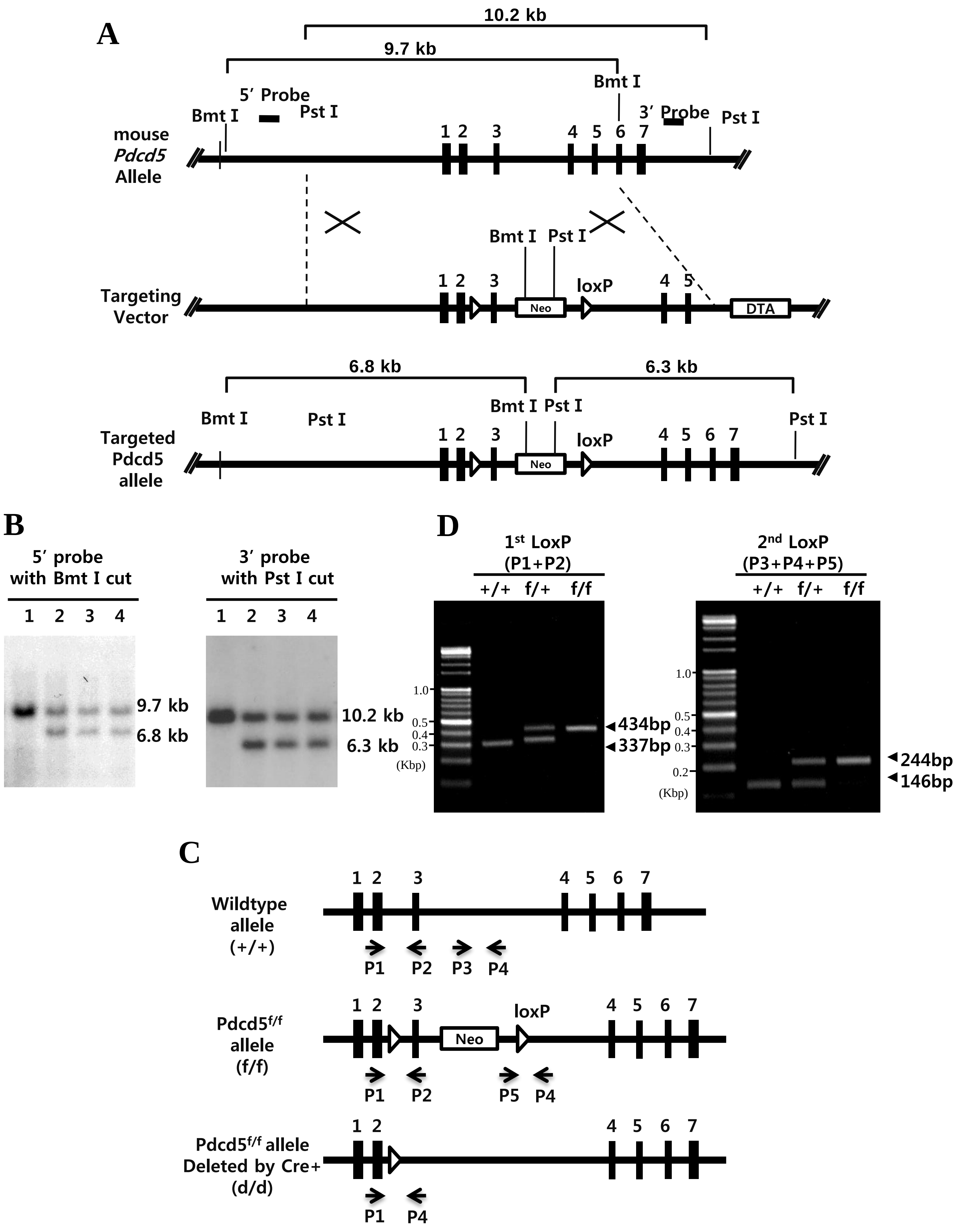
A
10.2 kb
9.7 kb
Bmt I
5′ Probe
Pst I
Bmt I
3′ Probe
Pst I
mouse
Pdcd5
Allele
1 2 3 4 5 6 7
Bmt I
Pst I
Targeting
Vector
1 2 3
Neo
loxP
4 5
DTA
6.8 kb
6.3 kb
Bmt I
Pst I
Bmt I Pst I
Pst I
Targeted
Pdcd5
allele
1 2 3
Neo
loxP
4 5 6 7
B
5′ probe
with Bmt I cut
1 2 3 4
9.7 kb
6.8 kb
3′ probe
with Pst I cut
1 2 3 4
10.2 kb
6.3 kb
D
1st LoxP
(P1+P2)
+/+ f/+ f/f
1.0
0.5
0.4
0.3
(Kbp)
434bp
337bp
2nd LoxP
(P3+P4+P5)
+/+ f/+ f/f
1.0
0.5
0.4
0.3
0.2
(Kbp)
244bp
146bp
C
Wildtype
allele
(+/+)
1 2 3 4 5 6 7
P1 P2 P3 P4
Pdcd5f/f
allele
(f/f)
1 2 3
Neo
loxP
4 5 6 7
P1 P2 P5 P4
Pdcd5f/f allele
Deleted by Cre+
(d/d)
1 2 4 5 6 7
P1 P4

Supplementary figure 14. Conditional targeting strategy of *PDCD5*. (**A**) Conditional targeting strategy of *pdcd5*. One loxP site is located between exon 2 and exon 3. Another is upstream of exon 4. (**B**) Southern blot analysis. Wild-type allele produced 9.7 kb and 10.2 kb bands using 5' and 3' probes after restriction enzyme digest with Bmt I and Pst I, respectively. Targeted allele produced 6.8 kb and 6.3 kb bands. Line 1, wild-type allele. Lines 2-4, targeted allele. (**C**) Location of primers used for genotyping PDCD5 $^{f/f}$. (**D**) Genotyping of PDCD5 $^{f/f}$. The first loxP site is amplified by primers P1 and P2. The second loxP is detected by primers P3, P4, and P5. Wild-type allele, (+/+); floxed allele, (f/+, f/f); deleted allele by Cre recombinase, (d/d). Expected size for the detection of first loxP is 434 bp, whereas wild-type band is 337 bp. The second loxP site is 244 bp compared to a wild-type band of 146 bp.

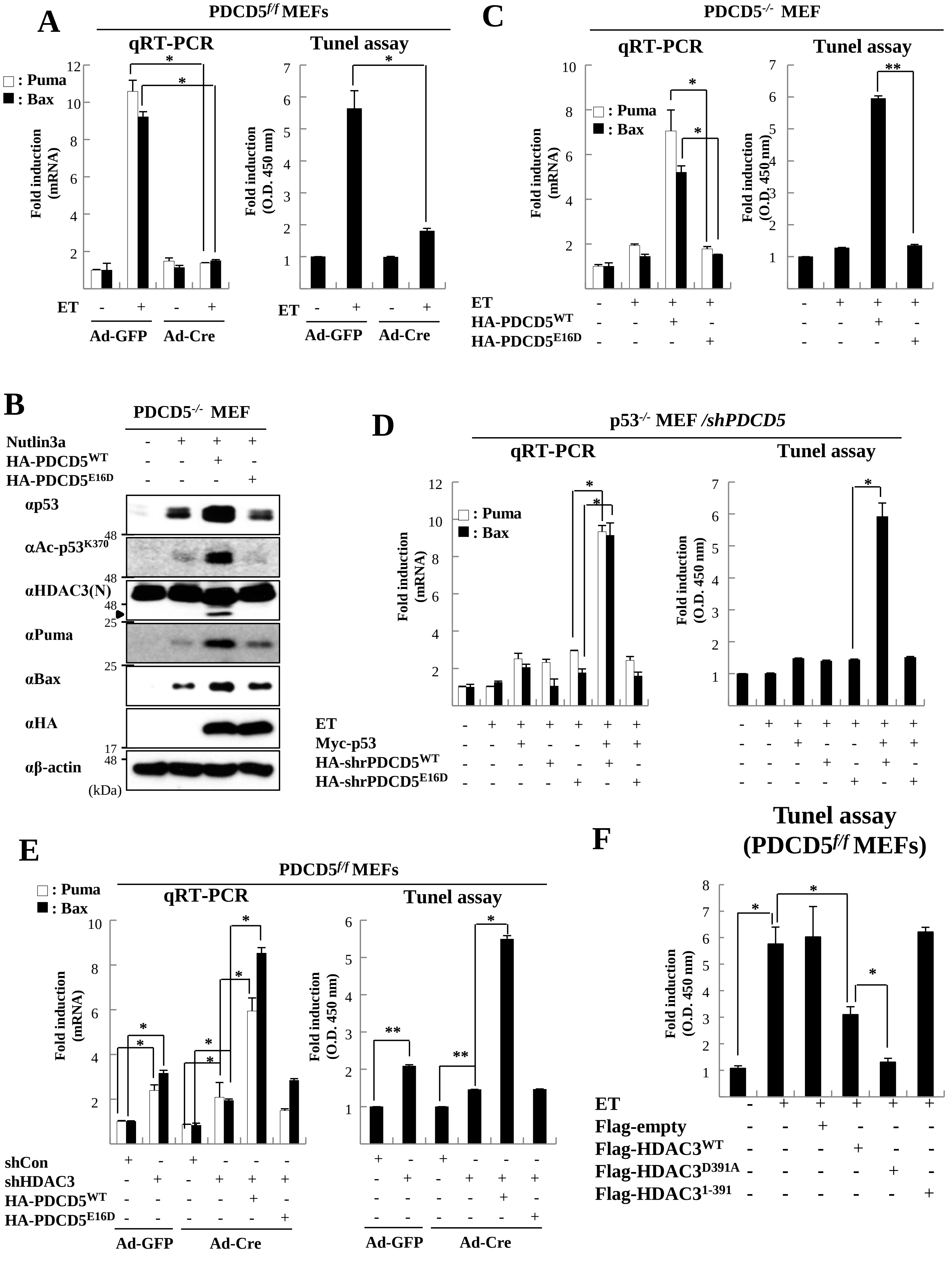

A
PDCD5^f/f MEFs
qRT-PCR
Tunel assay
□ : Puma
■ : Bax
Fold induction (mRNA)
Fold induction (O.D. 450 nm)
ET
Ad-GFP
Ad-Cre
C
PDCD5-/- MEF
HA-PDCD5WT
HA-PDCD5E16D
B
Nutlin3a
αp53
αAc-p53K370
αHDAC3(N)
αPuma
αBax
αHA
αβ-actin
(kDa)
D
p53-/- MEF /shPDCD5
Myc-p53
HA-shrPDCD5WT
HA-shrPDCD5E16D
E
shCon
shHDAC3
F
Tunel assay (PDCD5^f/f MEFs)
Flag-empty
Flag-HDAC3WT
Flag-HDAC3D391A
Flag-HDAC31-391

Supplementary figure 15. **PDCD5 is required for genotoxic stress-induced HDAC3 cleavage and p53 activation.** (**A**) MEFs were infected with Ad-Cre or Ad-GFP and then treated with ET (50 μM, 8 h). Total RNA was isolated from individual MEFs, and qRT-PCR was performed for the indicated genes. Error bars, SD (n=3). **P*<0.05. DNA damage of cells was determined by TUNEL assay. Error bars, SD (n=3). **P*<0.05. (**B**) PDCD5$^{-/-}$ MEFs were electroporated with indicated plasmids, treated with nutlin3a, lysed and then analyzed by immunoblotting. (**C**) MEFs were electroporated with indicated plasmids, and then treated with ET. Total RNA was isolated from individual MEFs, and qRT-PCR was performed for the indicated genes. Error bars, SD (n=3). **P*<0.05. DNA damage of cells was determined by TUNEL assay. Error bars, SD (n=3). ***P*<0.01. (**D**) Stable shPDCD5-expressing p53$^{-/-}$ MEFs were electroporated with indicated plasmids and treated with ET. Total RNA was isolated from individual MEFs, and RT-qPCR was performed for the indicated genes. Error bars, SD (n=3). **P*<0.05. DNA damage of cells was determined by TUNEL assay. Error bars, SD (n=3). **P*<0.05. (**E**) PDCD5$^{f/f}$ MEFs were electroporated with indicated plasmids and/or shRNAs. Total RNA was isolated from individual MEFs, and qRT-PCR was performed for the indicated genes. Error bars, SD (n=3). **P*<0.05. DNA damage of cells was determined by TUNEL assay. Error bars, SD (n=3). **P*<0.05, ***P*<0.01. (**F**) DNA damage of cells was determined by TUNEL assay. Error bars, SD (n=3). **P*<0.05. SD, Standard deviation.

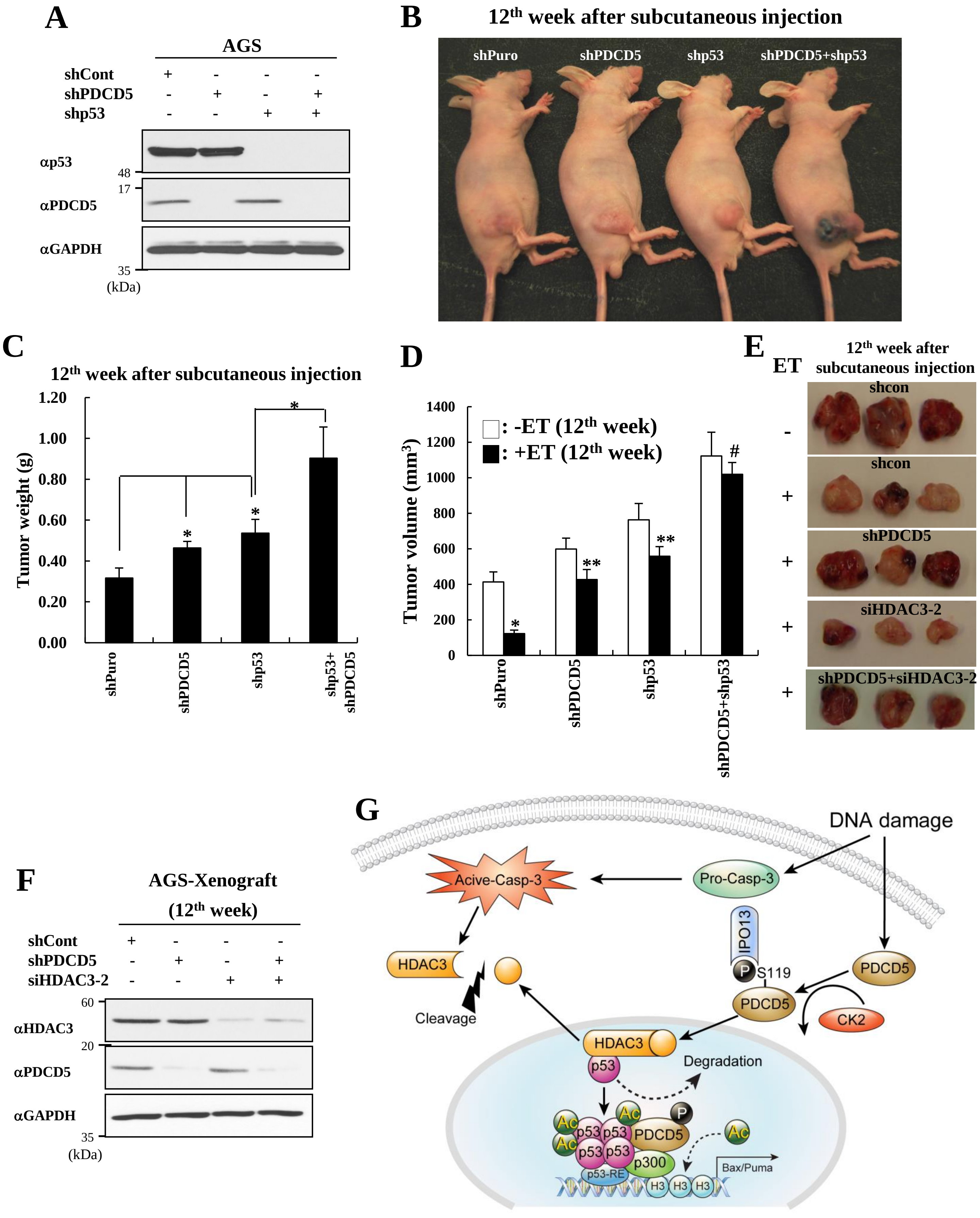
A
AGS
shCont + - - -
shPDCD5 - + - +
shp53 - - + +
αp53
48
17
αPDCD5
αGAPDH
35
(kDa)
B
12th week after subcutaneous injection
shPuro
shPDCD5
shp53
shPDCD5+shp53
C
12th week after subcutaneous injection
Tumor weight (g)
1.20
1.00
0.80
0.60
0.40
0.20
0.00
shPuro
shPDCD5
shp53
shp53+ shPDCD5
D
Tumor volume (mm3)
: -ET (12th week)
: +ET (12th week)
1400
1200
1000
800
600
400
200
0
shPuro
shPDCD5
shp53
shPDCD5+shp53
E
ET
12th week after subcutaneous injection
shcon
shcon
shPDCD5
siHDAC3-2
shPDCD5+siHDAC3-2
F
AGS-Xenograft
(12th week)
shCont + - - -
shPDCD5 - + - +
siHDAC3-2 - - + +
60
αHDAC3
20
αPDCD5
αGAPDH
35
(kDa)
G
DNA damage
Acive-Casp-3
Pro-Casp-3
IPO13
HDAC3
S119
PDCD5
PDCD5
Cleavage
CK2
HDAC3
p53
Degradation
PDCD5
p300
p53-RE
Bax/Puma
H3

Supplementary figure 16. Reduction of PDCD5 and p53 significantly increases the tumor growth and reduces the chemosensitivity of AGS cells compared to reduction of either protein individually. (**A**) AGS cells stably expressing shPDCD5 and/or shp53 were generated as described in the Materials and methods section. Cell lysates were subsequently analyzed by immunoblotting with indicated antibodies. (**B-C**) Stable AGS cells were injected subcutaneously into the right flank of nude mice. At the twelfth week, tumors were harvested and photographed (**B**). Tumor weights were measured at the twelfth week (**C**). Error bars indicate SD (n=6). *$P<0.05$. (**D**) Stable AGS cells were injected subcutaneously into the right flank of nude mice. Four weeks after injection, mice with comparable-sized tumors (100~200 mm^3) were selected for treatment with etoposide (10 mg/kg), with 2 days interval for eight weeks. At the termination of the study, tumor volumes were measured at the twelfth week. (*$P<0.05$ vs -ET; **$P<0.05$ vs shPuro+ET; $^{\#}P<0.05$ vs shPDCD5+ET.). Error bars indicate SD (n=6). (**E**) Stable shCon or shPDCD5-AGS cells were injected subcutaneously into the right flank of nude mice. Four weeks after injection, mice with comparable-sized tumors (100~200 mm^3) were selected for treatment with etoposide (10 mg/kg), with 2 days interval for eight weeks. For siHDAC3 treatment, HDAC3 siRNA/in vivo jetPEI complexes were prepared in a volume of 20 μl per tumor by using the in vivo jetPEI delivery reagent (20 μg siRNA/20 μl jetPEI) according to the manufacturer's instruction. The mice xenograft reaching a tumor volume of 60~80 mm^3 was injected intratumorally into each animal seven times at 2 days interval. At the termination of the study, tumors were harvested and photographed. (**F**) Mice were sacrificed and tumor tissues were collected, processed, and subjected to immunoblotting with indicated antibodies. (**G**) Model of our findings. Upon genotoxic stress, the Ser-119 of PDCD5 is phosphorylated by CK2α. The phosphorylated PDCD5 is recognized and imported into the nucleus by IPO13. Phosphor-PDCD5 stabilizes p53 by inducing dissociation of HDAC3 from p53, leading to cytosolic cleavage of HDAC3 and ubiquitin-dependent proteosomal degradation in a caspase-3-dependent manner. Subsequently, PDCD5 mediates the recruitment of acetylated p53-p300 complex to the promoter region of p53-target genes for promoting transcription of pro-apoptotic genes and apoptosis. SD, Standard deviation.

Supplementary Figure 17. Original images of the cropped blots in the main Figures

Fig. 1a

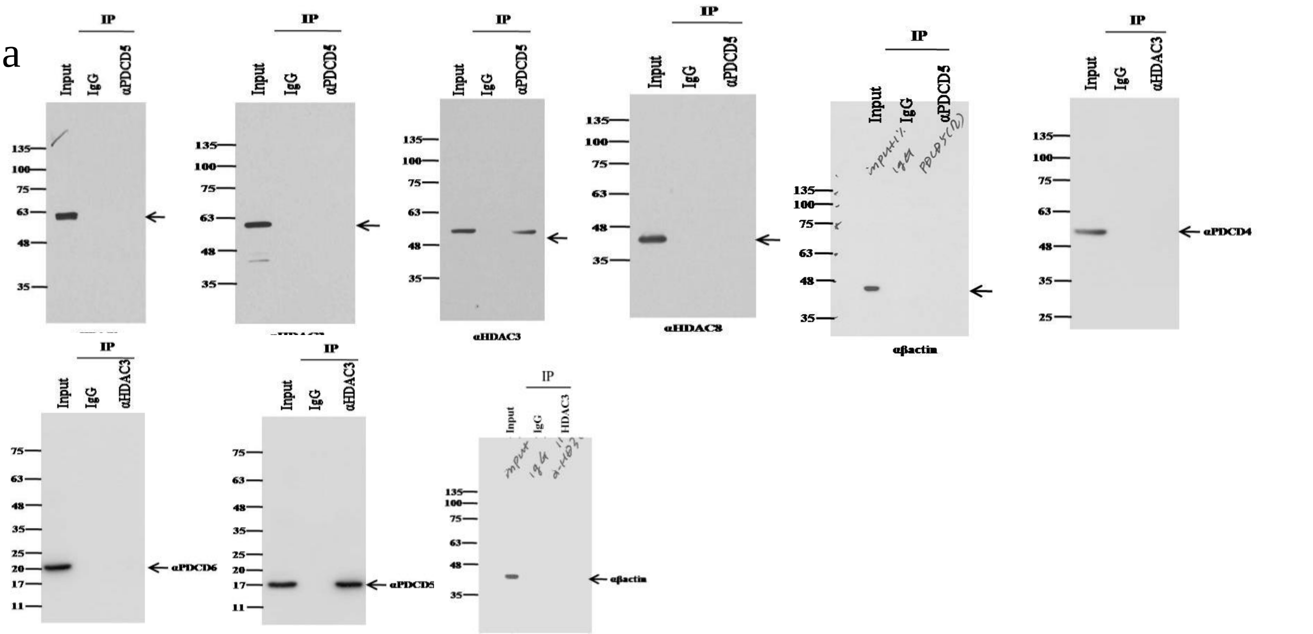

Fig. 1b

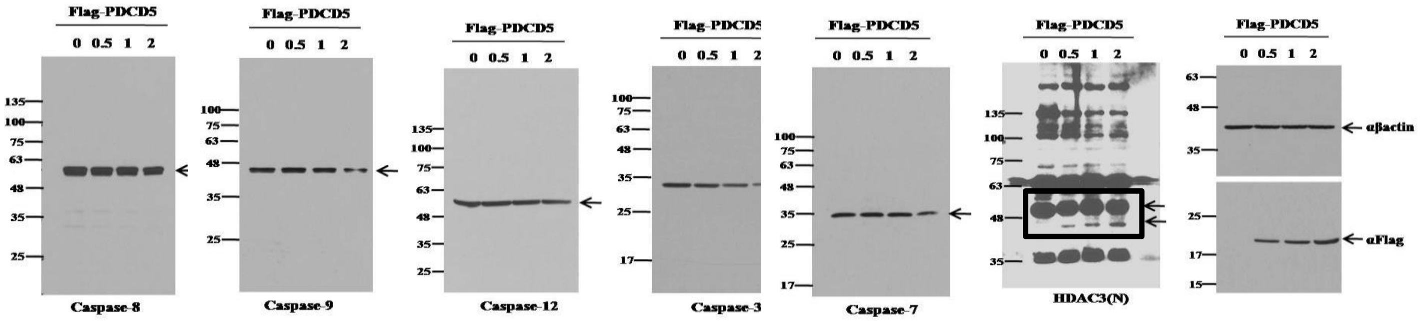

Fig. 1c

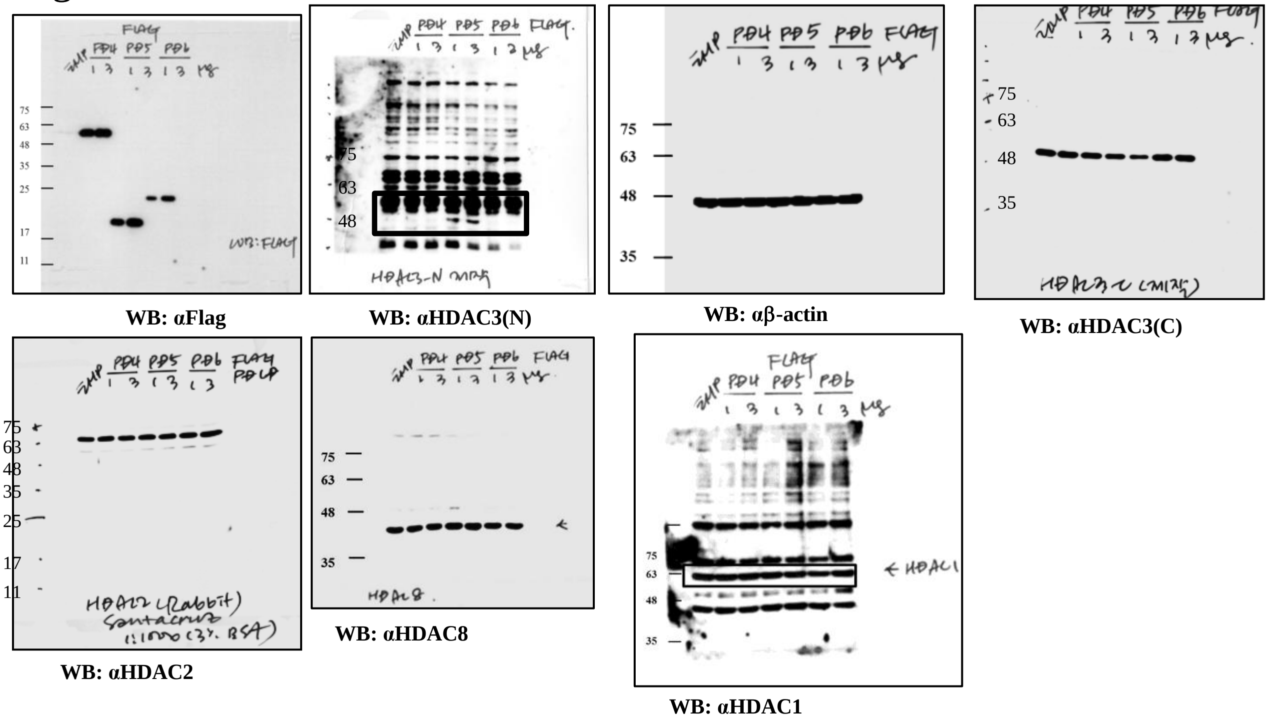

Fig. 1f

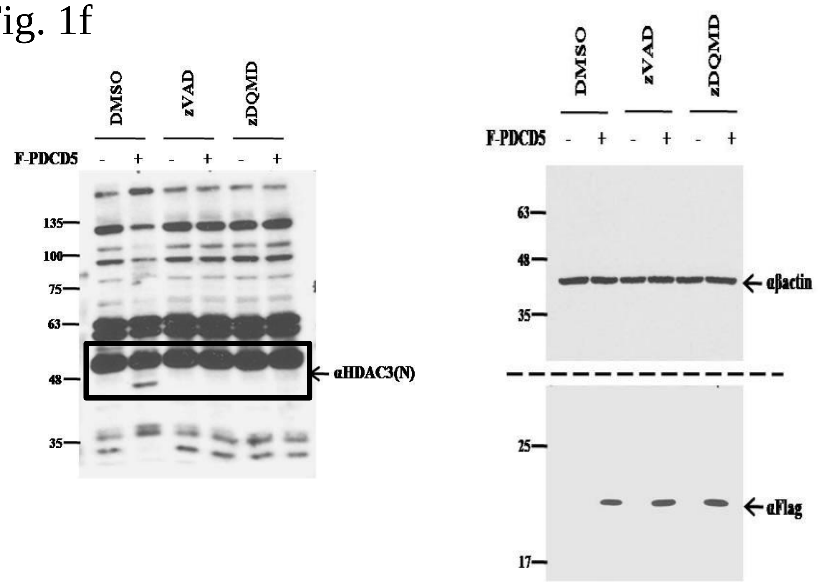

Fig. 1g

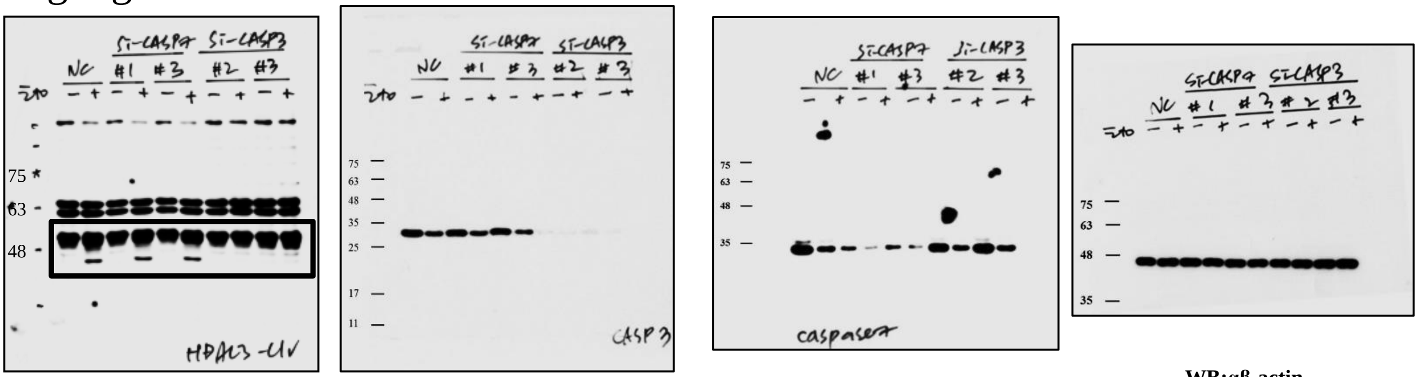

WB: α HDAC3(N)

WB: α Caspase3

WB: α Caspase7

WB:αβ-actin

Fig. 1h

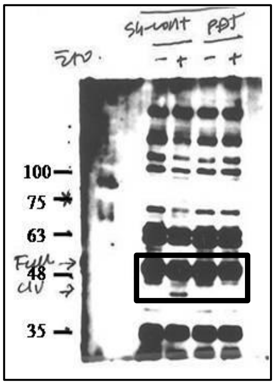

WB: αHDAC3(N)

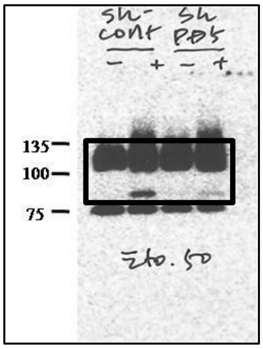

WB: αPARP1

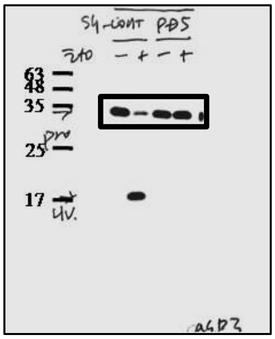

WB: αCaspase3

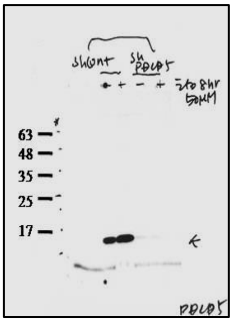

WB: αPDCD5

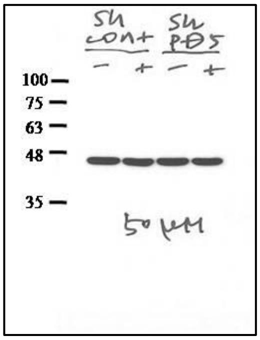

WB:αβ-actin

Fig. 2c

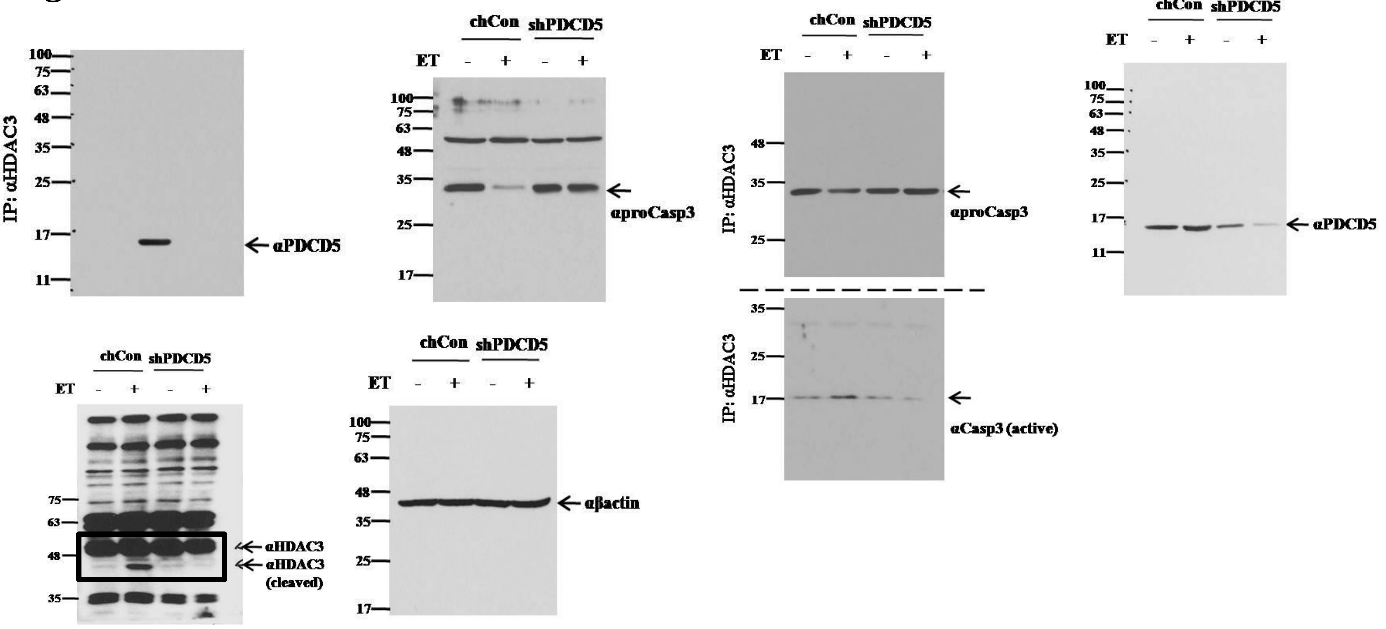
IP: αHDAC3
αPDCD5
chCon
shPDCD5
ET
αproCasp3
αCasp3 (active)
αHDAC3
αHDAC3 (cleaved)
αβactin
100
75
63
48
35
25
17
11

Fig. 2d

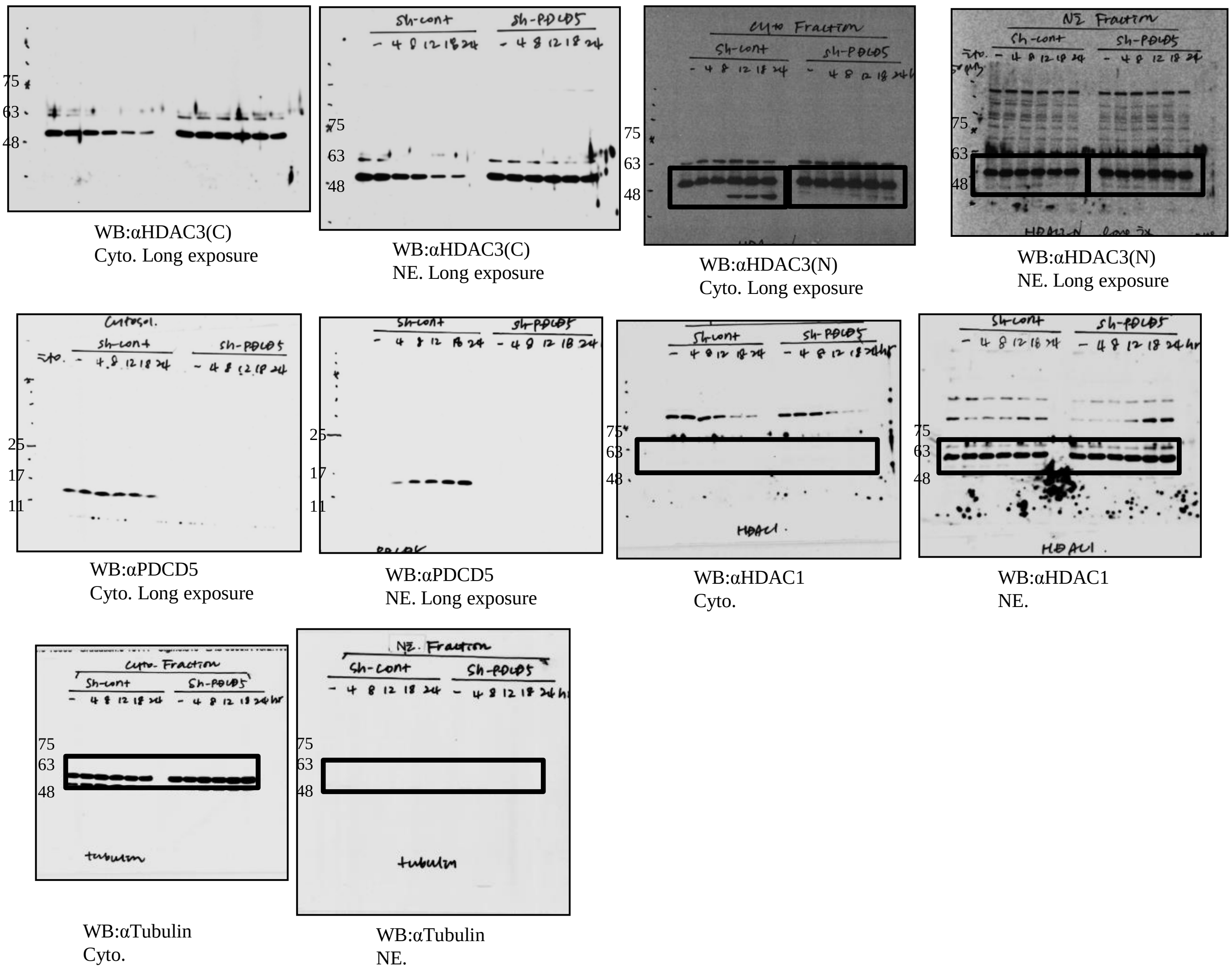
WB:αHDAC3(C)
Cyto. Long exposure
WB:αHDAC3(C)
NE. Long exposure
WB:αHDAC3(N)
Cyto. Long exposure
WB:αHDAC3(N)
NE. Long exposure
WB:αPDCD5
Cyto. Long exposure
WB:αPDCD5
NE. Long exposure
WB:αHDAC1
Cyto.
WB:αHDAC1
NE.
WB:αTubulin
Cyto.
WB:αTubulin
NE.
75
63
48
25
17
11

Fig. 2g

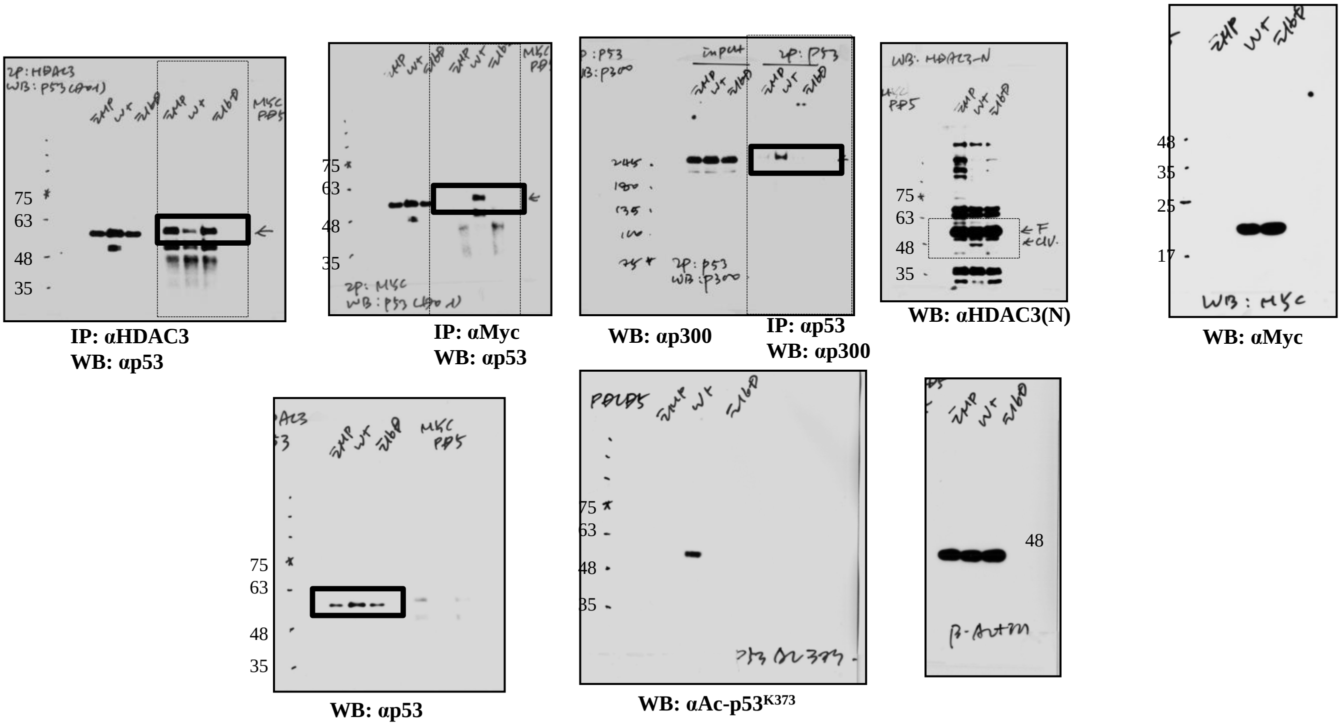
IP: αHDAC3
WB: αp53
IP: αMyc
WB: αp53
WB: αp300
IP: αp53
WB: αp300
WB: αHDAC3(N)
WB: αMyc
WB: αp53
WB: αAc-p53K373
75
63
48
35
25
17

Fig. 2f

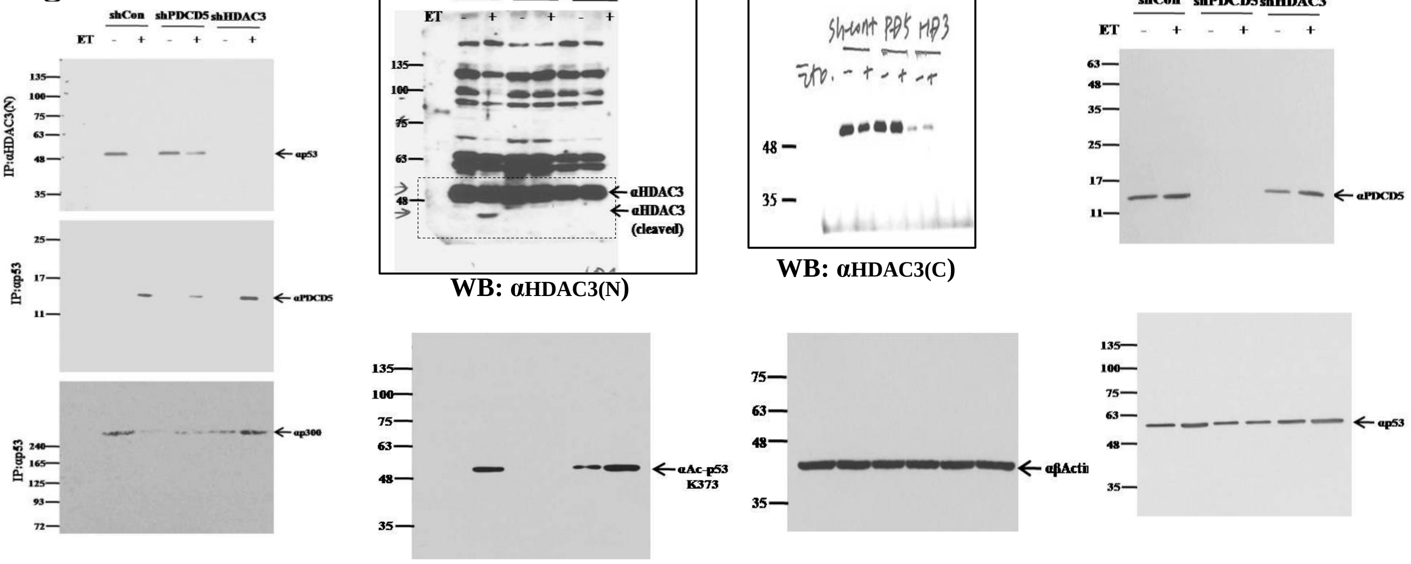
shCon
shPDCD5
shHDAC3
ET
IP:αHDAC3(N)
IP:αp53
αp53
αPDCD5
αp300
αHDAC3
αHDAC3 (cleaved)
WB: αHDAC3(N)
WB: αHDAC3(C)
αAc-p53 K373
αβActi
135
100
75
63
48
35
25
17
11
240
165
125
93
72

Fig. 2h

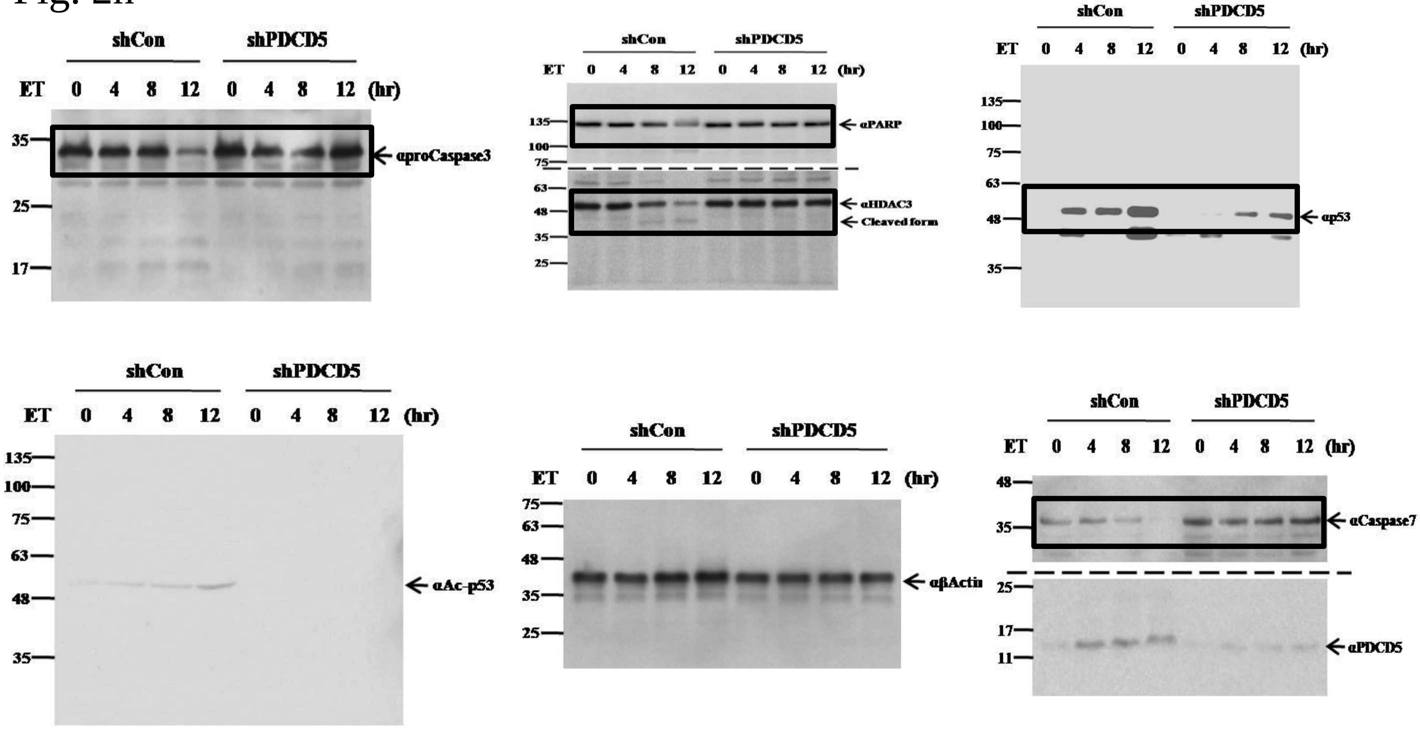

shCon
shPDCD5
ET 0 4 8 12 0 4 8 12 (hr)
αproCaspase3
αPARP
αHDAC3
Cleaved form
αp53
αAc-p53
αβActin
αCaspase7
αPDCD5

Fig. 2i

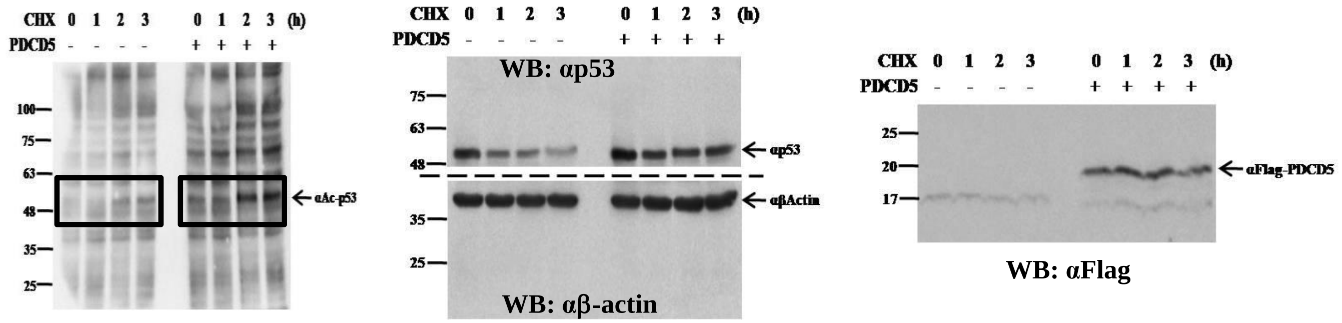

CHX 0 1 2 3 0 1 2 3 (h)
PDCD5 - - - - + + + +
αAc-p53
WB: αp53
αp53
αβActin
WB: αβ-actin
αFlag-PDCD5
WB: αFlag

Fig. 3a

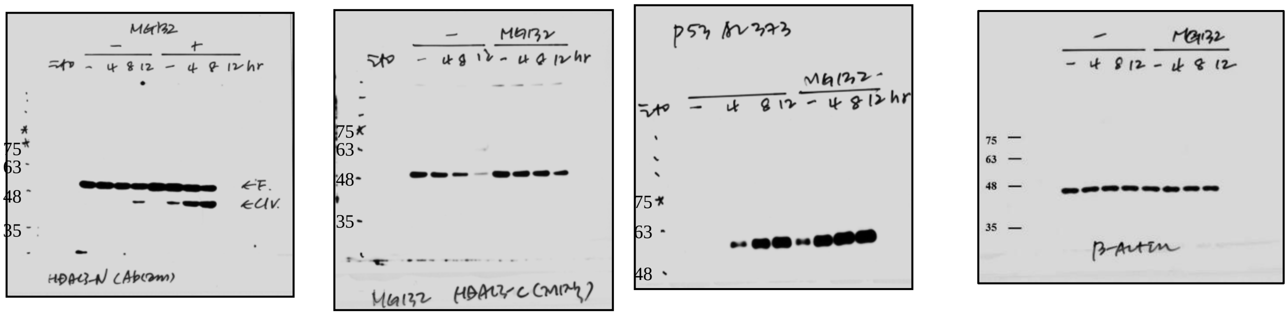

Fig. 3b

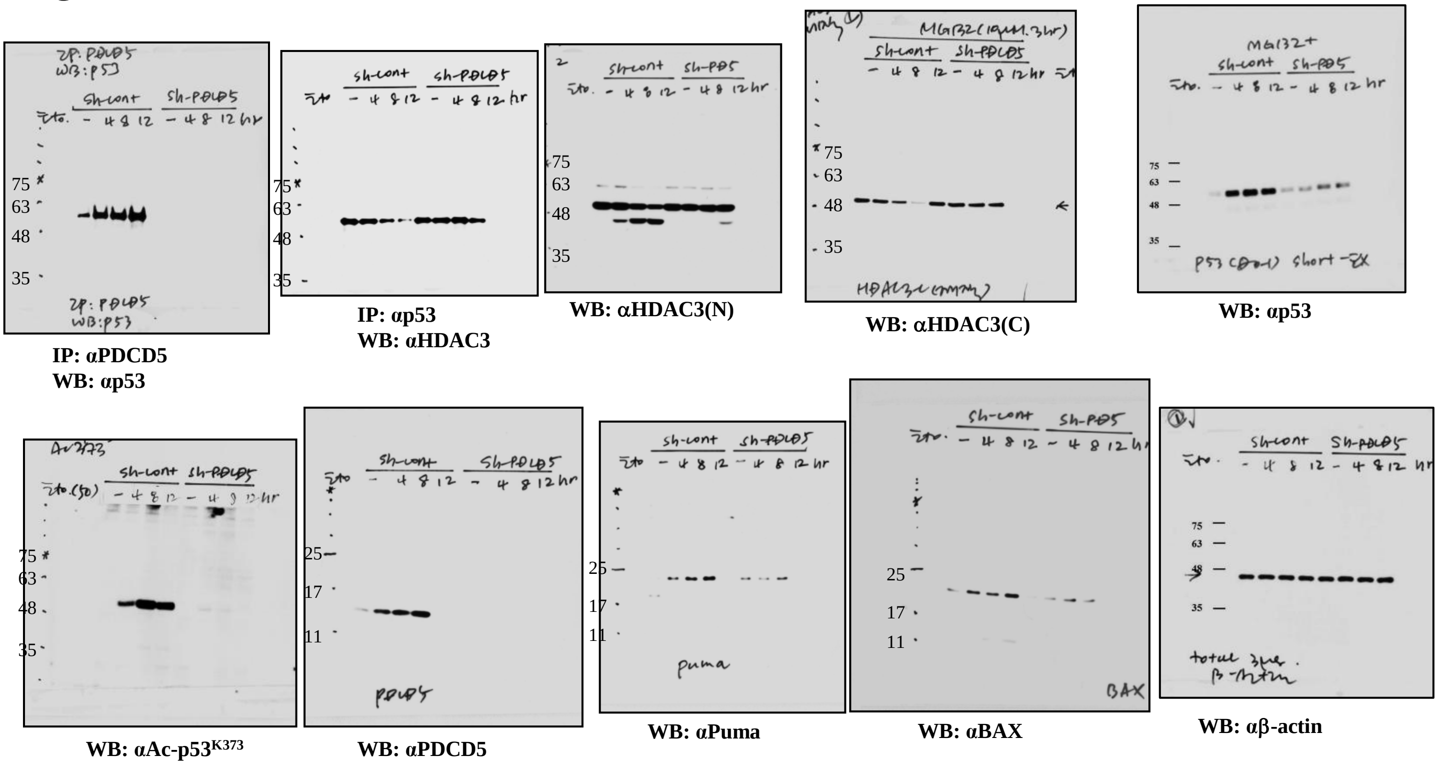

Fig. 3c

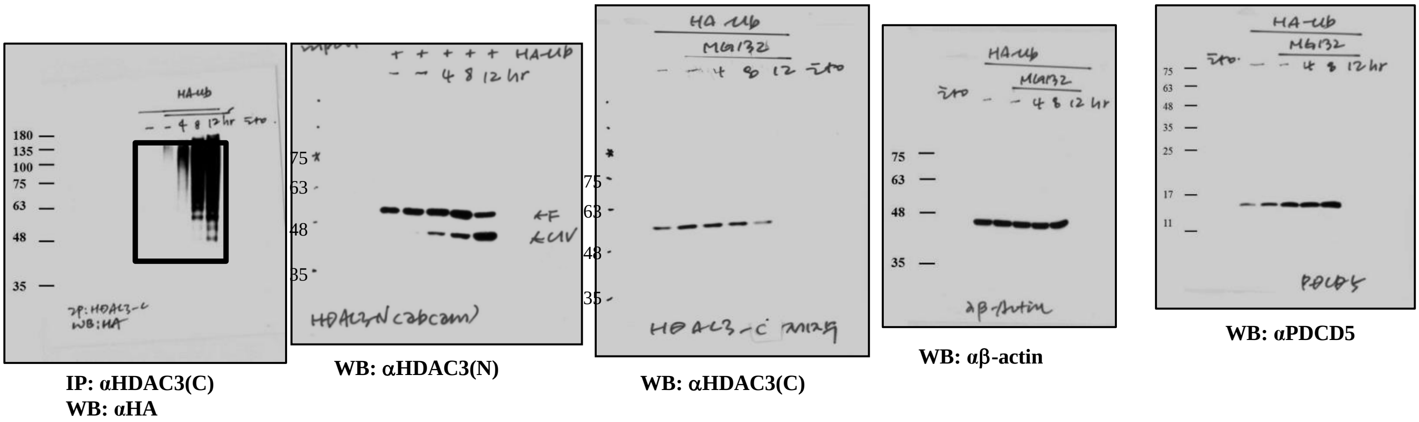

Fig. 3d

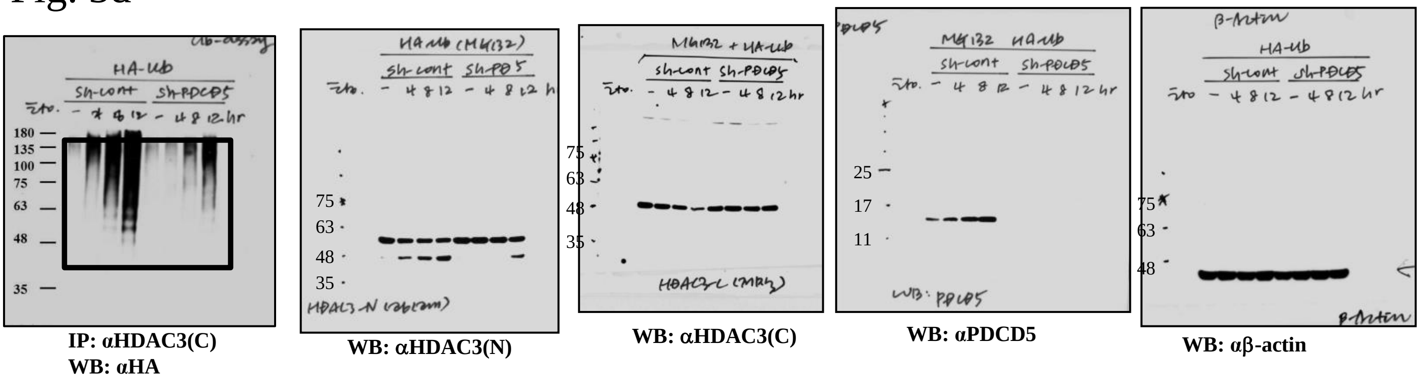

Fig. 3e

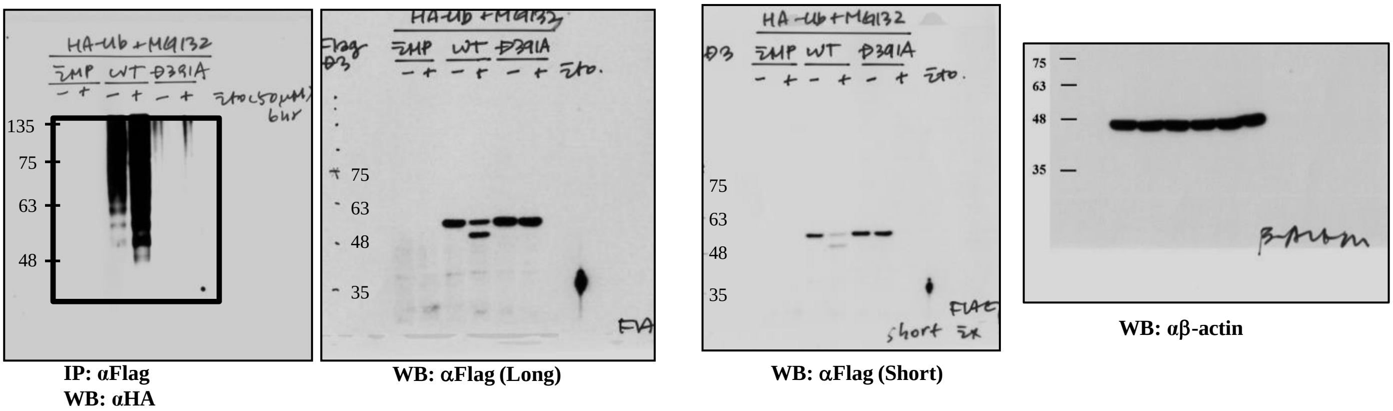

Fig. 3f

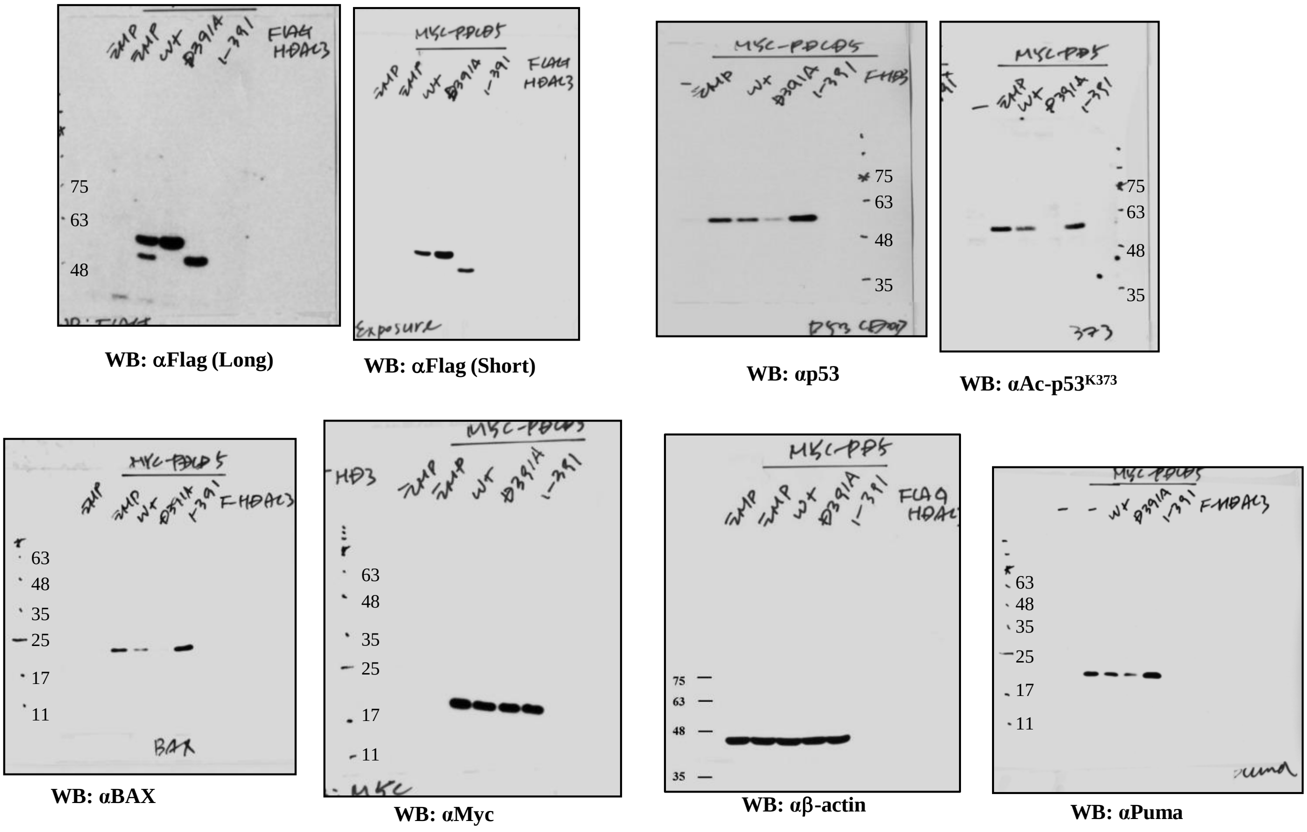

Fig. 4a

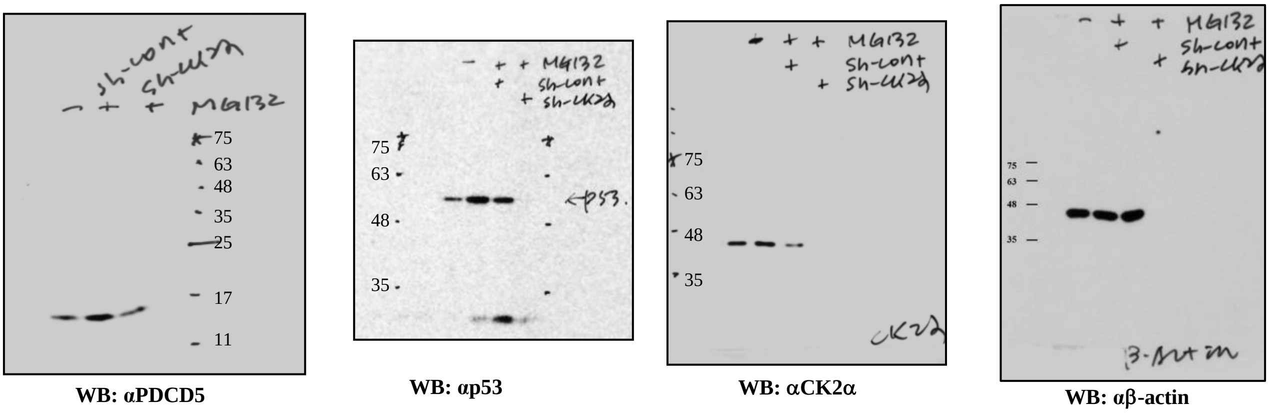

Fig. 4b

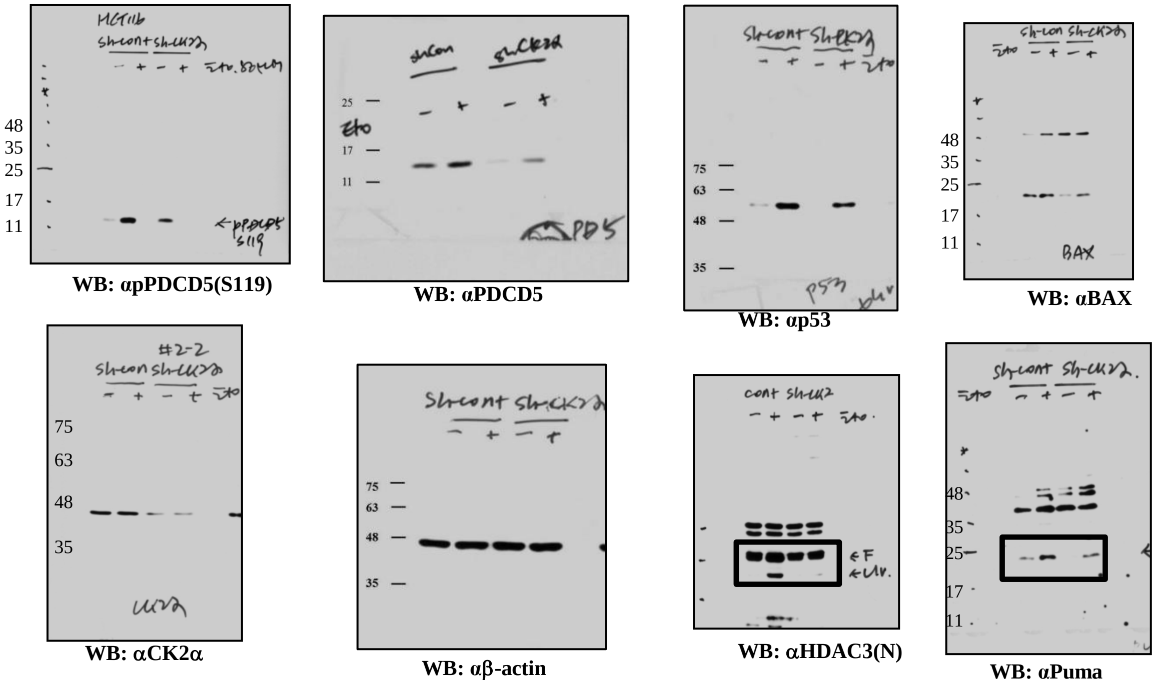

Fig. 4c

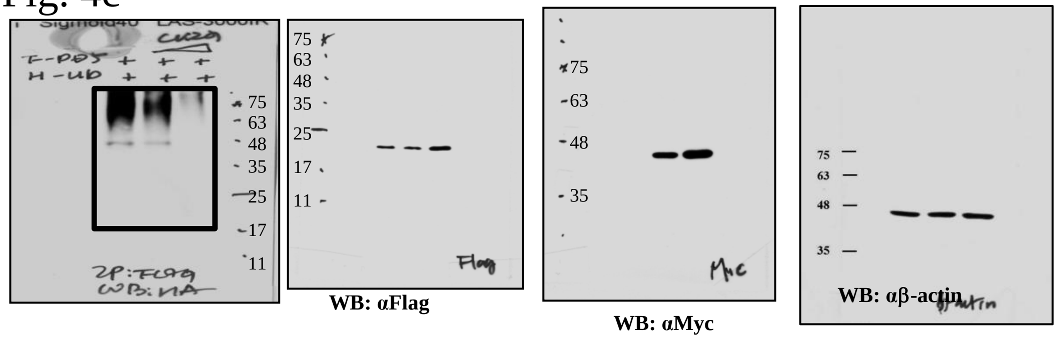

Fig. 4e

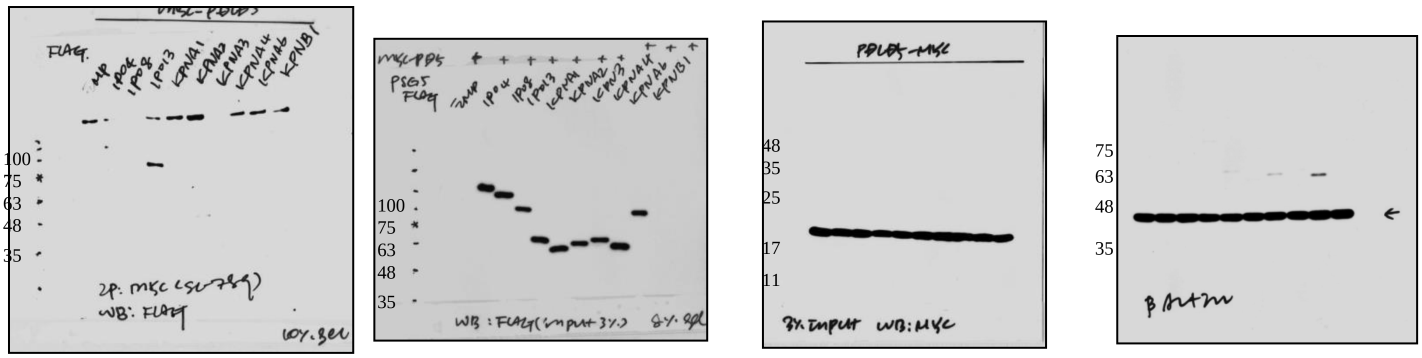
100
75
63
48
35
100
75
63
48
35
48
35
25
17
11
75
63
48
35

Fig. 4h

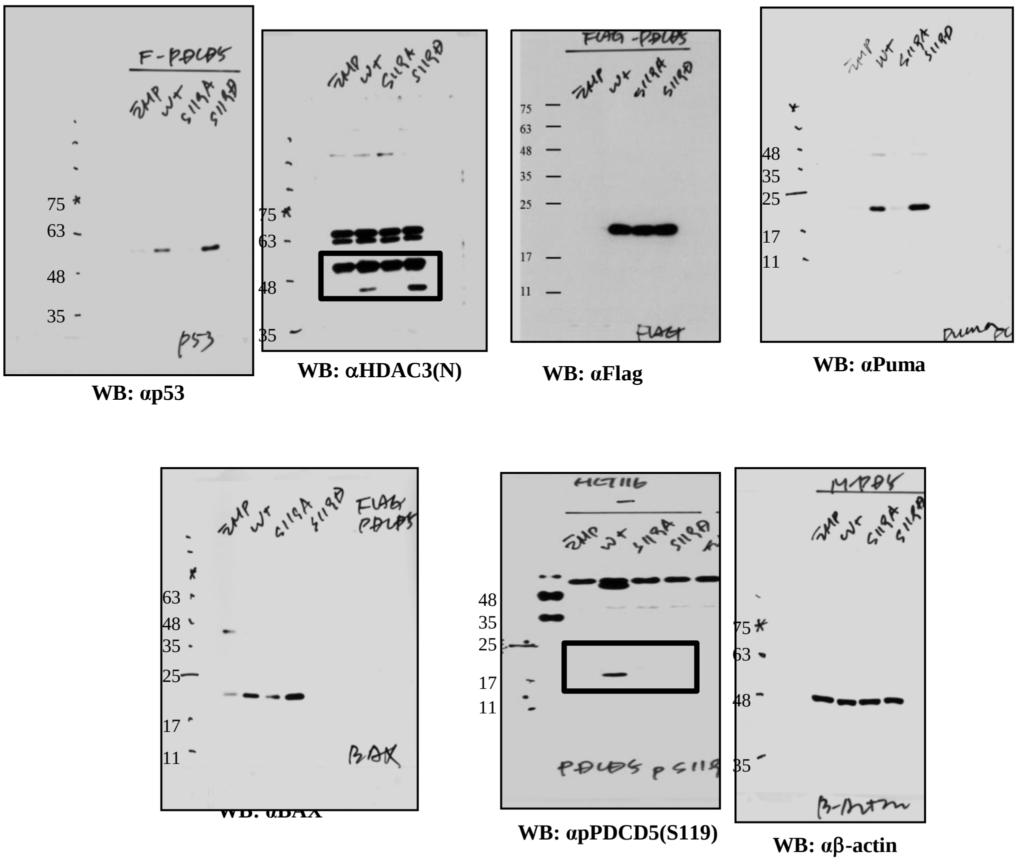
75
63
48
35
WB: αp53
75
63
48
35
WB: αHDAC3(N)
75
63
48
35
25
17
11
WB: αFlag
48
35
25
17
11
WB: αPuma
63
48
35
25
17
11
WB: αBAX
48
35
25
17
11
WB: αpPDCD5(S119)
75
63
48
35
WB: αβ-actin

Fig. 6a

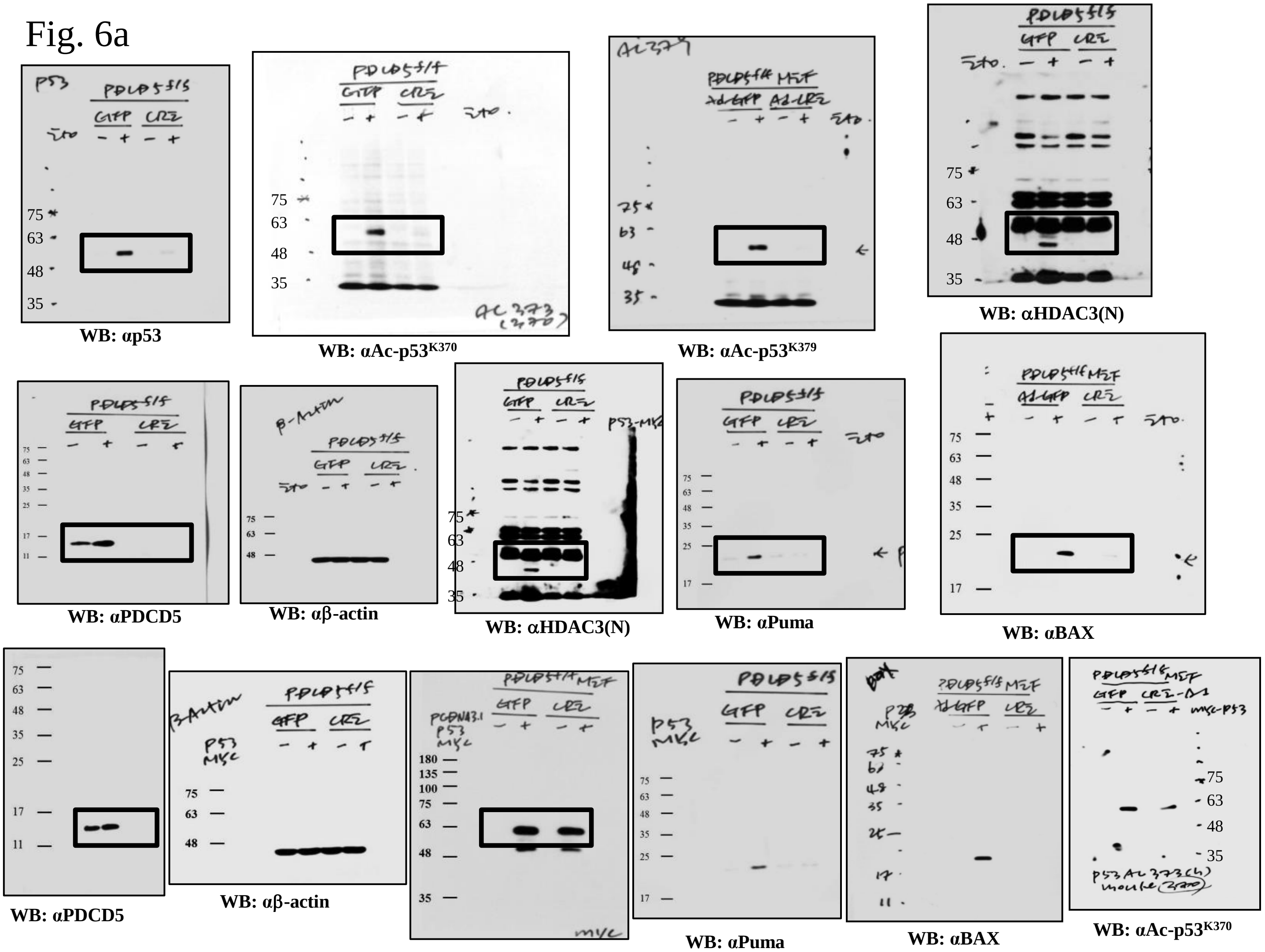

WB: αp53 | WB: αAc-p53^{K370} | WB: αAc-p53^{K379} | WB: αHDAC3(N)

WB: αPDCD5 | WB: αβ-actin | WB: αHDAC3(N) | WB: αPuma | WB: αBAX

WB: αPDCD5 | WB: αβ-actin | WB: αMyc | WB: αPuma | WB: αBAX | WB: αAc-p53^{K370}

Fig. 6b

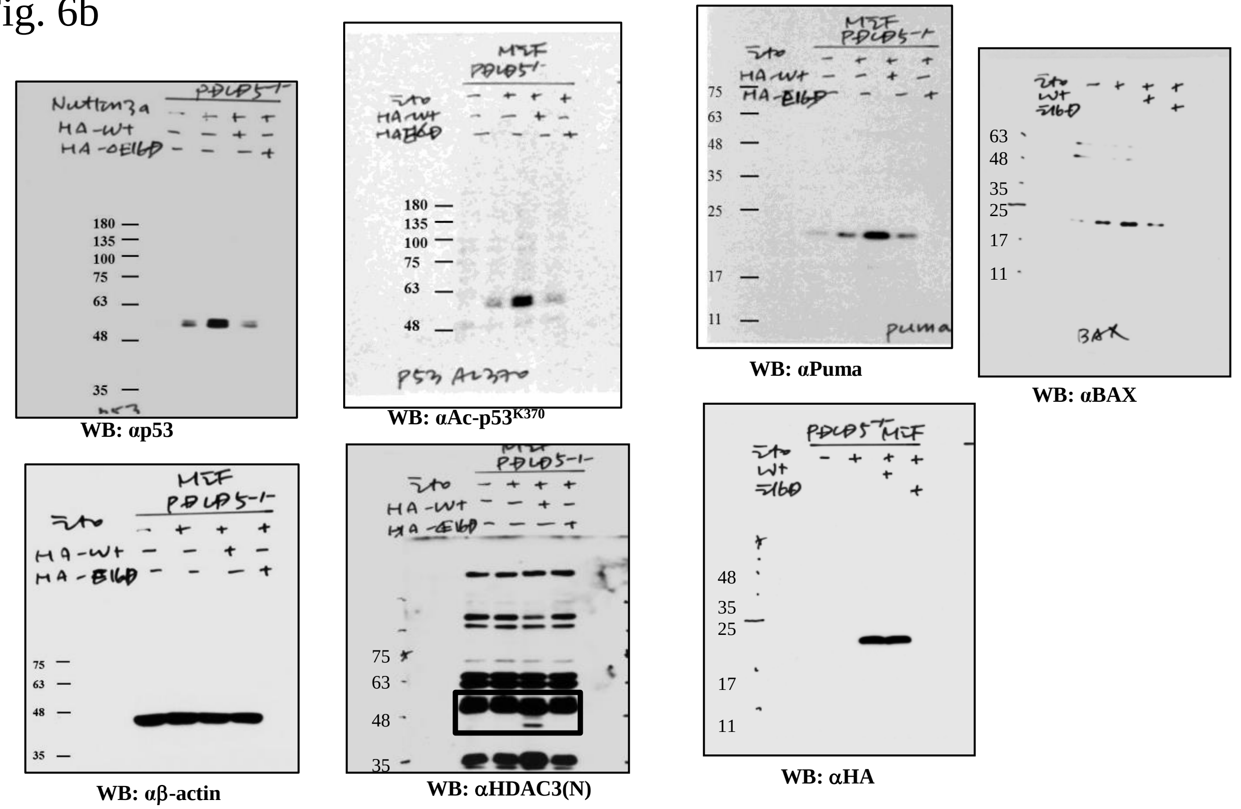

WB: αp53 | WB: αAc-p53^{K370} | WB: αPuma | WB: αBAX

WB: αβ-actin | WB: αHDAC3(N) | WB: αHA

Fig. 6d

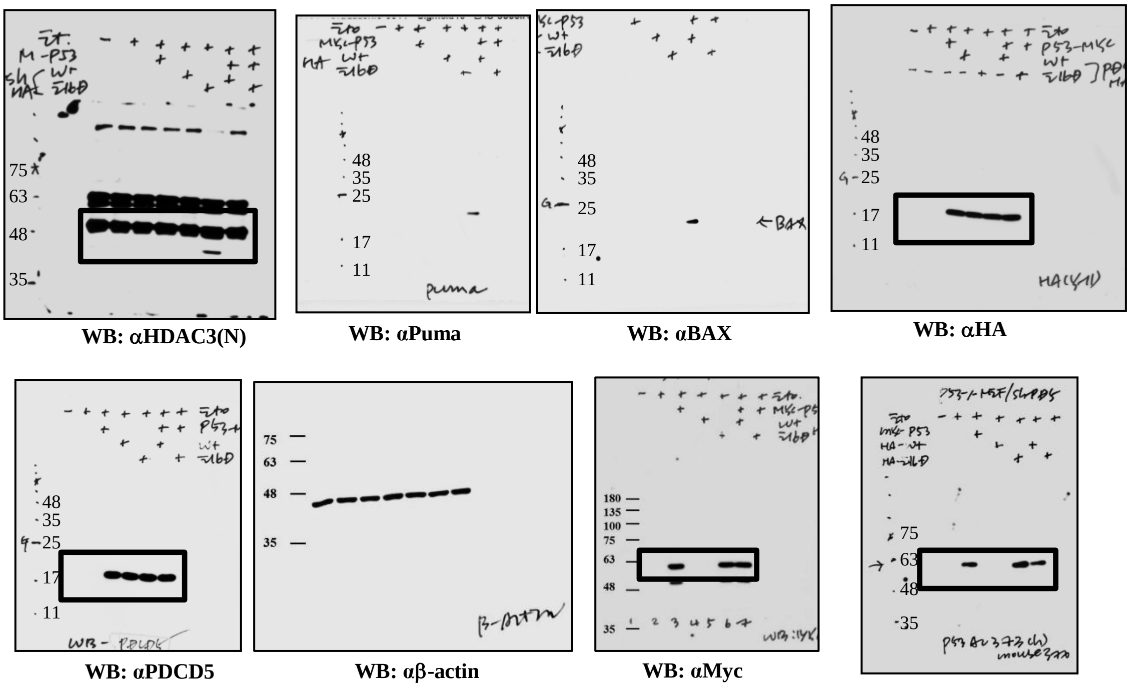

Fig. 6e

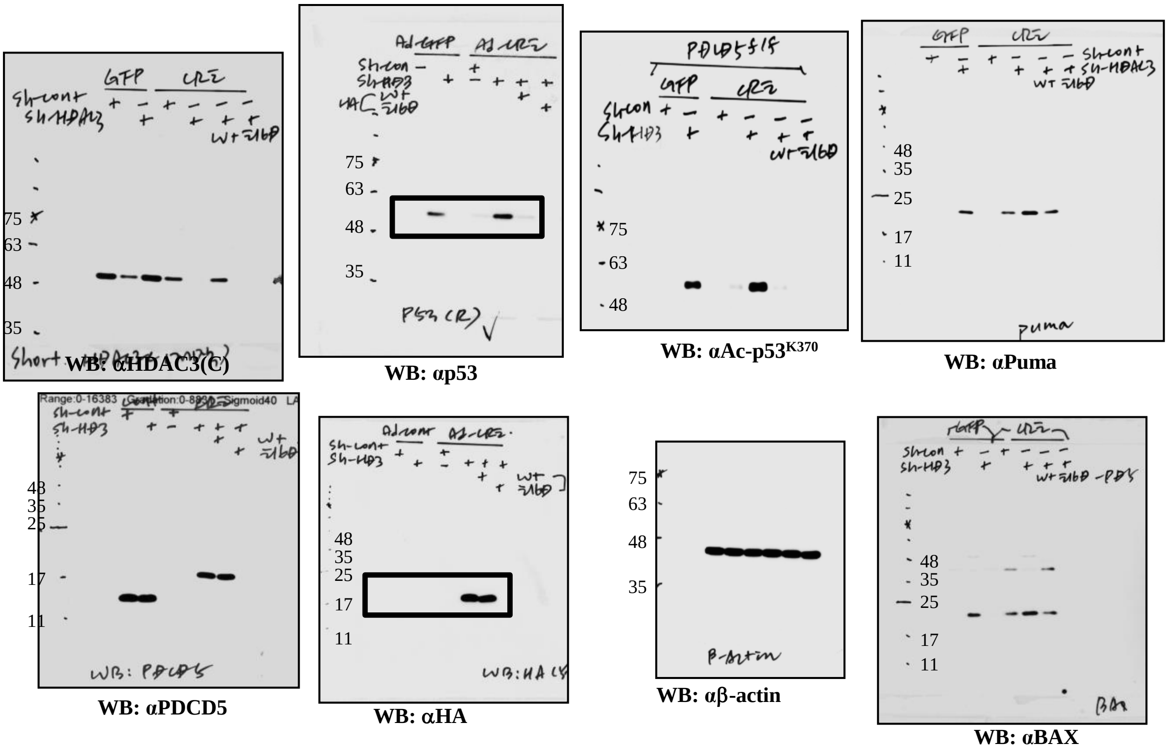

Fig. 6f

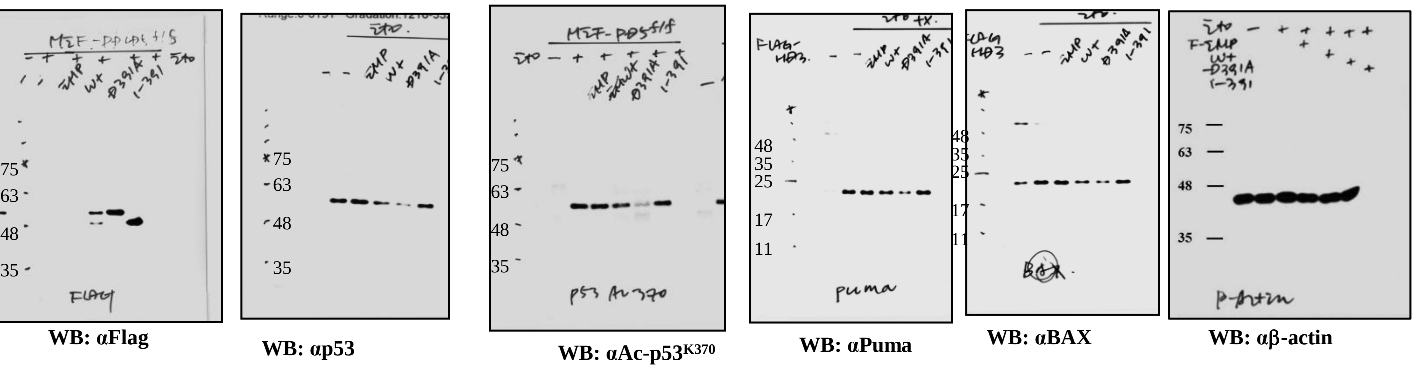

Fig. 6g

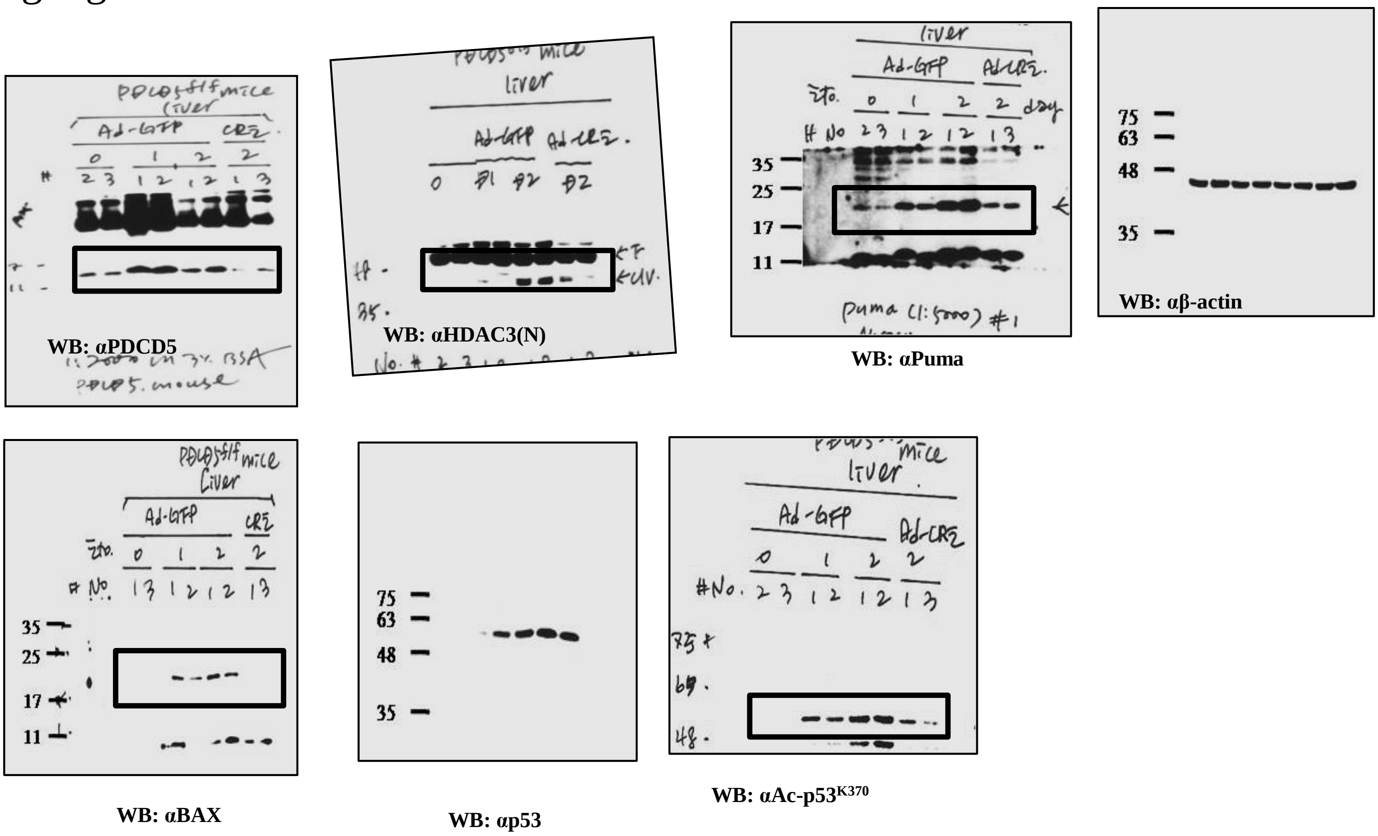
WB: αPDCD5
WB: αHDAC3(N)
WB: αPuma
WB: αβ-actin
WB: αBAX
WB: αp53
WB: αAc-p53K370

Fig. 7b

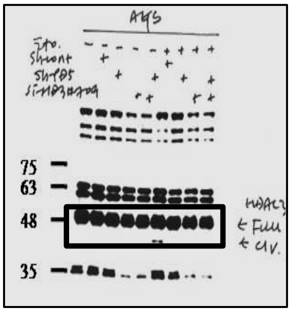

WB: αHDAC3(N)

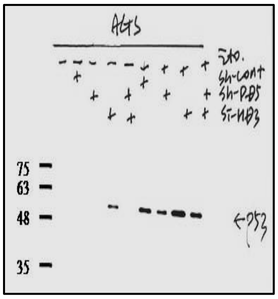

WB: αp53

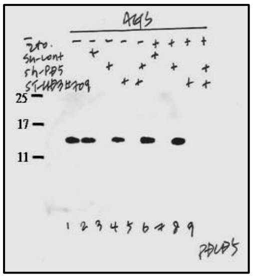

WB: αPDCD5

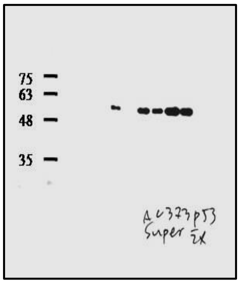

WB: αAc-p53^{K373}

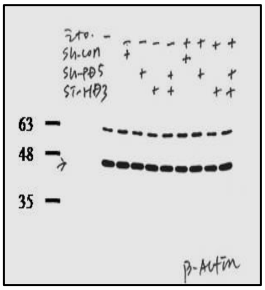

WB: αβ-actin

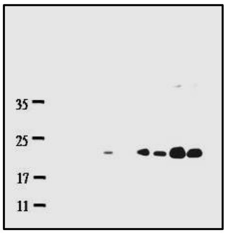

WB: αBax

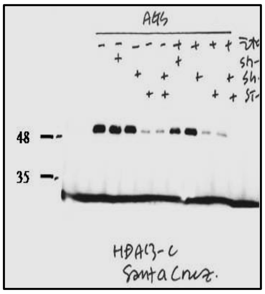

WB: αHDAC3(C)

Fig. 7c

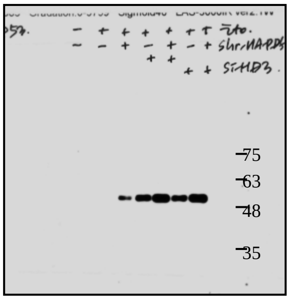

WB: αp53

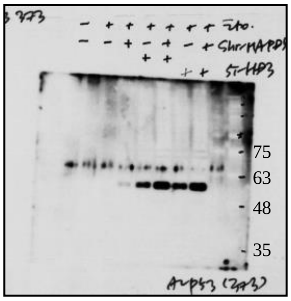

WB: αAc-p53^{K373}

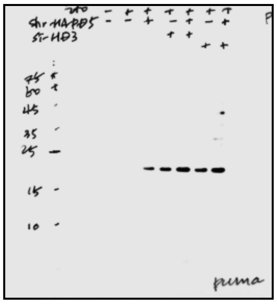

WB: αPuma

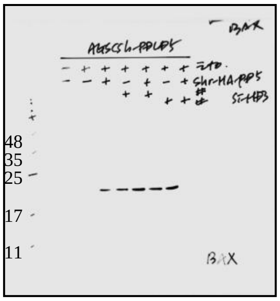

WB: αBAX

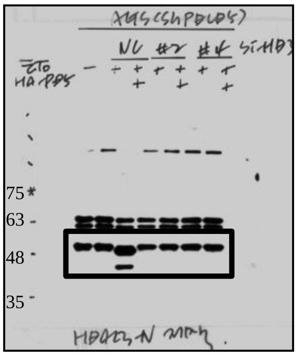

WB: αHDAC3(N)

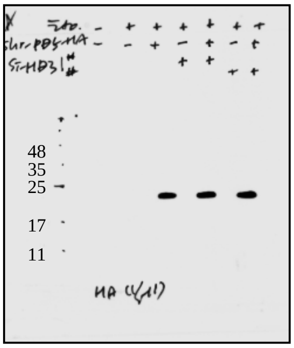

WB: αHA

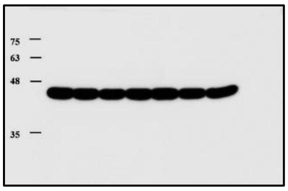

WB: αβ-actin

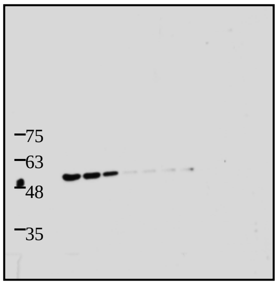

WB: αHDAC3(C)

Suppl. Fig. 1a

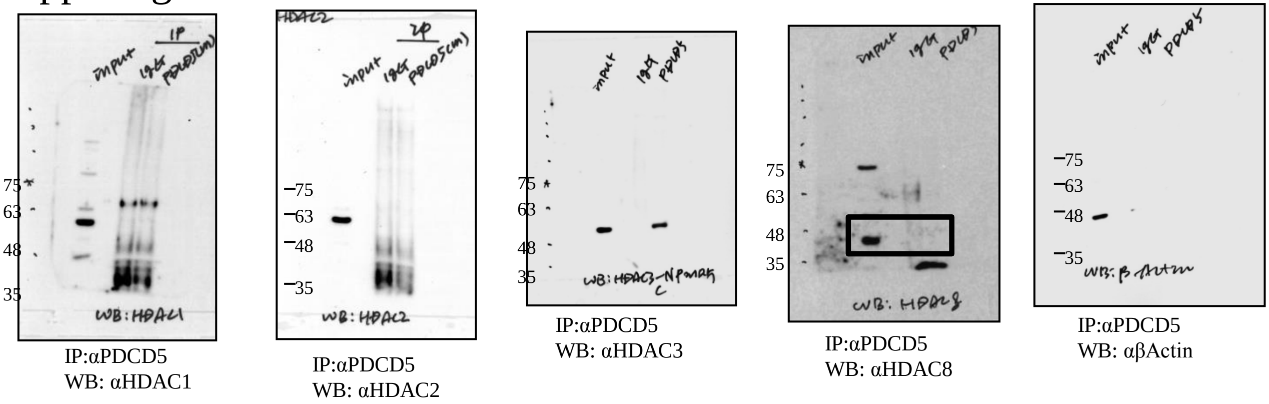

Suppl. Fig. 1b

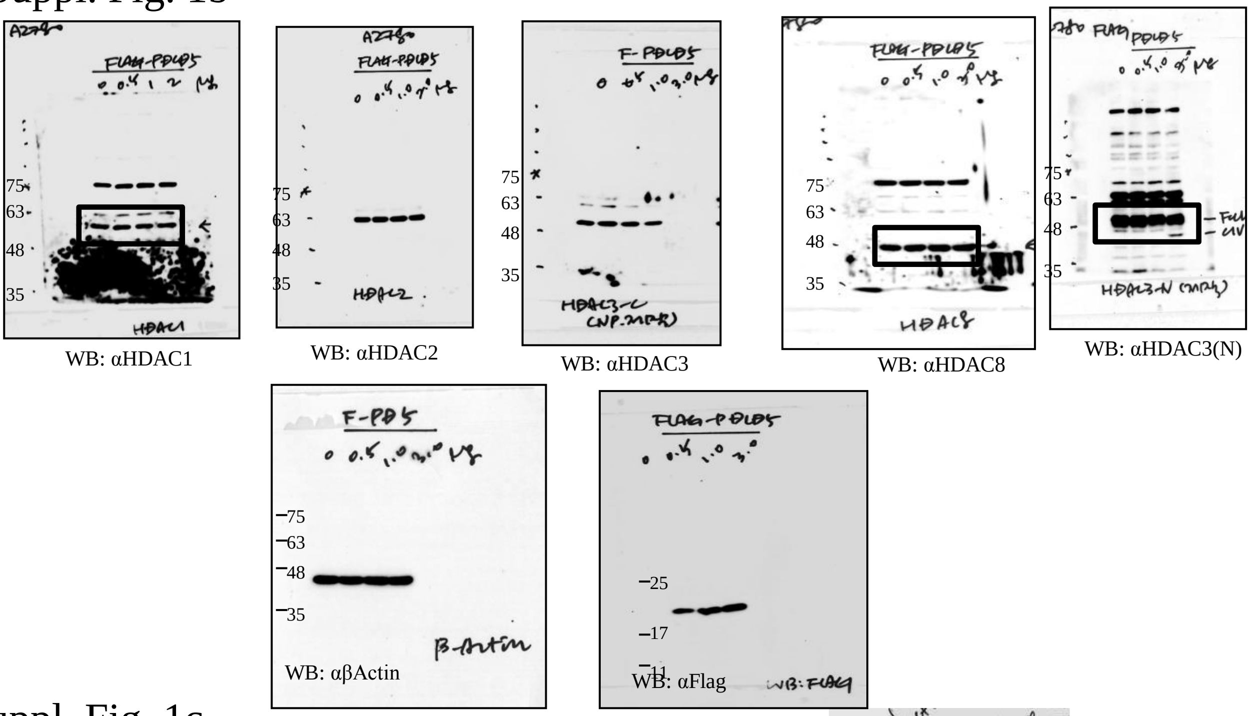

Suppl. Fig. 1c

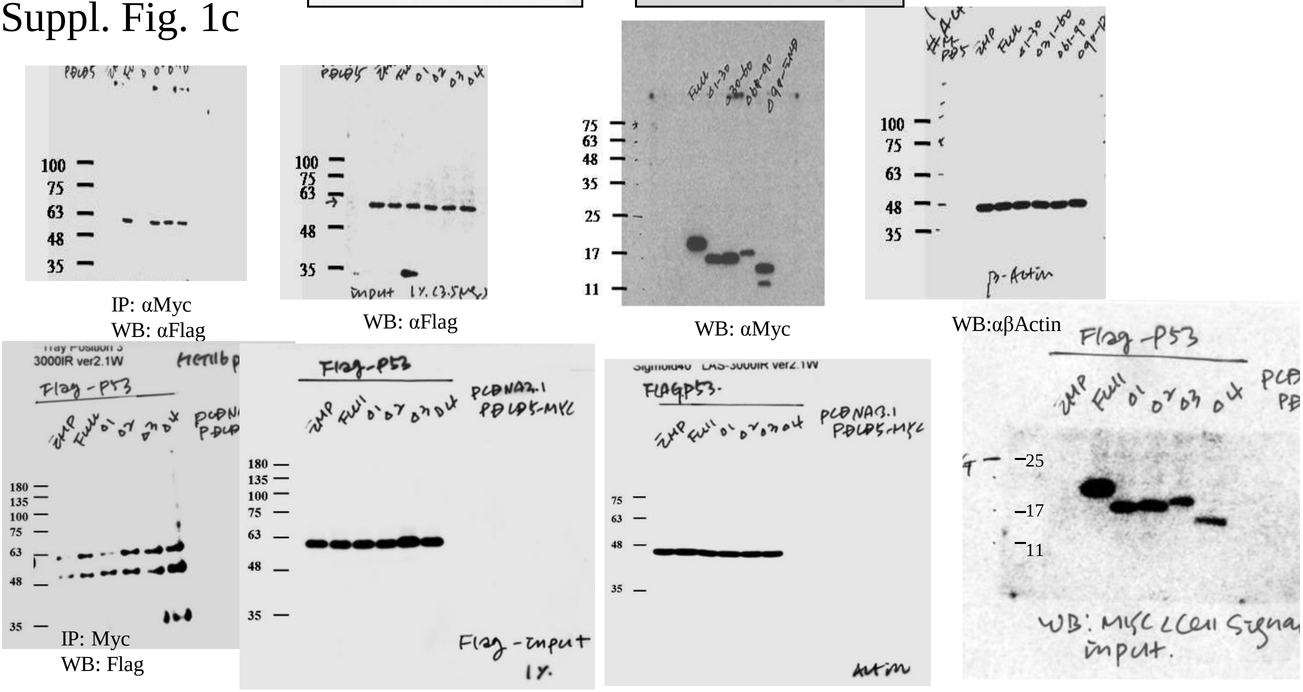

Suppl. Fig. 1d

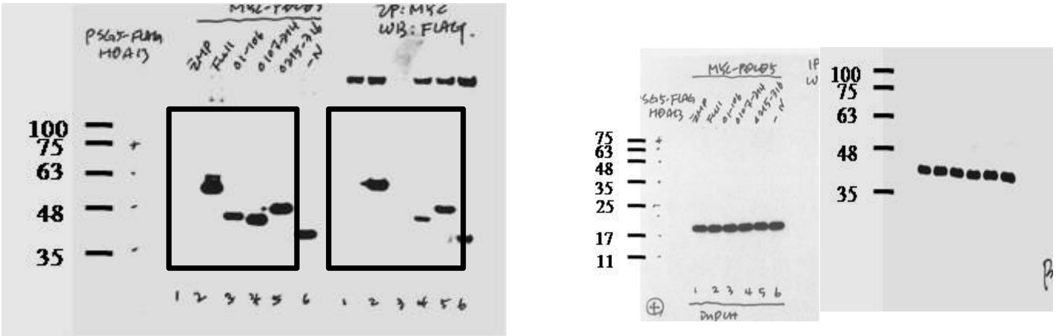

WB: αFlag

IP: αMyc
WB: αFlag

WB: αMyc

WB:αβActin

Suppl. Fig. 2a

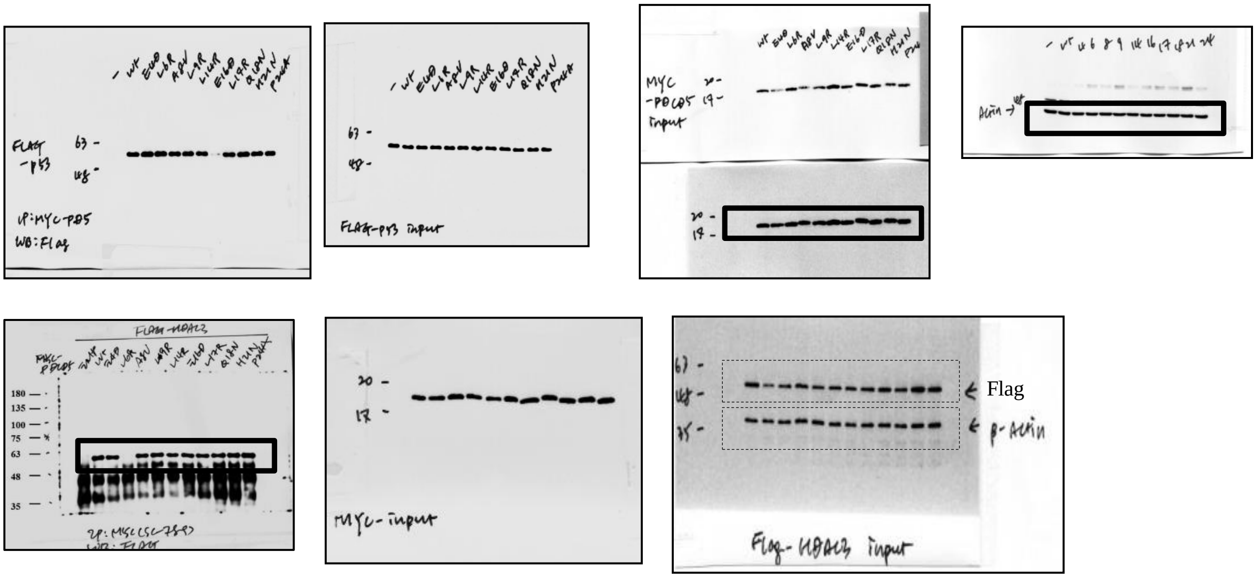

Suppl. Fig. 3a

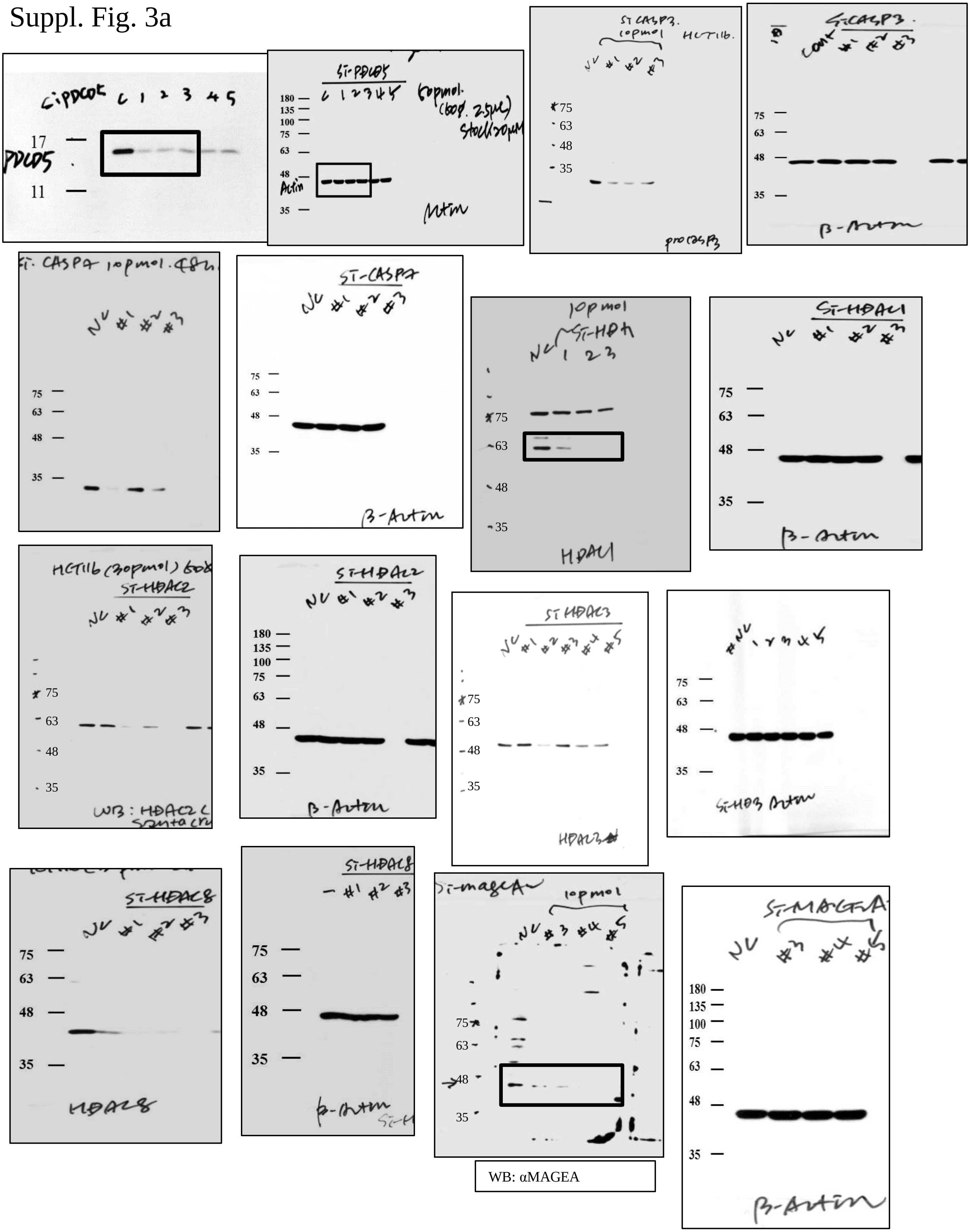

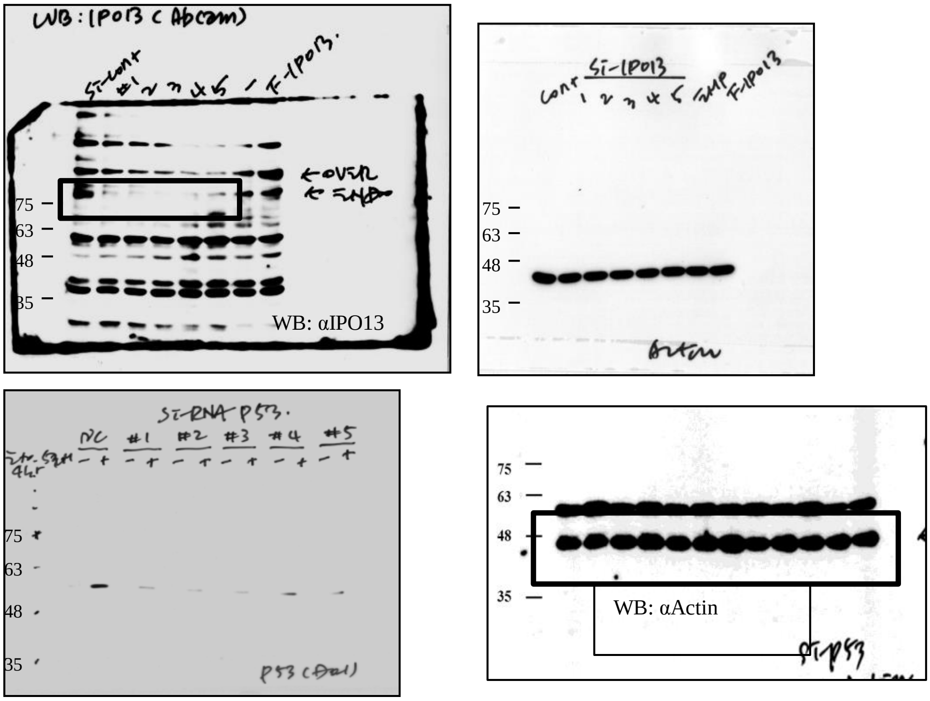
WB: αIPO13
75
63
48
35
WB: αActin

Suppl. Fig. 4

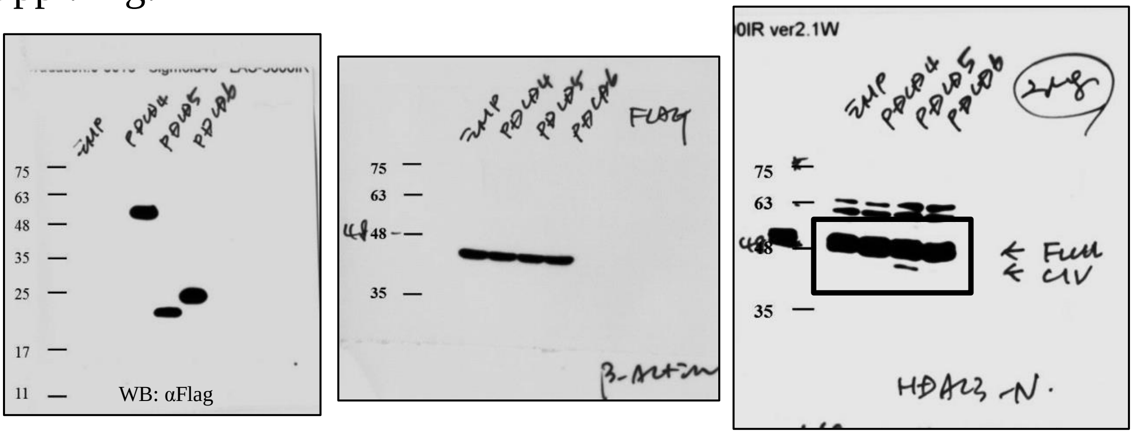
75
63
48
35
25
17
11
WB: αFlag
0IR ver2.1W

Suppl. Fig. 5a

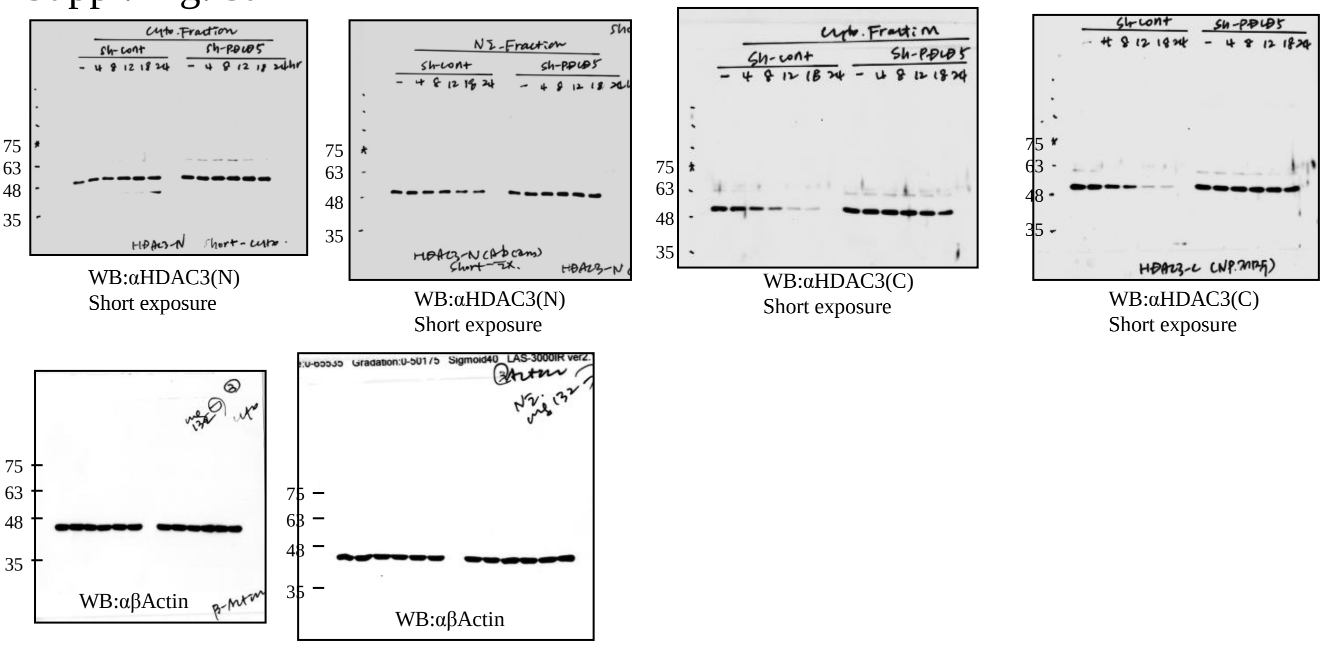

Suppl. Fig. 5b

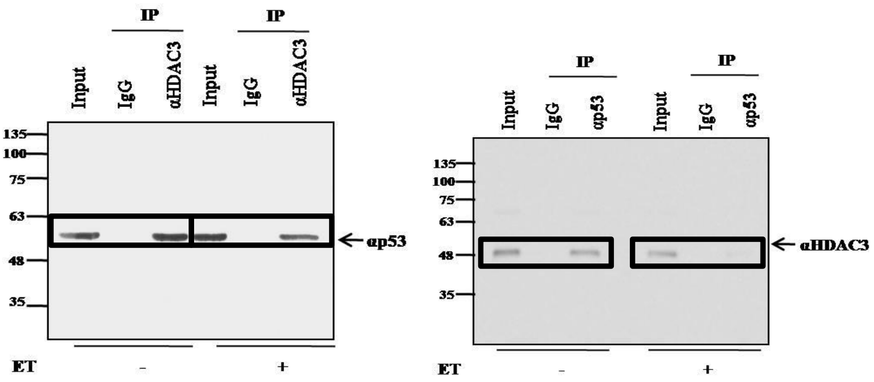

Suppl. Fig. 6a

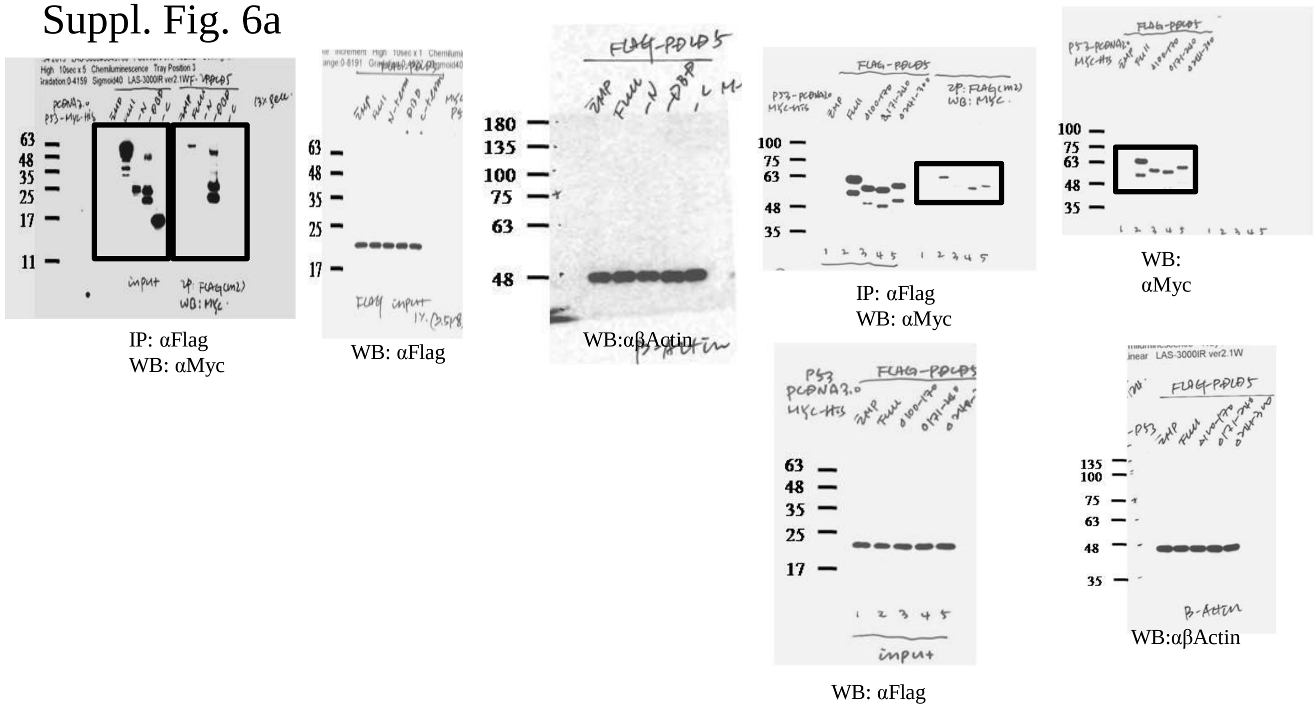

Suppl. Fig. 6b

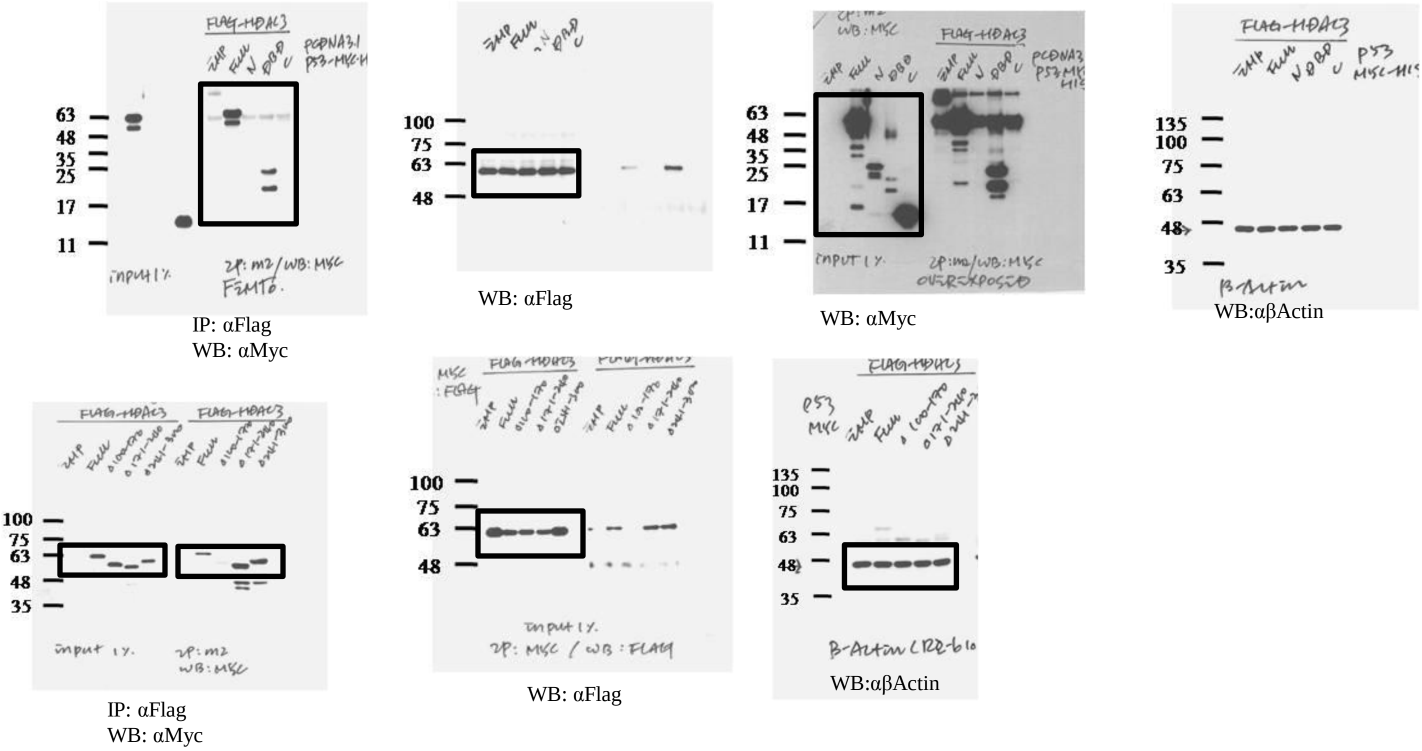

Suppl. Fig. 7a

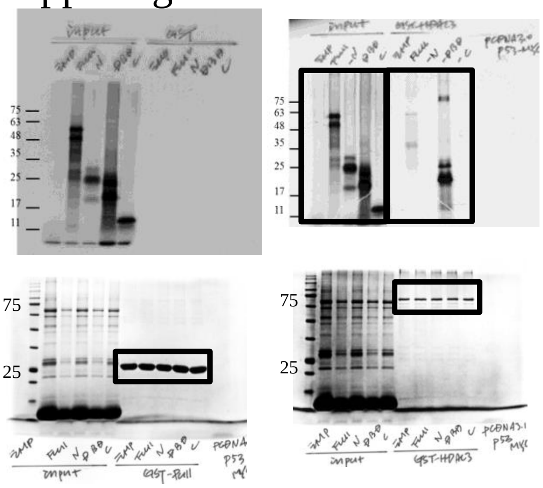

Suppl. Fig. 7b

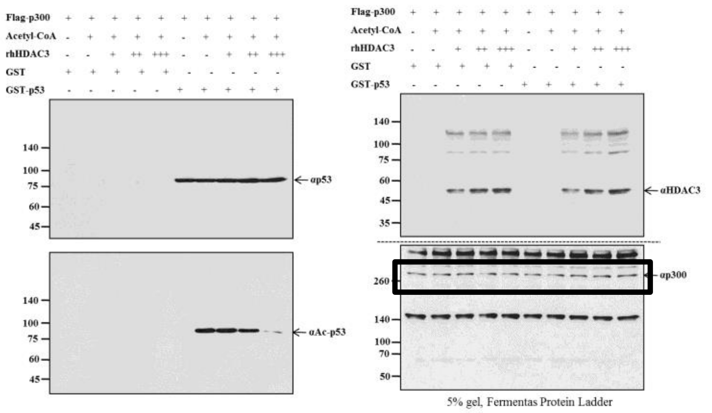

Suppl. Fig. 7c

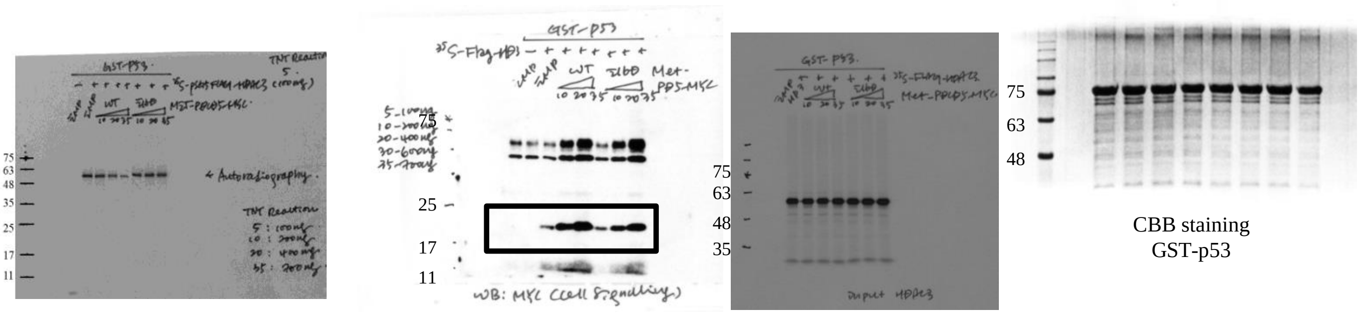

Suppl. Fig. 8a

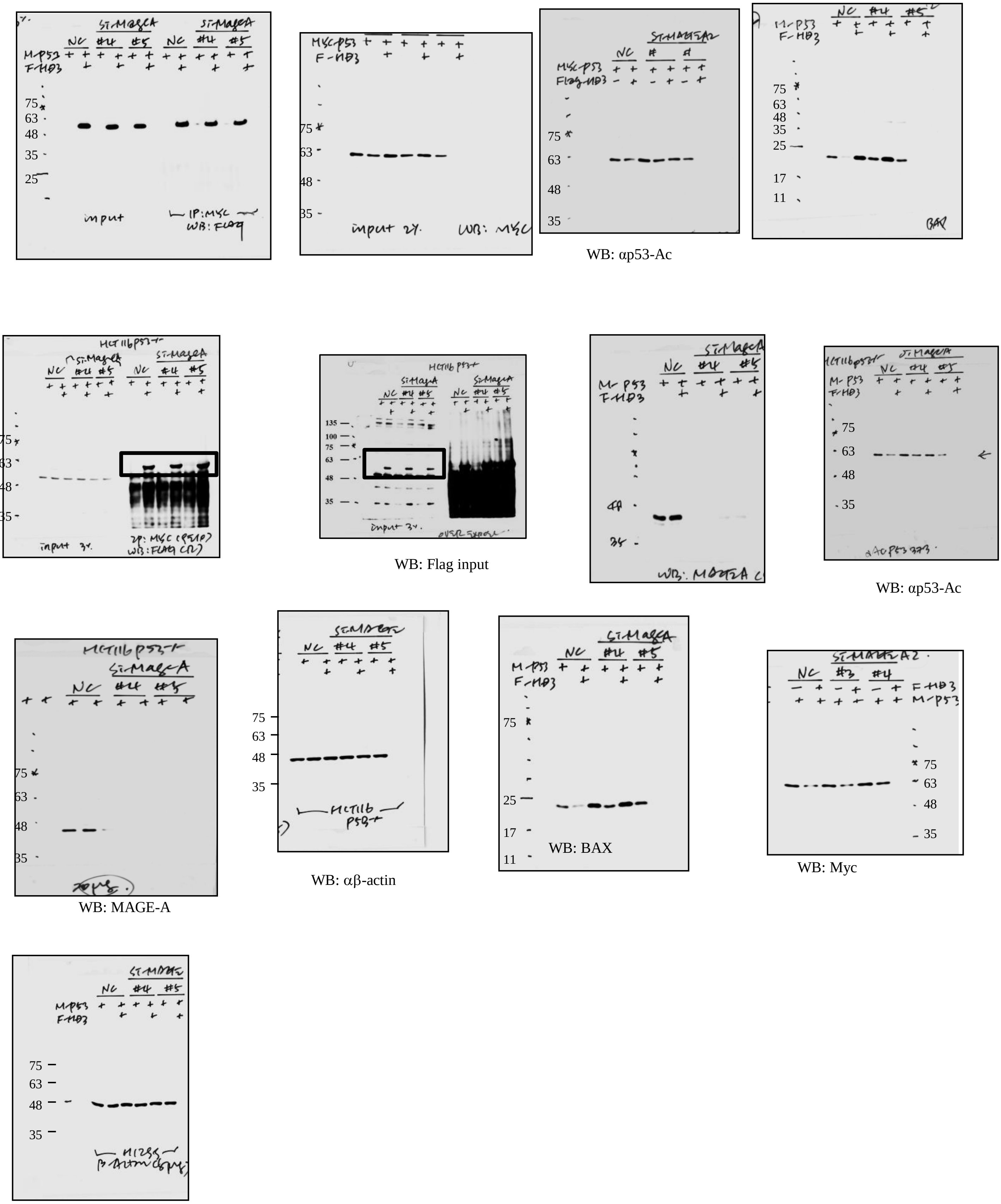

WB: αp53-Ac

WB: Flag input

WB: αp53-Ac

WB: MAGE-A

WB: αβ-actin

WB: BAX

WB: Myc

WB: αβ-actin

Suppl. Fig. 8b

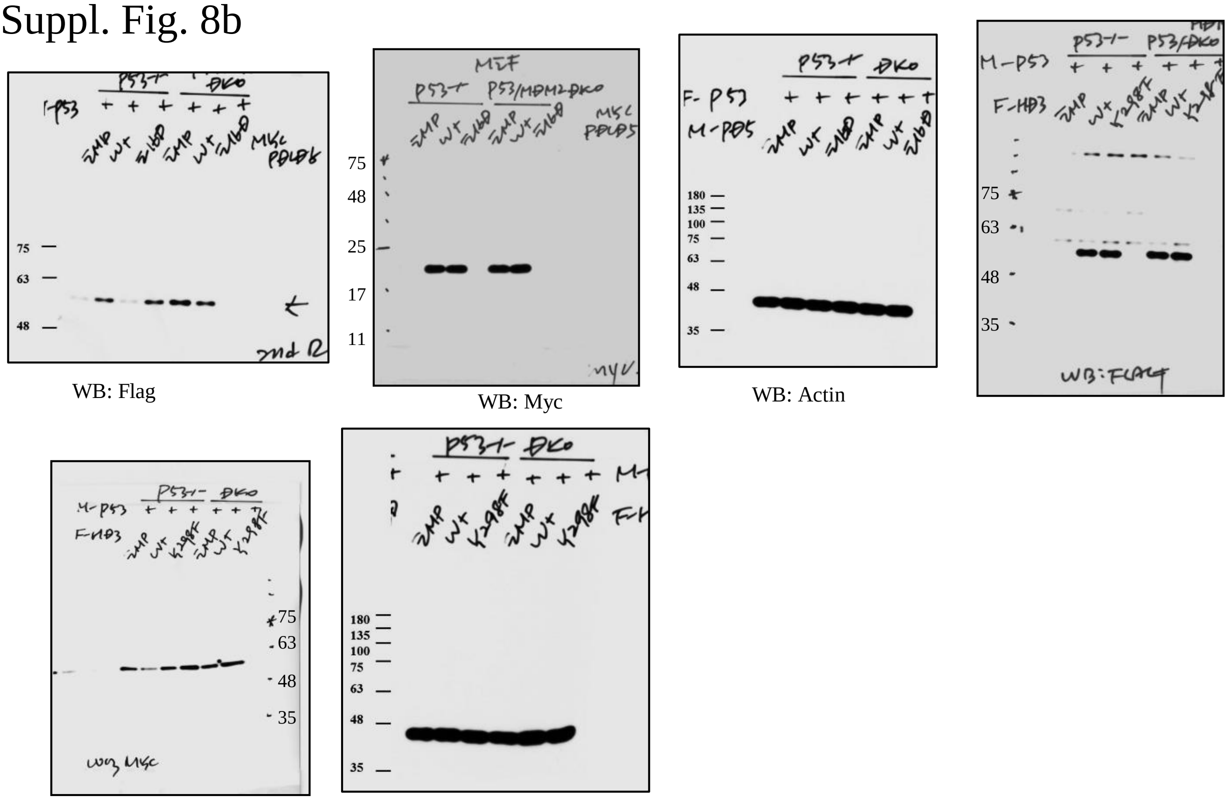

WB: Flag

WB: Myc

WB: Actin

WB: αMyc

WB: αβ-actin

Suppl. Fig. 9a

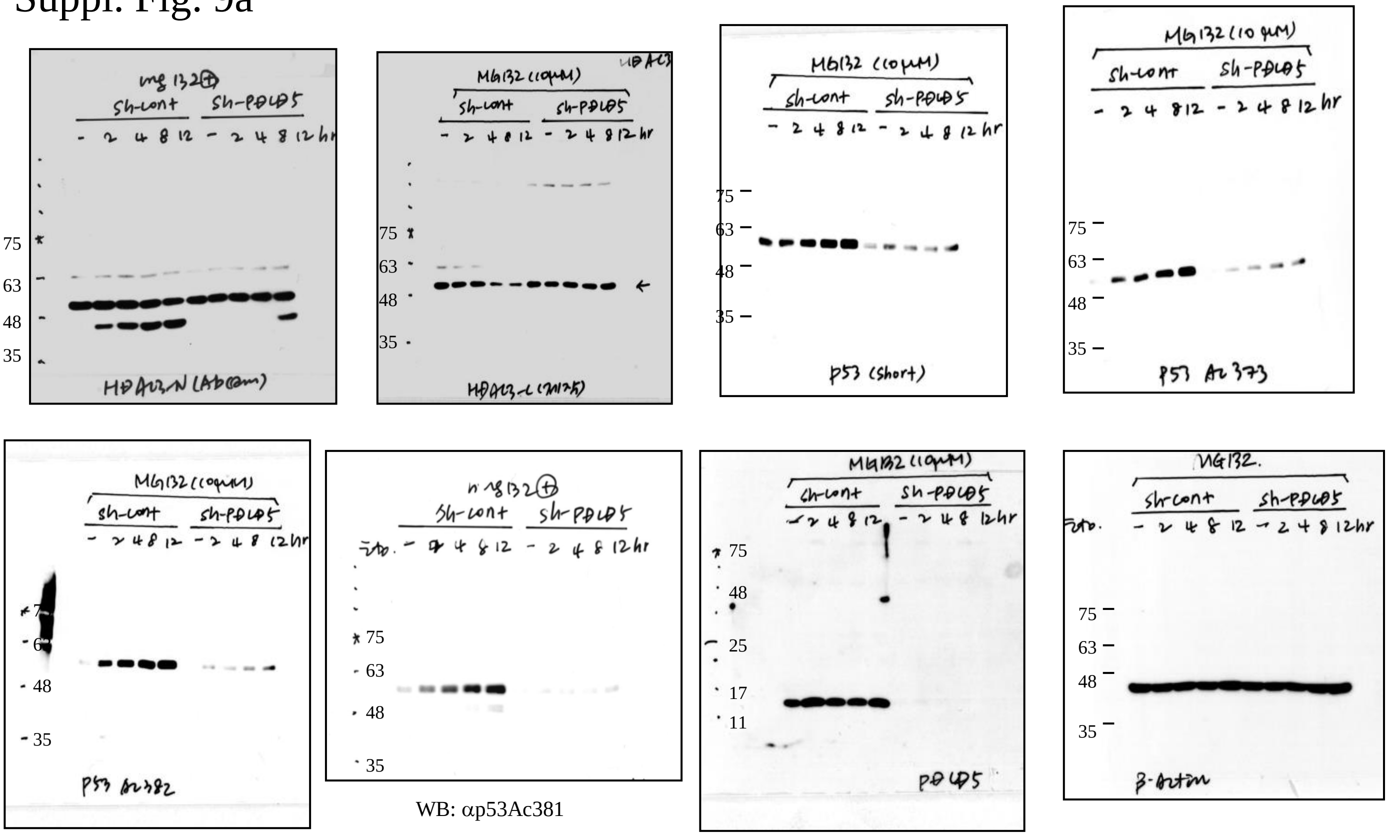

WB: αp53Ac381

Suppl. Fig. 9c

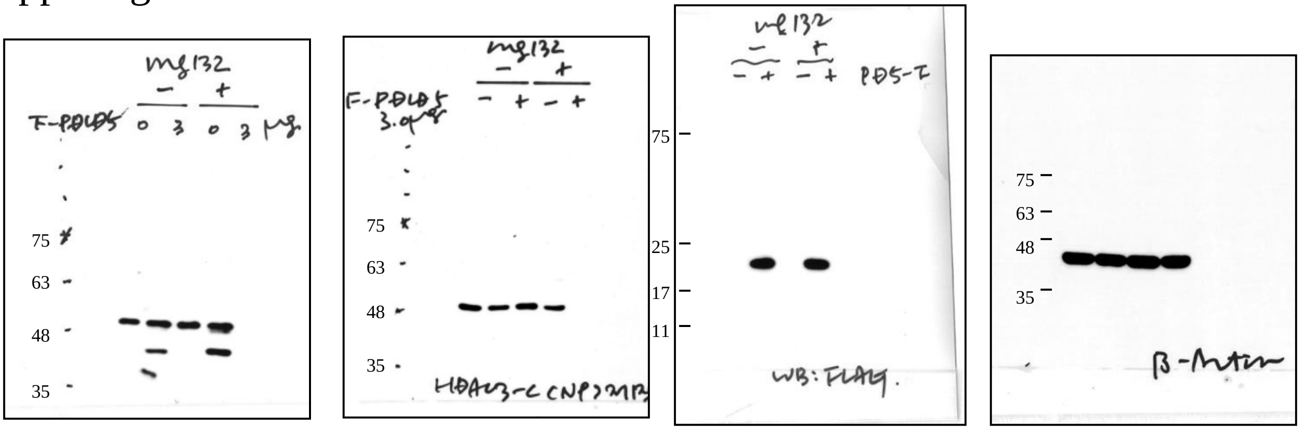

WB: αHDAC3(N)

Suppl. Fig. 10a

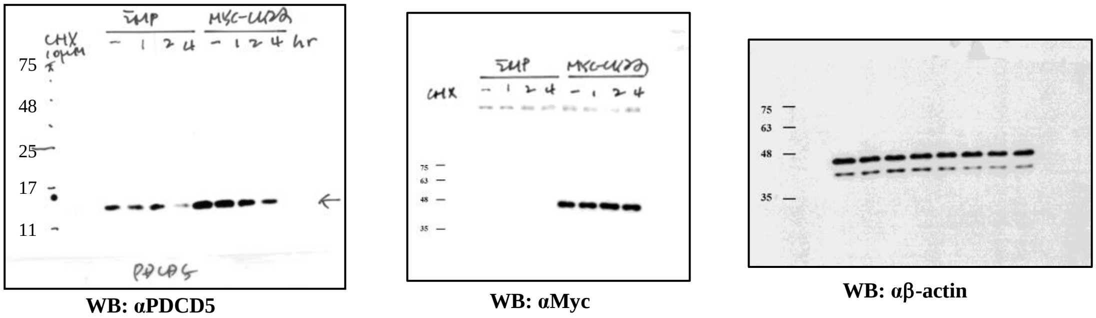

WB: αPDCD5

WB: αMyc

WB: αβ-actin

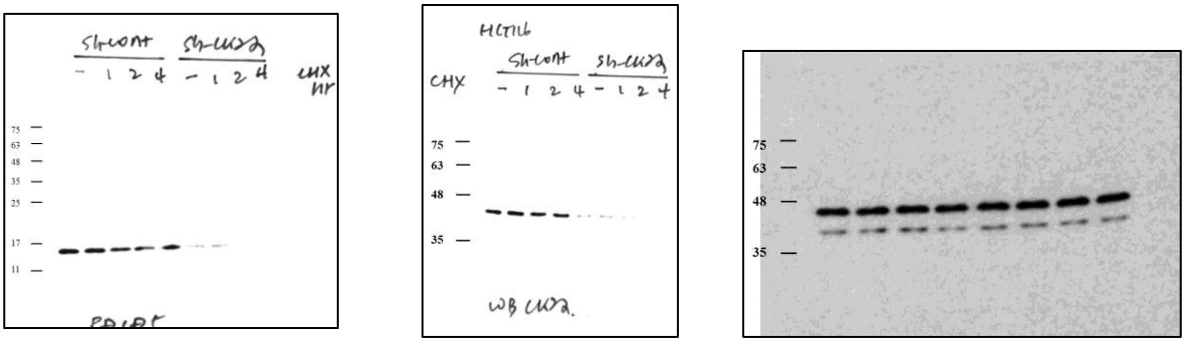

WB: αPDCD5

WB: αCK2α

WB: αβ-actin

Suppl. Fig. 10b

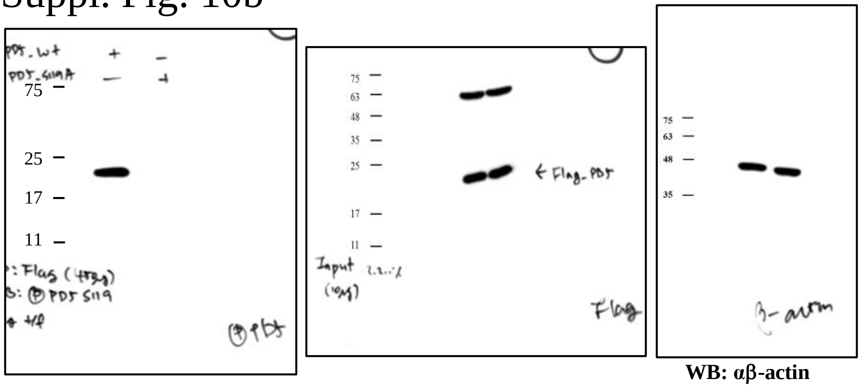

IP: αFlag
WB: αpPDCD5(S119)

WB: αFlag

WB: αβ-actin

Suppl. Fig. 10c

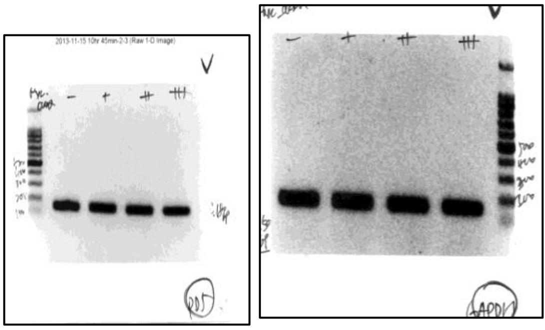

Suppl. Fig. 10d

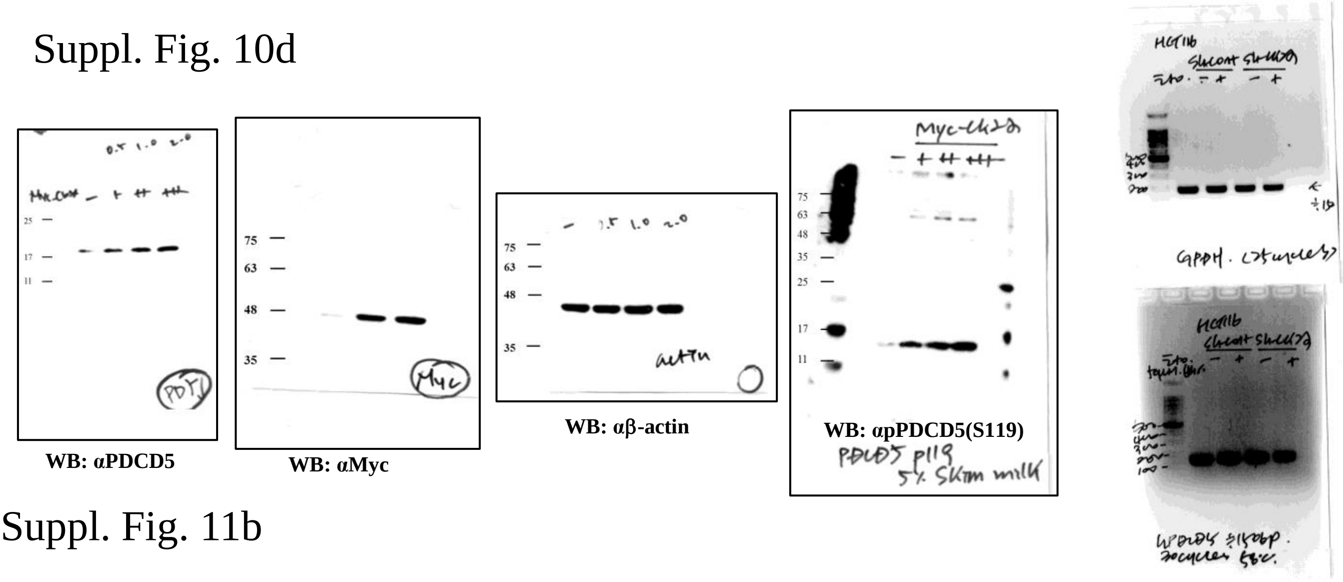

WB: αPDCD5

WB: αMyc

WB: αβ-actin

WB: αpPDCD5(S119)

Suppl. Fig. 11b

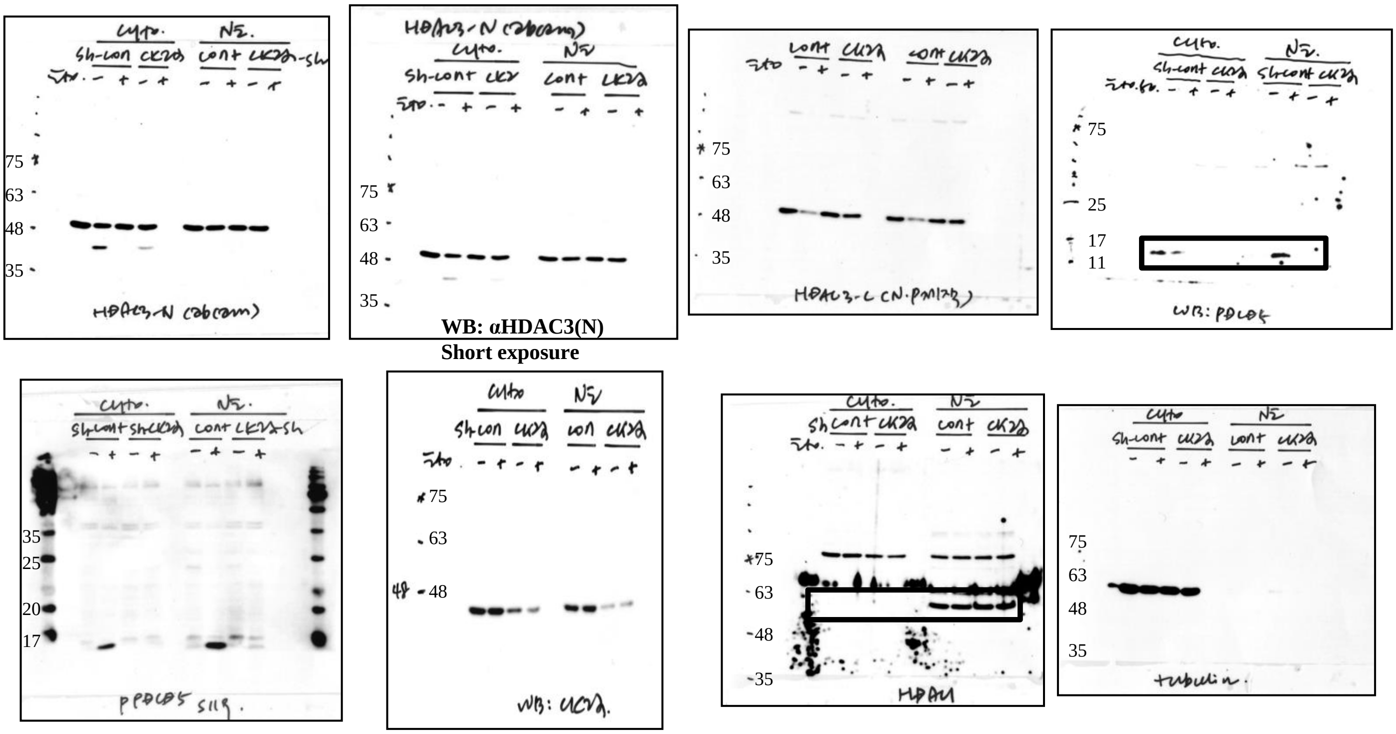

WB: αHDAC3(N)
Short exposure

Suppl. Fig. 11c

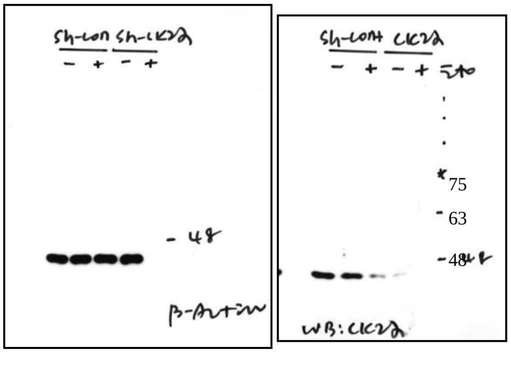

Suppl. Fig. 11d

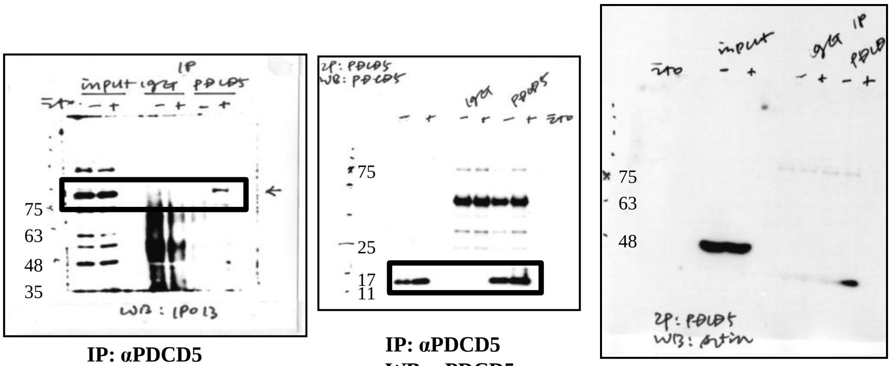

IP: αPDCD5
WB: αIPO13

IP: αPDCD5
WB: αPDCD5

IP: αPDCD5
WB: αβActin

Suppl. Fig. 11e

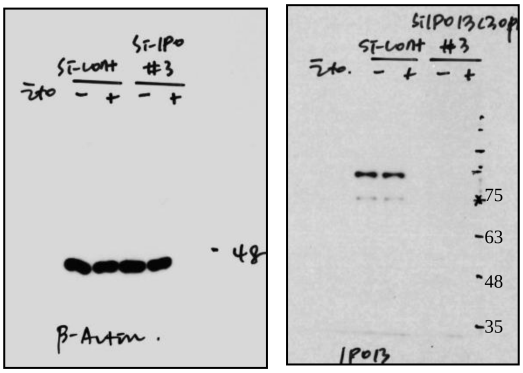

Suppl. Fig. 11f

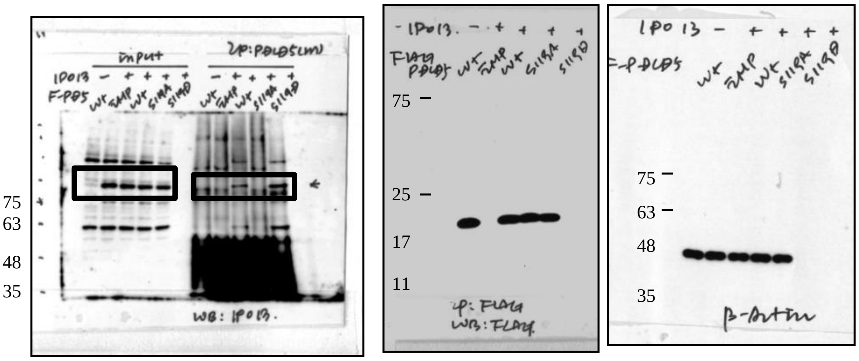

Suppl. Fig. 14b

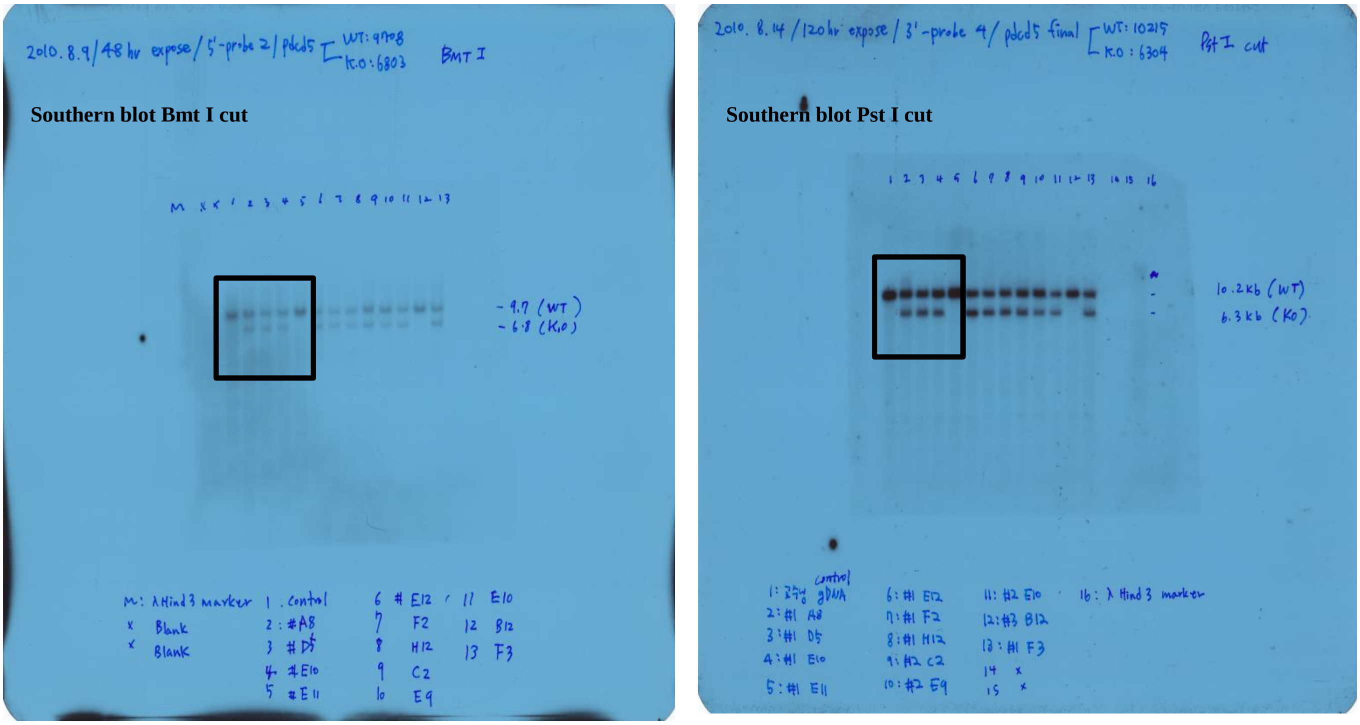

Suppl. Fig. 14d

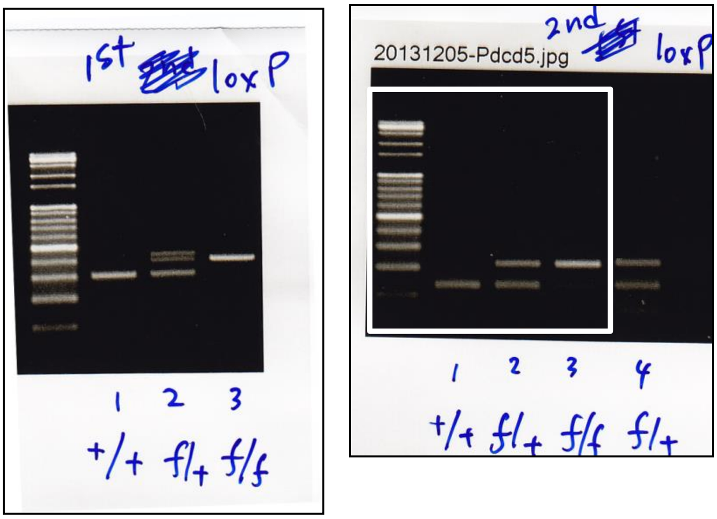

Suppl. Fig. 15b

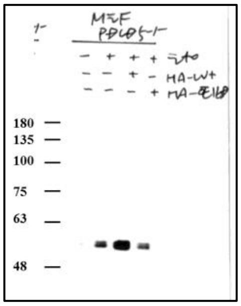

WB: p53

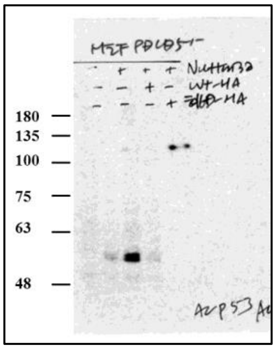

WB: p53-Ac

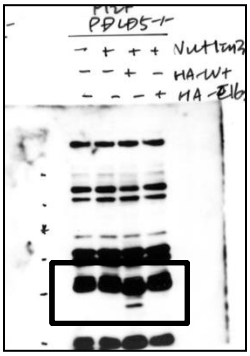

WB: HDAC3-N

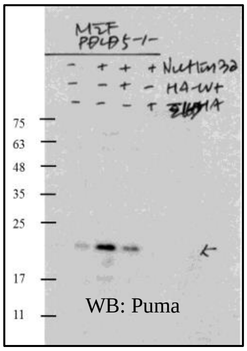

WB: Puma

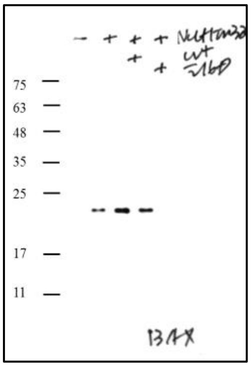

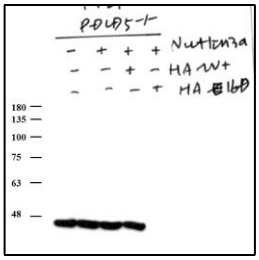

WB: Actin

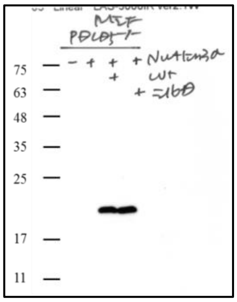

WB: HA

Suppl. Fig. 16a

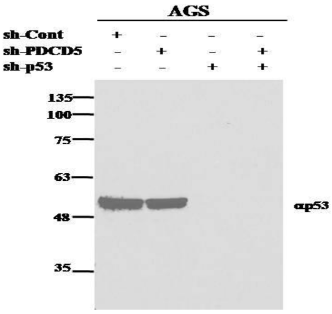

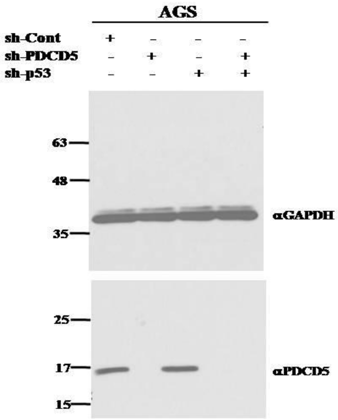

Suppl. Fig. 16f

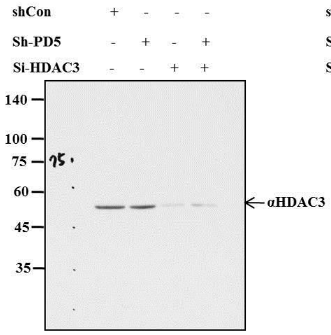

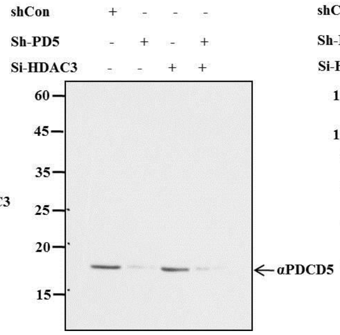

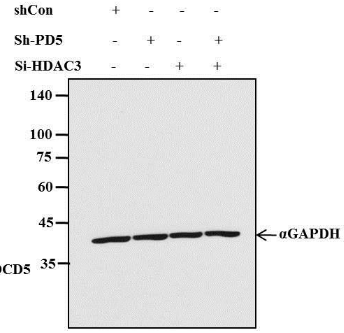

Supplementary Table 1. Screening of HDAC3-interacting proteins by yeast-two hybrid assay

No	Gene Symbol	Gene Bank
1	cAMP RESPONSE ELEMENT-BINDING PROTEIN 3 (CREB3)	AAO33070
2	FIBROBLAST GROWTH FACTOR RECEPTOR 1 (FGFR1)	AAH15035
3	HIGH MOBILITY GROUP NUCLEOSOMAL BINDING PROTEIN 3 (HMGN3)	BC009529
4	PROTEIN-TYROSINE PHOSPHATASE, RECEPTOR-TYPE, ALPHA (PTPRA)	BC027308
5	FIBRONECTIN TYPE III AND SPRY DOMAINS-CONTAINING PROTEIN 1 (FSD1)	AF316829
6	**Programmed Cell Death 5 (PDCD5)**	**CAG33215**
7	AMYLOID BETA A4 PRECURSOR-LIKE PROTEIN 1(APLP1)	BC012889
8	DNAJ/HSP40 HOMOLOG, SUBFAMILY B, MEMBER 1 (DNAJB1)	CAG46478
9	CCR4-NOT TRANSCRIPTION COMPLEX, SUBUNIT 1 (CNOT1)	ABQ66268
10	ZINC FINGER PROTEIN 184 (ZNF184)	BC022992
11	ACTIVATING TRANSCRIPTION FACTOR 7-INTERACTING PROTEIN (ATF7IP)	BC063855
12	METHYL-CpG-BINDING DOMAIN PROTEIN 6 (MBD6)	BC065530
13	SERINE/THERONINE KINASE PIM2 (PIM2)	NM_006866
14	BROMODOMAIN-CONTAINING PROTEIN 7 (BRD7)	BC050728
15	CASEIN KINASE 2 (CK2)	AY588245
16	UBIQUITIN A-52-RESIDUE RIBOSOMAL PROTEIN FUSION PRODUCT (UBA52)	AF348700
17	THYMOSIN, BETA-4, X CHROMOSOME (TMSB4X)	BM760483
18	UBIQUITIN-LIKE 4A (UBL4A)	NP_055050
19	L3MBT-LIKE 2 (L3MBTL2)	BC017191
20	ECTODERMAL-NEURAL CORTEX 1 (ENC1)	BC000418
21	MUS81, S. CEREVISIAE, HOMOLOG OF (MUS81)	BC009999
22	MINICHROMOSOME MAINTENANCE, S. CEREVISIAE, HOMOLOG OF, 7 (MCM7)	BC013375
23	RAS-ASSOCIATED PROTEIN RAB3A (RAB3A)	BC011782

Supplementary Table 2. Screening of PDCD5-interacting proteins by LC-MS/MS analysis

No	Gene Symbol	Gene Bank	Z-score*	Total Spectra count (TSC)**
1	Chaperonin containing TCP1, subunit 6B (zeta 2) (CCT6B)	NM_006584	12.97	9
2	WD repeat domain 74 (WDR74)	NM_018093	13.27	2
3	Crystallin, mu (CRYM)	NM_001888	13.27	2
**4**	**Histone deacetylase 3 (HDAC3)**	**NM_003883**	**12.38**	**1**
5	Inositol polyphosphate-5-phosphatase J (INPP5J)	NM_001284285	9	1
6	Chaperonin containing TCP1, subunit 4 (delta) (CCT4)	NM_006430	7.06	65
7	Tubulin, gamma 1 (TUBG1)	NM_001070	6.74	3
8	Chaperonin containing TCP1, subunit 6A (zeta 1) (CCT6A)	NM_001762	6.64	62
9	Chaperonin containing TCP1, subunit 3 (gamma) (CCT3)	NM_005998	6.59	75
10	Chaperonin containing TCP1, subunit 7 (eta) (CCT7)	NM_006429	6.46	85
11	Chaperonin containing TCP1, subunit 5 (epsilon) (CCT5)	NM_012073	5.93	92
12	Chaperonin containing TCP1, subunit 8 (theta) (CCT8)	NM_006585	4.56	59
13	T-complex 1 (TCP1)	NM_030752	4.42	51
14	Poly(rC) binding protein 3 (PCBP3)	NM_020528	4.17	1
15	DDB1 associated factor 7 (DCAF7)	NM_005828	3.2	2
16	Amylo-alpha-1, 6-glucosidase, 4-alpha-glucanotransferase (AGL)	NM_000642	1.33	14
17	DnaJ (Hsp40) homolog, subfamily C, member 10 (DNAJC10)	NM_018981	1.28	10
18	Small nuclear ribonucleoprotein 40kDa (U5) (SNRNP40)	NM_004814	0.84	1
19	TRK-fused gene (TFG)	NM_006070	0.79	48
20	Desmoglein 1 (DSG1)	NM_001942	0.46	3
21	Myosin, heavy chain 10, non-muscle (MYH10)	NM_001256012	0.3	1
22	TATA box binding protein (TBP)-associated factor, RNA polymerase I, C, 110kDa (TAF1C)	NM_005679	0.24	1
23	Fused in sarcoma (FUS)	NM_004960	-0.14	14
24	DEAD (Asp-Glu-Ala-Asp) box polypeptide 3, X-linked (DDX3X)	NM_001356	-0.18	13
25	Heat shock 70kDa protein 1B (HSPA1B)	NM_005346	-0.78	32
26	Heat shock 70kDa protein 8 (HSPA8)	NM_006597	-0.8	31

*Z-score is representative of the identification of candidate interacting proteins.
**TSC is for each identified protein from each IP-MS/MS experiment.

Supplementary Table 3. List of the antibodies used in the study

Antibody	Vendor	Cat. No.	WB	IP	IF	ChIP	Etc.
**HDAC1**	Santa Cruz	sc-7872	1/1000				
**HDAC2**	Santa Cruz	sc-7899	1/1000				
**HDAC8**	Santa Cruz	sc-11405	1/1000				
**HDAC3**	Santa Cruz	sc-11417	1/1000	1/100	1/100		
**caspasse-8 (pro)**	Santa Cruz	sc-7272	1/1000				
**caspasse-9 (pro)**	Santa Cruz	sc-17784	1/1000				
**p53**	Santa Cruz	sc-126	1/3000	1/500		1μg	
**p53**	Santa Cruz	sc-6243	1/1000	1/100			For mouse
**caspase-3 (pro)**	Santa Cruz	sc-7148	1/1000				
**caspase-7 (pro)**	Santa Cruz	sc-56063	1/1000				
**p21**	Santa Cruz	sc-397	1/1000				
**HA**	Santa Cruz	sc-805	1/1000	1/100		2μg	
**MAGE-A2**	Santa Cruz	sc-130164	1/500				
**PDCD5**	Proteintech	12456-1-AP	1/1000	1/100		2μg	
**PDCD5**	Abcam	ab126213			1/200		
**FLAG**	Sigma	F3165	1/10000		1/1000	0.5μg	
**Myc**	Cell signaling	#2278S	1/1000				
**Myc**	Santa Cruz	sc-789		1/100			
**caspase-12 (pro)**	Santa Cruz	sc-70227	1/1000				
**β-Actin**	Sigma	A5441	1/10000				
**acetyl-p53^{K379}**	Cell signaling	#2570	1/1000				
**acetyl-p53^{K382}**	Cell signaling	#2525S	1/1000				
**caspase-3 (active)**	Cell signaling	#9116S	1/1000				
**α-tubulin**	Millipore	05-829	1/10000				
**GAPDH**	Millipore	CB1001	1/10000				
**p300**	Millipore	05-257	1/1000			2μg	
**PARP-1**	BD Pharming en	51-6639GR	1/1000				
**acetyl-p53^{K373}**	Epitomics	#2204-1	1/1000			3μg	mouse K370
**Bax**	Epitomics	1063-1	1/1000				
**PDCD4**	Abcam	ab32831-100	1/1000				
**PDCD6**	Abcam	ab133326	1/1000				
**IPO13**	Abcam	ab101374	1/500				
**acetyl-p53^{K381}**	Abcam	ab61241	1/1000				
**Puma**	Abcam	ab9643	1/1000				
**Ck2α**	upstate	#06-873	1/2000				

WB, Western blot; IP, Immunoprecipitation; IF, Immunoflurorecence; ChIP, Chromatin IP

Supplementary Table 4. List of the sequences used for in the study

	Sequence	
Gene name	**Sense**	**Anti-sense**
**siPDCD5-1**	5’-GCAAGAUAUGGACAACUAAUU-3’	5’ –UUAGUUGUCCAUAUCUUGCUU- 3’
**siPDCD5-2**	5’-GUAACUUAGCACUUGUAAAUU-3’	5’ –UUUACAAGUGCUAAGUUACUU- 3’
**siPDCD5-3**	5’-GCAGAAAUGAGAAACAGUAUU-3’	5’ –UACUGUUUCUCAUUUCUGCUU- 3’
**siCaspase-3-2**	5’-GAGAUGGGUUUAUGUAUAAUU-3’	5’ –UUAUACAUAAACCCAUCUCAG- 3’
**siCaspase-3-3**	5’-GGCCUGCCGUGGUACAGAAUU- 3’	5’ – UUCUGUACCACGGCAGGCCUG- 3’
**siCaspase-7-1**	5’-GGGCAAAUGCAUCAUAAUAUU-3’	5’ –UAUUAUGAUGCAUUUGCCCAG- 3’
**siCaspase-7-3**	5’-GGAACUCUACUUCAGUCAAUU-3’	5’ – UUGACUGAAGUAGAGUUCCUU- 3’
**siHDAC1-2**	5’-GCUUCAAUCUAACUAUCAAUU-3’	5’- UUGAUAGUUAGAUUGAAGCAA- 3’
**siHDAC1-3**	5’-CGACUGUUUGAGAACCUUAUU-3’	5’ –UAAGGUUCUCAAACAGUCGCU- 3’
**siHDAC2-2**	5’-CAGUGAUGAGUAUAUCAAAUU-3’	5’- UUUGAUAUACUCAUCACUGUG- 3’
**siHDAC2-3**	5’-GGUCAAUAAGACCAGAUAAUU-3’	5’ –UUAUCUGGUCUUAUUGACCGU- 3’
**siHDAC3-2**	5’-CAACAAGAUCUGUGAUAUUUU-3’	5’- AAUAUCACAGAUCUUGUUGUU- 3’
**siHDAC3-4**	5’-CUGACAAUGGUACCUAUUAUU- 3’	5’- UAAUAGGUACCAUUGUCAGUU- 3’
**siHDAC8-2**	5’-CAUUCAGGAUGGCAUACAAUU-3’	5’ –UUGUAUGCCAUCCUGAAUGGG- 3’
**siHDAC8-3**	5’-GCUGGGAGCUGACACAAUAUU-3’	5’ –UAUUGUGUCAGCUCCCAGCUG- 3’
**siMAGE-a2-3**	5’-CUAUAUUGUGAACGAAUUAUU-3’	5’ –UAAUUCGUUCACAAUAUAGUU- 3’
**siMAGE-a2-4**	5’-CUUAUACCUCAGUCUAUUAUU-3’	5’ –UAAUAGACUGAGGUAUAAGUU- 3’
**siMAGE-a2-5**	5’-GAAAUUAAAUCUGAAUAAAUU-3’	5’ –UUUAUUCAGAUUUAAUUUCUU- 3’
**sip53-1**	5’-CAAGCAAUGGAUGAUUUGAUU-3’	5’ –UCAAAUCAUCCAUUGCUUGUU- 3’
**sip53-2**	5’-GGAUGUUUGGGAGAUGUAAUU-3’	5’ –UUACAUCUCCCAAACAUCCUU- 3’
**sip53-3**	5’-GCAGUUAAGGGUUAGUUUAUU-3’	5’ –UAAACUAACCCUUAACUGCUU- 3’
**siIPO13-3**	5’ –CAUUGACGUCAACUAUUCUUU- 3’	5’ –AGAAUAGUUGACGUCAAUGUU- 3’
**siIPO13-4**	5’ –CCCUAUUCCCAAAGAGUAAUU- 3’	5’ –UUACUCUUUGGGAAUAGGGUU- 3’

Continue on next page

	Sequence		
**Gene name**	**Sense**	**Anti-sense**	**Etc.**
**GAPDH (h)**	5’-ATGTTCGTCATGGGTGTGAAC-3’	5’-GCATGGACTGTGGTCATGAGT-3’	**qRT-PCR**
**GAPDH(m)**	5’-GTGTTCCTACCCCCAATGTGT-3’	5’-AGGAGACAACCTGGTCCTCAGT-3’	
**Bax (h)**	5’-TCTACTTTGCCAGCAAACTGGTGC-3’	5’-TGTCCAGCCCATGATGGTTCTGAT-3’	
**Bax (m)**	5’-GGAGCAGCTTGGGAGCG-3’	5’-AAAAGGCCCCTGTCTTCATGA-3’	
**Puma (h)**	5’-ACTGTGAATCCTGTGCTCTGCC-3’	5’-CAAATGAATGCCAGTGGTCACAC-3’	
**Caspase3 (h)**	5'-CAT GGA AGC GAA TCA ATG GAC T-3'	5'-CTG TAC CAG ACC GAG ATG TCA-3'	
**HDAC1 (h)**	5'-CGC CCT CAC AAA GCC AAT G-3'	5'-CTG CTT GCT GTA CTC CGA CA-3'	
**HDAC3 (h)**	5'-CCT GGC ATT GAC CCA TAG CC-3'	5'-CTC TTG GTG AAG CCT TGC ATA-3'	
**PDCD5 (h)**	5'-AAA GCA CAG GGA AGC AGA AA-3'	5'-TTG TCC ATA TCT TGC CAT CTG-3'	
**p53 (h)**	5'-ACC TAT GGA AAC TAC TTC CTG AAA-3'	5'-CTG GCA TTC TGG GAG CTT CA-3'	
**Puma (m)**	5’-GCCTCCTTTCTCACCGAGC-3’	5’-GGCCGCAATCTTTGCA-3’	
**Caspase7 (h)**	5'-CGG TCC TCG TTT GTA CCG TC-3'	5'-CGC CCA TAC CTG TCA CTT TAT CA-3'	
**HDAC2 (h)**	5'-ATG GCG TAC AGT CAA GGA GG-3'	5'-TGC GGA TTC TAT GAG GCT TCA-3'	
**HDAC8 (h)**	5'-TCG CTG GTC CCG GTT TAT ATC-3'	5'-TAC TGG CCC GTT TGG GGA T-3'	
**MAGE-A (h)**	5'-AAG GTA AGA CGC CGA GGG A-3'	5'-ACT CCC TGA GAT GCC AGA GTT-3'	
**IPO13 (h)**	5’-GTC ACC TCC GTC ACA CTC AC -3’	5’- CCA ATC GTT CAC ACA GGA AC -3’	
**Bax (h)**	5’-TAATCCCAGCGCTTTGGAAG-3’	5’-TGCAGAGACCTGGATCTAGCAA-3’	**ChIP assay**
**Bax (m)**	5’-CTGTCCTTGAACTCAGAGAGATGG-3’	5’-GGCTATCCTGGAACTCACTTTTGA-3’	
**Puma (h)**	5’-CTGTGGCCTTGTGTCTGTGAGTAC-3’	5’-CCTAGCCCAAGGCAAGGAGGAC-3’	
**Puma (m)**	5’-CCTCTGGCTGCCGGGAAACCCCCC-3’	5’-CGCCCCGCCTCTCGCTGGCTCC-3’	
**PDCD5 ∆1-30**	5’ – ACC TCT AGA GAA TTC GAA GCA AAG CAC AGG -3’	5’- CCT GTG CTT TGC TTC GAA TTC TCT AGA GGT -3’	**SDM**
**PDCD5 ∆31-60**	5’- GAT GCG GCC CAA CAG CTT GTA AAG CCT GAA-3’	5’- TTC AGG CTT TAC AAG CTG TTG GGC CGC ATC -3’	
**PDCD5 ∆61-90**	5’- TTA AGT AAC TTA GCA GGT TTA ATA G AA ATC -3’	5’- GAT TTC TAT TAA ACC TGC TAA GTT ACT TAA -3’	
**PDCD5 ∆91-124**	5’ –AAG CTA TCA GAA CAA TGA GCT AGC TAG GTA -3’	5’- TAC CTA GCT AGC TCA TTG TTC TGA TAG CTT -3’	
**PDCD5 L6R**	5’- ATG GCG GAC GAG GAG CGT GAG GCG CTG AGG AG -3’	5’- CT CCT CAG CGC CTC ACG CTC CTC G TC CTC CAT -3’	
**PDCD5 E16D**	5’- AGA CAG AGG CTG GCC GAC CTG CAG GCC AAA CAC -3’	5’– GTG TTT GGC CTG CAG GTC GGC CA G CCT CTG TCT -3’	

(h), human; (m), mouse; SDM, site-direct mutagenesis

Supplementary Table 5. The information on clinic-pathological characteristics of patients

Characteristic	YUSH (n=78)
**AGE**	
Median	60
Range	28-86
**Sex (%)**	
Male	50(64.1%)
Female	28(35.9%)
**Subsite of tumor**	
Cardia	6(7.7%)
Body	23(29.5%)
Antrum	33(42.3%)
Fundus	2(2.6%)
Unknown	14(17.9%)
**Histologic Type of tumor (%)**	
Intestinal	12(15.4%)
Diffuse	12(15.4%)
Mixed	2(2.6%)
Unknown	52(66.6%)
**Cancer Stage, TNM class(%)**	
I	17(21.8%)
II	50(64.1%)
III	2(2.6%)
Unknown	9(11.5%)
